# Childhood predictors of health limitations in life across 22 countries: a cross-national and cross-sectional analysis

**DOI:** 10.1186/s44263-025-00188-0

**Published:** 2025-08-13

**Authors:** Jason Paltzer, Elizabeth Kwon, Chukwuemeka N. Okafor, R. Noah Padgett, Erik W. Carter, Jessica Benfer, Tyler J. VanderWeele, Byron R. Johnson

**Affiliations:** 1 The Meros Center, Wisconsin Lutheran College, Milwaukee, WI USA; 2 Department of Public Health, Baylor University, Waco, TX USA; 3 Department of Medicine, Division of Infectious Diseases, Long School of Medicine, University of Texas Health Science Center San Antonio, San Antonio, TX USA; 4 Department of Epidemiology, Harvard T.H. Chan School of Public Health, Cambridge, MA USA; 5 Baylor Center for Developmental Disabilities, Baylor University, Waco, TX USA; 6 Departments of Epidemiology and Biostatistics, Harvard T.H. Chan School of Public Health, Cambridge, MA USA; 7 Institute for Studies of Religion, Baylor University, Waco, TX USA; 8 Human Flourishing Program, Harvard University, Boston, MA USA

**Keywords:** Health limitations, Physical health, Global Flourishing Study, Global health, Epidemiology, Psychological well-being

## Abstract

**Background:**

Preventing health problems that limit access to age-appropriate opportunities and relationships (health limitations) is critical to promoting human flourishing. Understanding childhood correlates of health limitations provides a vantage point for prevention efforts. Thus, the purpose of this study was to examine the childhood predictors of health limitations across diverse countries. An individual’s self-reported health limitations in adulthood are likely to vary by country, reflecting the influence of diverse sociocultural, economic, religious, and health contexts that characterize each nation.

**Methods:**

We used data from the Global Flourishing Study, an ongoing 5-year longitudinal study of human flourishing among 202,898 individuals across 22 countries within nationally representative sampling. A Poisson regression model was fit within each country by regressing health problems in adulthood on a set of childhood predictors. We conducted random effects meta-analyses of the regression coefficients to estimate proportions of effects across countries. Our exploratory analysis highlights key early-life experiences, personal attributes, and familial or social circumstances that are associated with self-reported health limitations in adulthood.

**Results:**

Early predictors of self-reported health limitations in adulthood vary by country, reflecting the influence of diverse sociocultural, economic, religious, and health contexts that characterize each nation. Across all countries, childhood abuse, growing up feeling like an outsider in one’s family, and self-rated health growing up were associated with a greater risk of having a health limitation as an adult.

**Conclusions:**

We found common factors among countries and some variations in childhood predictors across the 22 countries. This cross-national study illuminates the role of broader societal factors in shaping the relationship between childhood experiences and health limitations as adults.

**Supplementary Information:**

The online version contains supplementary material available at 10.1186/s44263-025-00188-0.

## Background

Understanding predictors of health limitations, health problems that limit physical functioning, is important for supporting and caring for individuals throughout their lives. Identifying and preventing such predictors can strengthen human flourishing, defined as living the good life and achieving well-being in all areas [1]. According to VanderWeele (2017), domains of flourishing include mental and physical health as well as “happiness and satisfaction, meaning and purpose, character and virtue, close social relationships,” and financial and material stability [1]. Health problems can limit access to the opportunities and relationships that contribute to human flourishing. A person’s environment and life circumstances could also contribute to their perception of flourishing [2]. Contributions to health limitations include musculoskeletal conditions, age, and chronic conditions, including neurological disorders related to cognitive abilities [3]. Seeing, hearing, mobility, communication, cognition, and self-care are common areas of health limitation and are associated with the quality of life for older populations; however, these do not necessarily minimize flourishing [4]. Childhood experiences can substantially affect health outcomes later in life by shaping one’s health behaviors and psychological development [5]. Understanding childhood predictors of health limitations and their impact on well-being later in life is crucial for prevention and for promoting human flourishing.

Childhood predictors such as abuse and neglect are known to determine health outcomes (or limitations) during childhood and as adults [6–8]. Children with health limitations can experience exacerbated functional limitations later in life due to additional required health care, surgeries, therapy, and other interventions that lead to compensatory actions [9, 10]. The relationship between health limitations and flourishing is complex and multi-generational. The ability of a parent to care for a child can lead to psychological delays and physical symptoms, while positive family activities like attending religious services and eating meals together lead to high flourishing [11, 12].

Positive childhood experiences (PCEs) need to be considered, including community volunteering, a guiding mentor, a connected caregiver, a safe neighborhood, a supportive neighborhood, and a resilient family [13]. In a national study in the USA comparing rural and urban PCEs, the environmental context plays a significant role in the presence of PCEs among children, leading to high levels of flourishing [13]. A greater understanding of the influence of cross-cultural and culture-specific childhood predictors on health limitations is needed to promote human flourishing.

Cross-country comparison is essential to understand how cultural, policy, and socioeconomic differences influence the association between childhood predictors and health limitations [14]. Although the existing body of research investigating the association between childhood predictors and health limitations, predominantly conducted in the USA, is valuable, these findings are constrained by certain cultural and socioeconomic factors. By leveraging cross-national data, the current study provides insights into the link between childhood experiences and health limitations across diverse environments.

Variation in the social determinants of health across cultures influences the prevalence of health limitations later in life. For example, higher rates of adolescent pregnancies and poor maternal outcomes in African countries lead to an increased risk for health problems that limit age-appropriate activity [15]. Caste discrimination in India or the prevalence of child labor in some Asian countries represent cultural factors that may influence health limitations in adulthood [16].

The present study of global human flourishing among 202,898 individuals from 22 countries with nationally representative samples provides a greater understanding of the nuances in childhood predictors across cultures and countries influencing health limitations. This study provides an exploratory analysis of key childhood predictors associated with experiencing health problems preventing age-appropriate functioning later in life. The childhood predictors were selected based on a multi-step process described in Lomas et al. [17]. This study is part of a larger coordinated set of papers evaluating multiple domains of flourishing, which included 17 childhood predictors on the intake survey. The variables were selected to be meaningful across multiple flourishing outcomes that make up the Global Flourishing Study (GFS). The final set of 13 childhood predictors was selected after pre-testing due to issues of multicollinearity. The items were selected in collaboration with Gallup, a global analytics and advisory firm, which conducted the sampling. Such a cross-national study is instrumental in exploring variations in cultural contexts. This study asked the following research questions and related hypotheses:

1. What childhood factors are associated with reported health limitations in adulthood? Hypothesis 1: certain childhood predictors (*see below for a list of variables) will show meaningful associations with an individual’s self-reported health limitations in adulthood.
2. To what extent do those factors vary across countries? Hypothesis 2: the strength of associations between the 13 childhood predictors and an individual’s self-reported health limitations in adulthood will vary by country, reflecting the influence of diverse sociocultural, economic, and health contexts that characterize each nation.
3. Are these observed relationships robust to potential confounding as assessed by *E*-values? Hypothesis: the observed associations between the 13 childhood predictors and an individual’s self-reported perceptions of health limitations will be robust against potential unmeasured confounding. *E*-values will suggest some associations would require strong confounding effects to explain away the associations.

## Methods

The description of the methods below has been adapted from the study profile [18]. Further methodological details are available elsewhere [17–25].

### Data

The GFS is a study of 202,898 participants from 22 geographically and culturally diverse countries, with nationally representative sampling within each country, concerning the distribution of determinants of well-being. Wave 1 of the data included the following countries and territories: Argentina, Australia, Brazil, Egypt, Germany, Hong Kong (Special Administrative Region of China), India, Indonesia, Israel, Japan, Kenya, Mexico, Nigeria, the Philippines, Poland, South Africa, Spain, Sweden, Tanzania, Türkiye, United Kingdom, and the USA. The countries were selected to (a) maximize coverage of the world’s population, (b) ensure geographic, cultural, and religious diversity, and (c) prioritize feasibility and existing data collection infrastructure. Data collection was carried out by Gallup Inc. Data for Wave 1 were collected principally during 2023, with some countries beginning data collection in 2022 and exact dates varying by country [23]. Four additional waves of panel data on the participants will be collected annually from 2024 to 2027. The precise sampling design to ensure nationally representative samples varied by country, and further details are available elsewhere [23]. Survey items included aspects of well-being such as happiness, health, meaning, character, relationships, and financial stability [1], along with other demographic, social, economic, political, religious, personality, childhood, community, health, and well-being variables. The data are publicly available through the Center for Open Science (https://www.cos.io/gfs). During the translation process, Gallup adhered to the TRAPD model (translation, review, adjudication, pretesting, and documentation) for cross-cultural survey research (ccsg.isr.umich.edu/chapters/translation/overview) [26].

### Measures

#### Outcome variable

The presence of Health Limitations was assessed with one question: “Do you have any health problems that prevent you from doing any of the things people your age normally can do?” with response options: yes [1] vs no [0].

#### Childhood predictors

The childhood predictor variables included: age (year of birth), gender, marital status/family structure, age 12 religious service attendance, relationship with mother, relationships with father, outsider growing up, abuse, self-rated health growing up, subjective financial status of family growing up, immigration status, race/ethnicity (when available), religious affiliation at age 12.

Relationship with mother during childhood was assessed with the question: “Please think about your relationship with your mother when you were growing up. In general, would you say that relationship was very good, somewhat good, somewhat bad, or very bad?” Responses were dichotomized to very/somewhat good versus very/somewhat bad. An analogous variable was used for relationship with father. “Does not apply” was treated as a dichotomous control variable for respondents who did not have a mother or father due to death or absence. Parental marital status during childhood was assessed with responses of married, divorced, never married, and one or both had died. Financial status was measured with: “Which one of these phrases comes closest to your own feelings about your family’s household income when you were growing up, such as when YOU were around 12 years old?” Responses were lived comfortably, got by, found it difficult, and found it very difficult. Abuse was assessed with yes/no responses to “Were you ever physically or sexually abused when you were growing up?” Participants were separately asked: “When you were growing up, did you feel like an outsider in your family?” Childhood health was assessed by: “In general, how was your health when you were growing up? Was it excellent, very good, good, fair, or poor?” Immigration status was assessed with: “Were you born in this country, or not?” Religious attendance during childhood was assessed with: “How often did YOU attend religious services or worship at a temple, mosque, shrine, church, or other religious building when YOU were around 12 years old?” with responses of at least once/week, one-to-three times/month, less than once/month, or never. Gender was assessed as male, female, or other. Continuous age (year of birth) was classified as 18–24, 25–29, 30–39, 40–49, 50–59, 60–69, 70–79, and 80 or older. Childhood religious tradition/affiliation had response categories of Christianity, Islam, Hinduism, Buddhism, Judaism, Sikhism, Baha’i, Jainism, Shinto, Taoism, Confucianism, Primal/Animist/Folk religion, Spiritism, African-derived, some other religion, or no religion/atheist/agnostic; precise response categories varied by country [25]. Racial/ethnic identity was assessed in some, but not all, countries, and response categories were unique to each country. For additional details on the assessments, see the Center for Open Science GFS codebook and the development of the survey [17, 19].

### Analysis

Descriptive statistics for the observed sample, weighted to be nationally representative within a country, were estimated for each childhood demographic category. Different methods were used to build country samples, resulting in different and customized approaches for weighting the data to be nationally representative [21, 23]. Country weights included only those who completed, both, the intake and annual surveys. A weighted modified Poisson regression model [27] with complex survey adjusted standard errors was fit within each country by regressing health problems in adulthood on all the aforementioned childhood predictor variables simultaneously. In the primary analyses, random effects meta-analyses of the regression coefficients [28, 29] along with confidence intervals, estimate proportions of effects across countries with effect sizes (risk-ratios) larger than 1.1 and smaller than 0.9, and ${I}^{2}$ for evidence concerning variation of effects across countries [30]. Forest plots of estimates are available in the online supplement. Religious affiliation/tradition and race/ethnicity were used within the country as control variables, when available, but these coefficients themselves were not included in the meta-analyses since categories/responses varied by country. Output from the random effects meta-analysis are estimates of the pooled effect of each childhood predictor across countries with standard deviations estimated from a hypothetical population representing the total sample. Country-specific results are also provided in Additional file 1: Tables S1–S22 with coefficients representing calibrated effect sizes adjusting for the other variables in the models. The calibrated effect size and other analysis decisions are discussed further in Padgett, et. al. [22]. Random effects meta-analysis was used to offer a global perspective of each global flourishing outcome across countries. The approach helps to explore heterogeneity across countries represented by the measure Tau. Tau, the estimated standard deviation of effects, provides a measure of variation in the effect across countries included in the GFS. All meta-analyses were conducted in R [31] using the metafor package [32]. Within each country, a global test of association of each childhood predictor variable group with the outcome was conducted, and a pooled *p*-value [33] across countries reported concerning evidence for association within any country. Bonferroni corrected *p*-value thresholds are provided based on the number of childhood demographic variables [34, 35]. For each childhood predictor, we calculated *E*-values to evaluate the sensitivity of results to unmeasured confounding. An *E*-value is the minimum strength of the association an unmeasured confounder must have with both the outcome and the predictor, above and beyond all measured covariates, for an unmeasured confounder to explain away an association [36]. The *E*-value, as used in this study, allows us to evaluate the potential of unmeasured confounding that could result in a null finding. Such an unmeasured confounder would need to be equal to or greater than the *E*-value for both the outcome and the predictor to explain away the observed association. As a supplementary analysis, population-weighted meta-analyses of the regression coefficients were estimated. All analyses were pre-registered with the Center for Open Science prior to data access, with only slight subsequent modification in the regression analyses due to multicollinearity [37]. All code to reproduce analyses is openly available in an online repository [38].

### Missing data

Missing data on all variables was imputed using multivariate imputation by chained equations, and five imputed datasets were used [39, 40]. To account for variation in the assessment of certain variables across countries (e.g., religious affiliation/tradition and race/ethnicity), the imputation process was conducted separately in each country. This within-country imputation approach ensured that the imputation models accurately reflected country-specific contexts and assessment methods. Sampling weights were included in the imputation model to account for specific-variable missingness that may have been related to the probability of inclusion in the study.

### Accounting for complex sampling design

The GFS used different sampling schemes across countries based on the availability of existing panels and recruitment needs [21, 23]. All analyses accounted for the complex survey design components by including weights, primary sampling units, and strata. Additional methodological detail, including accounting for the complex sampling design, is provided elsewhere [22]. STROBE guidelines [41] and checklist (see Additional file 2: STROBE checklist) were used in preparing the manuscript.

## Results

Nationally representative descriptive statistics of the retrospective childhood predictors are shown in Table 1. Most of the sample reported having a very good relationship with their mothers (63%) and fathers (53%), and the majority (75%) of couples were married. Seventy-six percent of the sample reported living comfortably or getting by growing up. Abuse as a child was relatively low among the sample (14% reporting abuse) and the majority of the sample (82%) did not feel like an outsider in their family while growing up. Most individuals (87%) reported self-rated health while growing up as excellent, very good, or good. Regarding religious service attendance at age 12, 41% attended at least 1 per week, and 23% never attended religious services.

**Table 1:** Nationally representative descriptive statistics of the retrospective childhood predictors

**Characteristic**	**N = 202,898**^1^
**Relationship with mother**	
Very good	127,836 (63%)
Somewhat good	52,439 (26%)
Somewhat bad	11,060 (5.5%)
Very bad	4,642 (2.3%)
Does not apply	5,965 (2.9%)
(Missing)	956 (0.5%)
**Relationship with father**	
Very good	107,742 (53%)
Somewhat good	55,714 (27%)
Somewhat bad	15,807 (7.8%)
Very bad	8,278 (4.1%)
Does not apply	13,985 (6.9%)
(Missing)	1,372 (0.7%)
**Parent marital status**	
Parents married	152,001 (75%)
Divorced	17,726 (8.7%)
Parents were never married	15,534 (7.7%)
One or both parents had died	7,794 (3.8%)
(Missing)	9,843 (4.9%)
**Subjective financial status of family growing up**	
Lived comfortably	70,861 (35%)
Got by	82,905 (41%)
Found it difficult	35,852 (18%)
Found it very difficult	12,606 (6.2%)
(Missing)	674 (0.3%)
**Abuse**	
Yes	29,139 (14%)
No	167,279 (82%)
(Missing)	6,479 (3.2%)
**Outsider growing up**	
Yes	28,732 (14%)
No	170,577 (84%)
(Missing)	3,589 (1.8%)
**Self-rated health growing up**	
Excellent	67,121 (33%)
Very good	63,086 (31%)
Good	47,378 (23%)
Fair	19,877 (9.8%)
Poor	4,906 (2.4%)
(Missing)	530 (0.3%)
**Immigration status**	
Born in this country	190,998 (94%)
Born in another country	9,791 (4.8%)
(Missing)	2,110 (1.0%)
**Age 12 religious service attendance**	
At least 1/week	83,237 (41%)
1-3/month	33,308 (16%)
<1/month	36,928 (18%)
Never	47,445 (23%)
(Missing)	1,980 (1.0%)
**Year of birth**	
1998-2005; age 18-24	27,007 (13%)
1993-1998; age 25-29	20,700 (10%)
1983-1993; age 30-39	40,256 (20%)
1973-1983; age 40-49	34,464 (17%)
1963-1973; age 50-59	31,793 (16%)
1953-1963; age 60-69	27,763 (14%)
1943-1953; age 70-79	16,776 (8.3%)
1943 or earlier; age 80+	4,119 (2.0%)
(Missing)	20 (<0.1%)
**Gender**	
Male	98,411 (49%)
Female	103,488 (51%)
Other	602 (0.3%)
(Missing)	397 (0.2%)
**Country**	
Argentina	6,724 (3.3%)
Australia	3,844 (1.9%)
Brazil	13,204 (6.5%)
Egypt	4,729 (2.3%)
Germany	9,506 (4.7%)
Hong Kong	3,012 (1.5%)
India	12,765 (6.3%)
Indonesia	6,992 (3.4%)
Israel	3,669 (1.8%)
Japan	20,543 (10%)
Kenya	11,389 (5.6%)
Mexico	5,776 (2.8%)
Nigeria	6,827 (3.4%)
Philippines	5,292 (2.6%)
Poland	10,389 (5.1%)
South Africa	2,651 (1.3%)
Spain	6,290 (3.1%)
Sweden	15,068 (7.4%)
Tanzania	9,075 (4.5%)
Türkiye	1,473 (0.7%)
United Kingdom	5,368 (2.6%)
United States	38,312 (19%)

^1^n (%); History of abuse was not collected in Israel.

Table 2 presents the random effects meta-analysis of health limitations on retrospective childhood predictors by each demographic variable. The risk of a health limitation was especially higher among those experiencing physical or sexual abuse as a child (RR = 1.59, 95% CI: 1.46, 1.74). Self-rated health growing up was associated with lower risk of reporting health limitations, with risk ratios of 0.75 for excellent health (95% CI 0.68, 0.82) and 0.83 (95% CI 0.78, 0.88) for very good health relative to good health. Conversely, poor self-rated health growing up was associated with increased risk of having a health limitation by 64% (95% CI 1.43, 1.88). Feeling like an outsider growing up was associated with health limitations, with a risk ratio of 1.25 (95% CI 1.16, 1.34). In addition, individuals had a greater health limitation risk if the parent marital status was living in a single, never-married household (RR = 1.15, 95% CI: 1.00, 1.31) or if one or both parents had died (RR = 1.14, 95% CI: 1.03, 1.27) and possibly if divorced (RR = 1.06, 95% CI 1.00, 1.13). A subjective financial status of very difficult growing up had a risk ratio for health limitations of 1.13 (95% CI 1.07, 1.20). As expected, earlier birth cohorts showed much higher risks of health limitations as adults. Immigration status was not especially strongly associated with the risk of reporting a health limitation (RR = 0.93; 95% CI 0.82, 1.04); however, it was associated with changes in risk for health limitations in specific countries described later.

**Table 2:** Random effects meta-analysis of regressing health problems on retrospective childhood predictors

				Estimated Proportion of Effects by Threshold			
Variable	Category	Risk Ratio	95% CI	< 0.90	> 1.10		Global p-value
Relationship with mother	(Ref: Very bad/somewhat bad)						0.686
	Very good/somewhat good	1.02	(0.98,1.06)	0.00	0.00	<0.1ǂ	
Relationship with father	(Ref: Very bad/somewhat bad)						0.487
	Very good/somewhat good	1.01	(0.98,1.05)	0.00	0.00	<0.1ǂ	
Parent marital status	(Ref: Parents married)						<.001**
	Divorced	1.06	(1.00,1.13)	0.00	0.23	35.7	
	Single, never married	1.15	(1.00,1.31)	0.05	0.55	85.6	
	One or both parents had died	1.14	(1.03,1.27)	0.09	0.59	67.9	
Subjective financial status of family growing up	(Ref: Got by)						<.001**
	Lived comfortably	0.96	(0.92,0.99)	0.09	0.00	33.5	
	Found it difficult	1.08	(1.02,1.14)	0.09	0.41	67.4	
	Found it very difficult	1.13	(1.07,1.20)	0.00	0.64	24.1	
Abuse	(Ref: No)						<.001**
	Yes	1.59	(1.46,1.74)	0.00	1.00	89.1	
Outsider growing up	(Ref: No)						<.001**
	Yes	1.25	(1.16,1.34)	0.00	0.86	75.8	
Self-rated health growing up	(Ref: Good)						<.001**
	Excellent	0.75	(0.68,0.82)	0.77	0.05	85.6	
	Very good	0.83	(0.78,0.88)	0.68	0.00	70.2	
	Fair	1.26	(1.17,1.35)	0.00	0.73	73.0	
	Poor	1.64	(1.43,1.88)	0.00	1.00	84.1	
Immigration status	(Ref: Born in this country)						<.001**
	Born in another country	0.93	(0.82,1.04)	0.45	0.18	62.5	
Age 12 religious service attendance	(Ref: Never)						<.001**
	At least 1/week	1.02	(0.96,1.07)	0.05	0.27	36.9	
	1-3/month	1.02	(0.95,1.10)	0.09	0.23	55.5	
	< 1/month	0.98	(0.93,1.03)	0.09	0.05	38.4	
Year of birth	(Ref: 1998-2005; age 18-24)						<.001**
	1993-1998; age 25-29	1.05	(0.97,1.14)	0.14	0.36	41.8	
	1983-1993; age 30-39	1.16	(1.07,1.26)	0.00	0.64	59.6	
	1973-1983; age 40-49	1.56	(1.40,1.73)	0.00	0.95	76.7	
	1963-1973; age 50-59	2.02	(1.72,2.36)	0.00	0.95	89.3	
	1953-1963; age 60-69	2.63	(2.17,3.18)	0.00	1.00	92.0	
	1943-1953; age 70-79	3.18	(2.56,3.95)	0.00	1.00	92.0	
	1943 or earlier; age 80+^ǂ^	1.69	(0.41,7.05)	0.09	0.91	99.6	
Gender	(Ref: Male)						<.001**
	Female	1.12	(1.05,1.20)	0.05	0.50	88.0	
	Other^ǂ^	0.11	(0.01,1.01)	0.39	0.50	99.5	

*Note.* *N*= 202,898.*p < .05; **p < .004 (Bonferroni corrected threshold); ^ǂ^Group is very small (<0.1% of the observed sample) within several countries leading large uncertainty in this estimate or even complete separation—be cautious about interpreting this estimate; CI = confidence interval; the estimated proportion of effects is the estimated proportion of effects above (or below) a threshold based on the calibrated effect sizes^30^; *I*^2^is an estimate of the variability in means due to heterogeneity across countries vs. sampling variability; the Global *p*-value corresponds to the joint test of the null hypothesis that the country-specific joint parameter Wald tests (all parameters within variable groups are zero) are all null all 22 countries; and additional details of heterogeneity of effects are available in the forest plots of Additional file 1: Figures S1-S26.

Sensitivity of meta-analyzed childhood predictors to unmeasured confounding is shown in Table 3 with reported *E*-values. *E*-values report how much an unmeasured confounder would have to be related to the childhood predictor and health problems to explain away an effect estimate. The minimum *E*-value is 1 (no unmeasured confounding is needed to explain away the observed association); the higher the *E*-value, the stronger the unmeasured confounder associations would have to be to explain away the effect. For example, to explain away the association of physical or sexual abuse as a child and adult health limitations, an unmeasured confounder that is associated with both childhood abuse and adult health limitations by risk ratios of 2.57 each, above and beyond the measured covariates, could suffice, but weaker joint confounder associations could not. Associations for poor self-rated health were likewise especially robust. To shift the 95% confidence interval to include the null, an unmeasured confounder associated with both childhood abuse and adult health limitations by risk ratios of 2.27 each, above and beyond the measured covariates, could suffice, but weaker joint confounder associations could not. Other predictors had somewhat smaller *E*-values, such as religious attendance and relationships with mother and father, suggesting that a weak confounding variable could potentially explain away the association.

**Table 3:** Sensitivity of meta-analyzed retrospective childhood predictors to unmeasured confounding

Variable	Category	E-value for Estimate	E-value for 95% CI
Relationship with mother	(Ref: Very bad/somewhat bad)		
	Very good/somewhat good	1.16	1.00
Relationship with father	(Ref: Very bad/somewhat bad)		
	Very good/somewhat good	1.14	1.00
Parent marital status	(Ref: Parents married)		
	Divorced	1.33	1.05
	Single, never married	1.56	1.07
	One or both parents had died	1.54	1.20
Subjective financial status of family growing up	(Ref: Got by)		
	Lived comfortably	1.27	1.10
	Found it difficult	1.37	1.15
	Found it very difficult	1.52	1.35
Abuse	(Ref: No)		
	Yes	2.57	2.27
Outsider growing up	(Ref: No)		
	Yes	1.80	1.60
Self-rated health growing up	(Ref: Good)		
	Excellent	2.00	1.72
	Very good	1.70	1.52
	Fair	1.83	1.61
	Poor	2.65	2.20
Immigration status	(Ref: Born in this country)		
	Born in another country	1.37	1.00
Age 12 religious service attendance	(Ref: Never)		
	At least 1/week	1.14	1.00
	1-3/month	1.17	1.00
	< 1/month	1.17	1.00
Year of birth	(Ref: 1998-2005; age 18-24)		
	1993-1998; age 25-29	1.29	1.00
	1983-1993; age 30-39	1.59	1.34
	1973-1983; age 40-49	2.49	2.15
	1963-1973; age 50-59	3.45	2.83
	1953-1963; age 60-69	4.69	3.76
	1943-1953; age 70-79	5.81	4.56
	1943 or earlier; age 80+^ǂ^	2.77	1.00
Gender	(Ref: Male)		
	Female	1.49	1.27
	Other^ǂ^	17.32	1.00

Note. N = 202,898; the E-value is the minimum strength of the association an unmeasured confounder must have with both the outcome (health problems) and the predictor, above and beyond all measured covariates, for an unmeasured confounder to explain away an association^36^; and ^ǂ^Group is very small (<0.1% of the observed sample) within several countries potentially leading to complete separation and large uncertainty in this estimate—be cautious about interpreting this estimate.

Country-specific descriptive statistics, effect size estimates, and sensitivity analyses are found in Additional file 1: Tables S1–S22. Additional file 1: Table S23 shows the population weighted meta-analysis of regression results and sensitivity estimates.

## Discussion

Using data from 22 countries, we investigated childhood predictors of health limitations, defined as health problems that prevent individuals from doing things that people their age normally can do. As shown in Table 1, the majority of our sample had good relationships with their parents, were raised in households where their biological parents were married, and did not experience severe financial hardships or abuse during their childhood.

One of the most notable findings was the robust and consistent influence of childhood experience of physical or sexual abuse on health limitations. Experiencing childhood abuse increased the risk of health limitations by 1.46–1.74 times compared to those who never experienced abuse. The effect size for abuse was comparable to that of having poor health growing up, making these two the strongest predictors of health problems among *modifiable* determinants of health. As shown in the forest plots of Additional file 1: Figs. S1–S26, the effect of abuse was consistently associated across all countries (note: the question was not asked in Israel) except for Turkey. In contrast, self-rated health was not quite as consistent. Healthy aging requires an understanding of upstream determinants influencing children from conception to adulthood. Abuse and self-rated health are two such predictors that increase additional risk factors such as risky health behaviors, educational attainment, and early onset of chronic diseases [42].

Adverse childhood experiences (ACEs) are known to have detrimental effects on child development, mentally and physically [43]. A recent framework on ACEs, based on neurocognitive development of youth, categorizes ACEs into threat, deprivation, and unpredictability [44]. Studies have found that distinct types of ACEs have differential effects on developmental outcomes [45]. Although the current study did not directly assess different types of ACEs, experiences of physical or sexual abuse align with the concept of threat, while growing up feeling like an outsider and facing financial hardships resemble forms of deprivation. Our findings indicate that threat-related ACEs, rather than deprivation-related ACEs, might have more substantial effects on health problems associated with functional limitations. Deprivation-related childhood experiences were significant predictors of such health limitations in some countries (e.g., Australia, Brazil, Germany, Hong Kong, Japan, Indonesia, Israel, Mexico, Spain, and Sweden), while threat-based experiences predicted health limitations in all countries but Turkey. Further investigation on cross-cultural differences in the association between specific types of ACEs and health limitations will inform the development of tailored interventions for different cultures.

Parents’ marital status growing up was also an important predictor of health limitations overall and especially so in some particular countries (e.g., Indonesia, Spain). Both difficult parenting experiences and social stigma around single-parent or divorced families in certain cultures could increase children’s stress through neglect or unpredictable situations, which could stunt physical and cognitive development, thereby increasing their risk for health problems [46]. It is also possible that in some countries, non-traditional families experience financial hardships that limit their access to health care, education, or other basic needs, increasing the risk of health limitations. In this study, financial hardship also increased the risk of health limitations. Childhood socioeconomic status (SES) has been shown to be associated with subsequent prevalence rates of dementia in adulthood [47]. Neurological disorders are among the top causes of the global burden of disease [48] and a primary health problem preventing age-appropriate functioning. Among high-income countries, dementia will be the leading cause of mortality [49].

Interestingly, the impact of immigration status was more complex. It was associated with a *lower* risk for health limitations in Spain, Germany, the United Kingdom, and the USA. In contrast, immigration status in Nigeria and Indonesia was associated with a higher risk for health limitations [50]. Immigrants may have different access to support in some countries compared to others, or different social norms for “what people their age normally do” compared to citizens. Among Mexican immigrants to the USA, men and women experience different trajectories related to limitations in activities of daily living. The later one migrates, the more likely they are to experience health-related limitations, suggesting a multidimensional aspect[51]. It may be that the health behaviors immigrants brought from their previous countries affected their health behaviors and were associated with health limitations.

The relationship between religiosity (religious affiliation and service attendance) and health limitations requires consideration of the type of religious beliefs. Some countries with high levels of religious service attendance, as seen in Nigeria, Kenya, and Indonesia, could be related to such beliefs or based on social compulsion compared to voluntary attendance. The literature generally strongly supports a salutary mechanism between religiosity and health. However, negative relationships are also possible [52].

These data support the first two hypotheses by showing (a) varying levels of childhood predictors across countries, (b) variation in risk associated with the childhood predictors across countries. There was notable evidence for birth year influencing health limitations across all countries, and likewise for childhood abuse (global RR = 1.59) in all countries except Turkey, and for self-rated health in all except Nigeria and South Africa. Abuse as a child was not asked by Gallup in Israel. One interesting area of variation across countries was that religious affiliation growing up was notably associated with adult health limitations in five countries, with four of those being in Asia: Hong Kong, Indonesia, Japan, and the Philippines. Religious affiliation is noted to be associated with health-promoting behaviors in some countries, and some religions, such as Islam and Buddhism, may stigmatize some health conditions, such as mental health, associated with a lack of faith [53]. Failure to access health care for mental health may lead to physical limitations later in life. Meditation practices associated with such religious practices may also improve mental health conditions and should be an area for further investigation. Country variation was also observed for parental marital status, financial status, and feeling like an outsider growing up. Growing up in a connected family is associated with greater financial status and increased access to services, reducing the risk of future physical limitations [54]. However, in some cultures like Japan, self-esteem and other psychosocial stressors could also mediate this relationship, resulting in a higher risk of limitations in late adulthood [55].

This study suggests country-level variation exists regarding predictors of health limitations, highlighting the importance of understanding the differences in social, physical, and spiritual factors during childhood that influence health limitations as adults and overall, human flourishing. Among the measured modifiable childhood predictors, abuse, poor self-rated health, and growing up an outsider were consistently salient across countries. Hypothesis three was moderately supported in that several predictors were robust to the influence of unmeasured confounding as measured by the *E*-values. The *E*-value estimates for higher age groups of 60 + (*E*-value = 5.81) and childhood abuse (2.57) showed moderate robustness to unmeasured confounding, leading to greater confidence in the association with health limitations. E-value estimates among categories with small group sizes should be interpreted with caution, as small group sizes could lead to complete separation and large uncertainty. The results highlight the importance of longitudinal studies investigating the relationship between childhood abuse and health limitations to identify potential protective and moderating factors that might reduce the effect of abuse later in life.

The strengths of this study include the diverse and global representation of countries involved, the large sample size as part of the GFS, nationally representative sampling, and an extensive list of childhood predictors to explore cross-nationally. The study has several limitations, including recall bias, given that individuals were asked to recall childhood circumstances. However, for recall bias to completely explain away the observed associations would require that the effect of adult health limitations on biasing retrospective assessments of the childhood predictors would essentially have to be at least as strong as the observed associations themselves, and some of these were quite substantial [56]. The outcome of health limitations was a self-reported measure and may be interpreted differently between individuals and cultures. We acknowledge that relying on self-reported measures without objective assessments of health conditions may be a limitation. However, formal diagnoses can be influenced by factors like access to healthcare, income, and trust in medical professionals, making self-reported data a valuable complement in understanding health-related quality of life. The cross-sectional nature of the data may limit our ability to draw causal interpretations from our findings, and some evidence suggests retrospective assessment of adverse childhood experiences may lead to an overestimation of their impact on self-reported outcomes like health limitations [57]. Unmeasured confounding could influence some of the results; however, the *E*-values support robustness for some of the predictors. The study included countries that represent the majority of the global population. However, generalizability to countries not included in the study should be done with caution. Additional sources of bias include institutionalized individuals residing in assisted living, long-term care, or nursing home facilities, who have a higher likelihood of health limitations, and were likely under-represented in the sampling. In addition, functional and cognitive limitations are strong predictors of survey participation [58]. This differential non-response bias could also underestimate the reported effect sizes. Lastly, the GFS uses single-item questions of core indicators of human flourishing. The items were selected from established scales; however, using single items is a limitation of the study.

## Conclusions

This study underscores the cross-cultural variation in childhood predictors of health problems that impede typical age-related activities. The findings indicate that the most impactful modifiable predictors are experiences of physical or sexual abuse during childhood, self-rated health in childhood, and the feeling of growing up as an outsider. Protecting children from trauma is critical in improving health-related quality of life across the life course. These insights highlight the critical need for targeted interventions and policies that address these factors to mitigate long-term health limitations. Future research should explore the mechanisms through which these predictors influence health outcomes across different cultural contexts, social stigma, and system-level factors that increase risk through childhood. Efforts should focus on developing and evaluating culturally sensitive prevention and intervention strategies. By deepening our understanding of these predictors and their cultural nuances, we can better inform public health initiatives aimed at improving childhood health, increasing quality of life in adulthood, and reducing disparities in health outcomes.

## Supplementary Information

Additional file 1: GFS Childhood Predictors of Health Problems in Adulthood Country Level Analyses

Additional file 2:STROBE checklist

## Data Availability

All analyses were pre-registered through the Center for Open Science Open Science Framework prior to data access (https://osf.io/8qs6v) [37]. The analysis code used to conduct the analysis is openly available in an online repository through the Center for Open Science (https://osf.io/vbype) [38]. The data used in this study are available through the Center for Open Science (https://www.cos.io/gfs). The dataset used was Wave 1 non-sensitive Global data available February 2024 - March 2026 via preregistration and publicly from then onwards [24].

## Abbreviations

ACE: Adverse Childhood Experiences
COS: Center for Open Science
GFS: Global Flourishing Study
PCE: Positive Childhood Experiences
SES: Socioeconomic Status
STROBE: Strengthening the Reporting of Observational Studies in Epidemiology
TRAPD: Translation, Review, Adjudication, Pretesting, and Documentation
