# Supplementary material for: Childhood predictors of health limitations in life across 22 countries: a cross-national and cross-sectional analysis
Source: BMC Glob Public Health. 2025 Aug 13;3:70. doi: 10.1186/s44263-025-00188-0 (PMC12351978; doi:10.1186/s44263-025-00188-0)

**GFS Online Supplement**

Important Notes and Caveats

This online supplement to the Global Flourishing Study paper reporting childhood predictors of self-reported health problems later in life has several important caveats to interpretation. First, this study focused on childhood predictors using a retrospective (recall) approach to obtain information about the respondents’ lives at age 12. Several the childhood characteristics such as relationship quality with parents, subjective financial status growing up, etc. are necessarily highly related. This led to multicollinearity issues so some effects may be unstable. Secondly, the low degree of variability in the outcome in some countries for some subgroups leads to the issues of complete separation in the modified Poisson regression model. The complete separation results in uninterpretable estimates for those effects as there were no observed differences between the exposure and the outcome in this sample. We have tried to note when this occurs in the meta-analyses. Third, in rare instances for childhood predictor analyses the confidence interval of the effect estimate can contradict the reported global p-value (e.g., for the single-category effects of relationship with mother). In such cases, the reported confidence interval is more robust with corrected degrees of freedom from the pooling across multiple imputations, whereas the global p-value is based on a Wald-type test and is less robust to uncertainty attributable to multiple imputation. Lastly, comparing results across countries should be done with caution due to possible measurement non-invariance, differences in translation, differences in the quality of weights used to project the sample to be nationally representative, and unmeasured confounders that may specific to each country.

Table S1a. Nationally representative descriptive statistics for Argentina

| **Characteristic** | **N = 6,724**^1^ |
| --- | --- |
| **Relationship with mother** |  |
| Very good | 4,463 (66%) |
| Somewhat good | 1,436 (21%) |
| Somewhat bad | 299 (4.4%) |
| Very bad | 216 (3.2%) |
| Does not apply | 273 (4.1%) |
| (Missing) | 36 (0.5%) |
| **Relationship with father** |  |
| Very good | 3,612 (54%) |
| Somewhat good | 1,537 (23%) |
| Somewhat bad | 440 (6.5%) |
| Very bad | 401 (6.0%) |
| Does not apply | 694 (10%) |
| (Missing) | 39 (0.6%) |
| **Parent marital status** |  |
| Parents married | 4,110 (61%) |
| Divorced | 637 (9.5%) |
| Parents were never married | 1,368 (20%) |
| One or both parents had died | 199 (3.0%) |
| (Missing) | 410 (6.1%) |
| **Subjective financial status of family growing up** |  |
| Lived comfortably | 2,042 (30%) |
| Got by | 2,305 (34%) |
| Found it difficult | 1,789 (27%) |
| Found it very difficult | 569 (8.5%) |
| (Missing) | 19 (0.3%) |
| **Abuse** |  |
| Yes | 1,302 (19%) |
| No | 5,271 (78%) |
| (Missing) | 151 (2.2%) |
| **Outsider growing up** |  |
| Yes | 1,165 (17%) |
| No | 5,458 (81%) |
| (Missing) | 101 (1.5%) |
| **Self-rated health growing up** |  |
| Excellent | 2,402 (36%) |
| Very good | 1,819 (27%) |
| Good | 1,830 (27%) |
| Fair | 505 (7.5%) |
| Poor | 156 (2.3%) |
| (Missing) | 12 (0.2%) |
| **Immigration status** |  |
| Born in this country | 6,346 (94%) |
| Born in another country | 348 (5.2%) |
| (Missing) | 29 (0.4%) |
| **Age 12 religious service attendance** |  |
| At least 1/week | 2,601 (39%) |
| 1-3/month | 1,204 (18%) |
| <1/month | 1,059 (16%) |
| Never | 1,808 (27%) |
| (Missing) | 53 (0.8%) |
| **Year of birth** |  |
| 1998-2005; age 18-24 | 1,108 (16%) |
| 1993-1998; age 25-29 | 719 (11%) |
| 1983-1993; age 30-39 | 1,432 (21%) |
| 1973-1983; age 40-49 | 1,254 (19%) |
| 1963-1973; age 50-59 | 1,014 (15%) |
| 1953-1963; age 60-69 | 730 (11%) |
| 1943-1953; age 70-79 | 356 (5.3%) |
| 1943 or earlier; age 80+ | 112 (1.7%) |
| (Missing) | 0 (0%) |
| **Gender** |  |
| Male | 3,143 (47%) |
| Female | 3,542 (53%) |
| Other | 21 (0.3%) |
| (Missing) | 18 (0.3%) |
| **Religious affiliation** |  |
| Christianity | 5,805 (86%) |
| Islam | 11 (0.2%) |
| Hinduism | 2 (<0.1%) |
| Buddhism | 3 (<0.1%) |
| Judaism | 51 (0.8%) |
| Sikhism | 5 (<0.1%) |
| Baha'i | 0 (0%) |
| Jainism | 0 (0%) |
| Shinto | 0 (0%) |
| Taoism | 1 (<0.1%) |
| Confucianism | 0 (0%) |
| Primal, Animist, or Folk religion | 17 (0.2%) |
| Spiritism | 0 (0%) |
| Umbanda, Candomble, and other African-derived religions | 0 (0%) |
| Chinese folk/traditional religion | 0 (0%) |
| Some other religion | 10 (0.2%) |
| No religion/Atheist/Agnostic | 697 (10%) |
| (Missing) | 122 (1.8%) |
| **Race/Ethnicity** |  |
| Asian | 43 (0.6%) |
| Black | 95 (1.4%) |
| Indigenous | 129 (1.9%) |
| Mestizo(a) | 1,801 (27%) |
| Mullato(a) | 75 (1.1%) |
| Other | 104 (1.5%) |
| White | 3,406 (51%) |
| (Missing) | 1,070 (16%) |
| ^1^n (%) | |

Table S1b. Childhood predictors regression for Argentina

| Variable | Category | Risk-Ratio | RR 95% CI | Global p-value |
| --- | --- | --- | --- | --- |
| Relationship with mother | (Ref: Very bad/somewhat bad) |  |  | 0.335 |
|  | Very good/somewhat good | 0.89 | (0.71,1.13) |  |
| Relationship with father | (Ref: Very bad/somewhat bad) |  |  | 0.533 |
|  | Very good/somewhat good | 0.94 | (0.77,1.16) |  |
| Parent marital status | (Ref: Parents married) |  |  | 0.598 |
|  | Divorced | 0.95 | (0.73,1.23) |  |
|  | Parents were never married | 0.97 | (0.79,1.20) |  |
|  | One or both parents had died | 1.19 | (0.82,1.74) |  |
| Subjective financial status of family growing up | (Ref: Got by) |  |  | 0.047 |
|  | Lived comfortably | 1.07 | (0.88,1.30) |  |
|  | Found it difficult | 1.29 | (1.07,1.55) |  |
|  | Found it very difficult | 1.22 | (0.95,1.58) |  |
| Abuse | (Ref: No) |  |  | <.001 |
|  | Yes | 1.40 | (1.18,1.67) |  |
| Outsider growing up | (Ref: No) |  |  | 0.141 |
|  | Yes | 1.15 | (0.95,1.40) |  |
| Self-rated health growing up | (Ref: Good) |  |  | <.001 |
|  | Excellent | 0.65 | (0.53,0.79) |  |
|  | Very good | 0.77 | (0.63,0.93) |  |
|  | Fair | 1.35 | (1.08,1.68) |  |
|  | Poor | 1.70 | (1.25,2.32) |  |
| Immigration status | (Ref: Born in this country) |  |  | 0.298 |
|  | Born in another country | 0.84 | (0.61,1.17) |  |
| Age 12 religious service attendance | (Ref: Never) |  |  | 0.477 |
|  | At least 1/week | 0.95 | (0.79,1.15) |  |
|  | 1-3/month | 0.87 | (0.69,1.09) |  |
|  | < 1/month | 0.86 | (0.67,1.10) |  |
| Year of birth | (Ref: 1998-2005; current age: 18-24) |  |  | <.001 |
|  | 1993-1998; age 25-29 | 0.95 | (0.66,1.36) |  |
|  | 1983-1993; age 30-39 | 0.93 | (0.70,1.25) |  |
|  | 1973-1983; age 40-49 | 1.19 | (0.89,1.59) |  |
|  | 1963-1973; age 50-59 | 1.74 | (1.32,2.28) |  |
|  | 1953-1963; age 60-69 | 2.17 | (1.63,2.90) |  |
|  | 1943-1953; age 70-79 | 2.61 | (1.90,3.57) |  |
|  | 1943 or earlier; age 80+ | 3.43 | (2.25,5.24) |  |
| Gender | (Ref: Male) |  |  | <.001 |
|  | Female | 1.06 | (0.90,1.23) |  |
|  | Other | 3.06 | (1.71,5.48) |  |
| Religious affiliation | (Ref: No religion/Atheist/Agnostic) |  |  | 0.793 |
|  | Christianity | 1.00 | (0.76,1.31) |  |
|  | Collapsed affiliations with prevalence<3% | 1.19 | (0.64,2.24) |  |
| Race/ethnicity | (Ref: Plurality group) |  |  | 0.659 |
|  | Non-plurality groups | 0.98 | (0.83,1.16) |  |

Table S1c. Sensitivity to unmeasured confounding of childhood predictors in Argentina

| Variable | Category | E-value for Estimate | E-value for 95% CI |
| --- | --- | --- | --- |
| Relationship with mother | (Ref: Very bad/somewhat bad) |  |  |
|  | Very good/somewhat good | 1.48 | 1.00 |
| Relationship with father | (Ref: Very bad/somewhat bad) |  |  |
|  | Very good/somewhat good | 1.32 | 1.00 |
| Parent marital status | (Ref: Parents married) |  |  |
|  | Divorced | 1.30 | 1.00 |
|  | Parents were never married | 1.20 | 1.00 |
|  | One or both parents had died | 1.67 | 1.00 |
| Subjective financial status of family growing up | (Ref: Got by) |  |  |
|  | Lived comfortably | 1.34 | 1.00 |
|  | Found it difficult | 1.89 | 1.34 |
|  | Found it very difficult | 1.75 | 1.00 |
| Abuse | (Ref: No) |  |  |
|  | Yes | 2.16 | 1.64 |
| Outsider growing up | (Ref: No) |  |  |
|  | Yes | 1.58 | 1.00 |
| Self-rated health growing up | (Ref: Good) |  |  |
|  | Excellent | 2.47 | 1.86 |
|  | Very good | 1.94 | 1.36 |
|  | Fair | 2.03 | 1.37 |
|  | Poor | 2.79 | 1.81 |
| Immigration status | (Ref: Born in this country) |  |  |
|  | Born in another country | 1.65 | 1.00 |
| Age 12 religious service attendance | (Ref: Never) |  |  |
|  | At least 1/week | 1.27 | 1.00 |
|  | 1-3/month | 1.58 | 1.00 |
|  | < 1/month | 1.60 | 1.00 |
| Year of birth | (Ref: 1998-2005; current age: 18-24) |  |  |
|  | 1993-1998; age 25-29 | 1.29 | 1.00 |
|  | 1983-1993; age 30-39 | 1.34 | 1.00 |
|  | 1973-1983; age 40-49 | 1.67 | 1.00 |
|  | 1963-1973; age 50-59 | 2.87 | 1.98 |
|  | 1953-1963; age 60-69 | 3.77 | 2.64 |
|  | 1943-1953; age 70-79 | 4.65 | 3.21 |
|  | 1943 or earlier; age 80+ | 6.33 | 3.93 |
| Gender | (Ref: Male) |  |  |
|  | Female | 1.30 | 1.00 |
|  | Other | 5.58 | 2.82 |
| Religious affiliation | (Ref: No religion/Atheist/Agnostic) |  |  |
|  | Christianity | 1.02 | 1.00 |
|  | Collapsed affiliations with prevalence<3% | 1.67 | 1.00 |
| Race/ethnicity | (Ref: Plurality group) |  |  |
|  | Non-plurality groups | 1.15 | 1.00 |

Table S2a. Nationally representative descriptive statistics for Australia

| **Characteristic** | **N = 3,844**^1^ |
| --- | --- |
| **Relationship with mother** |  |
| Very good | 2,554 (66%) |
| Somewhat good | 925 (24%) |
| Somewhat bad | 218 (5.7%) |
| Very bad | 107 (2.8%) |
| Does not apply | 32 (0.8%) |
| (Missing) | 7 (0.2%) |
| **Relationship with father** |  |
| Very good | 2,032 (53%) |
| Somewhat good | 1,144 (30%) |
| Somewhat bad | 315 (8.2%) |
| Very bad | 196 (5.1%) |
| Does not apply | 148 (3.9%) |
| (Missing) | 9 (0.2%) |
| **Parent marital status** |  |
| Parents married | 3,048 (79%) |
| Divorced | 462 (12%) |
| Parents were never married | 187 (4.9%) |
| One or both parents had died | 96 (2.5%) |
| (Missing) | 52 (1.4%) |
| **Subjective financial status of family growing up** |  |
| Lived comfortably | 1,756 (46%) |
| Got by | 1,496 (39%) |
| Found it difficult | 422 (11%) |
| Found it very difficult | 154 (4.0%) |
| (Missing) | 16 (0.4%) |
| **Abuse** |  |
| Yes | 995 (26%) |
| No | 2,790 (73%) |
| (Missing) | 59 (1.5%) |
| **Outsider growing up** |  |
| Yes | 756 (20%) |
| No | 3,062 (80%) |
| (Missing) | 26 (0.7%) |
| **Self-rated health growing up** |  |
| Excellent | 1,736 (45%) |
| Very good | 1,087 (28%) |
| Good | 603 (16%) |
| Fair | 308 (8.0%) |
| Poor | 106 (2.8%) |
| (Missing) | 4 (<0.1%) |
| **Immigration status** |  |
| Born in this country | 2,953 (77%) |
| Born in another country | 885 (23%) |
| (Missing) | 6 (0.2%) |
| **Age 12 religious service attendance** |  |
| At least 1/week | 1,362 (35%) |
| 1-3/month | 486 (13%) |
| <1/month | 600 (16%) |
| Never | 1,307 (34%) |
| (Missing) | 90 (2.3%) |
| **Year of birth** |  |
| 1998-2005; age 18-24 | 345 (9.0%) |
| 1993-1998; age 25-29 | 282 (7.3%) |
| 1983-1993; age 30-39 | 641 (17%) |
| 1973-1983; age 40-49 | 618 (16%) |
| 1963-1973; age 50-59 | 691 (18%) |
| 1953-1963; age 60-69 | 589 (15%) |
| 1943-1953; age 70-79 | 498 (13%) |
| 1943 or earlier; age 80+ | 178 (4.6%) |
| (Missing) | 2 (<0.1%) |
| **Gender** |  |
| Male | 1,861 (48%) |
| Female | 1,941 (50%) |
| Other | 36 (0.9%) |
| (Missing) | 6 (0.2%) |
| **Religious affiliation** |  |
| Christianity | 2,678 (70%) |
| Islam | 48 (1.2%) |
| Hinduism | 39 (1.0%) |
| Buddhism | 16 (0.4%) |
| Judaism | 29 (0.8%) |
| Sikhism | 6 (0.2%) |
| Baha'i | 5 (0.1%) |
| Jainism | 0 (0%) |
| Shinto | 0 (0%) |
| Taoism | 1 (<0.1%) |
| Confucianism | 0 (0%) |
| Primal, Animist, or Folk religion | 4 (<0.1%) |
| Spiritism | 0 (0%) |
| Umbanda, Candomble, and other African-derived religions | 0 (0%) |
| Chinese folk/traditional religion | 0 (0%) |
| Some other religion | 8 (0.2%) |
| No religion/Atheist/Agnostic | 990 (26%) |
| (Missing) | 21 (0.5%) |
| **Race/Ethnicity** |  |
| Aboriginal | 53 (1.4%) |
| Australian | 1,946 (51%) |
| Australian British/European | 1,047 (27%) |
| Chinese | 75 (1.9%) |
| Indian | 58 (1.5%) |
| Japanese | 1 (<0.1%) |
| Malay | 11 (0.3%) |
| New Zealander | 91 (2.4%) |
| Other | 163 (4.2%) |
| Other European | 357 (9.3%) |
| Russian | 7 (0.2%) |
| Samoan | 4 (0.1%) |
| Sinhalese | 1 (<0.1%) |
| Spanish | 2 (<0.1%) |
| Sri Lankan Moor | 1 (<0.1%) |
| Sri Lankan Tamil | 7 (0.2%) |
| Vietnamese | 7 (0.2%) |
| (Missing) | 14 (0.4%) |
| ^1^n (%) | |

Table S2b. Childhood predictors regression for Australia

| Variable | Category | Risk-Ratio | RR 95% CI | Global p-value |
| --- | --- | --- | --- | --- |
| Relationship with mother | (Ref: Very bad/somewhat bad) |  |  | 0.415 |
|  | Very good/somewhat good | 1.09 | (0.88,1.34) |  |
| Relationship with father | (Ref: Very bad/somewhat bad) |  |  | 0.515 |
|  | Very good/somewhat good | 1.06 | (0.88,1.28) |  |
| Parent marital status | (Ref: Parents married) |  |  | 0.401 |
|  | Divorced | 1.00 | (0.80,1.24) |  |
|  | Parents were never married | 0.86 | (0.59,1.26) |  |
|  | One or both parents had died | 0.71 | (0.46,1.10) |  |
| Subjective financial status of family growing up | (Ref: Got by) |  |  | 0.223 |
|  | Lived comfortably | 0.87 | (0.76,1.00) |  |
|  | Found it difficult | 1.01 | (0.85,1.21) |  |
|  | Found it very difficult | 0.98 | (0.74,1.30) |  |
| Abuse | (Ref: No) |  |  | <.001 |
|  | Yes | 1.40 | (1.22,1.61) |  |
| Outsider growing up | (Ref: No) |  |  | 0.004 |
|  | Yes | 1.26 | (1.07,1.49) |  |
| Self-rated health growing up | (Ref: Good) |  |  | <.001 |
|  | Excellent | 0.62 | (0.52,0.74) |  |
|  | Very good | 0.77 | (0.64,0.92) |  |
|  | Fair | 1.45 | (1.18,1.78) |  |
|  | Poor | 1.79 | (1.39,2.30) |  |
| Immigration status | (Ref: Born in this country) |  |  | 0.072 |
|  | Born in another country | 0.86 | (0.72,1.02) |  |
| Age 12 religious service attendance | (Ref: Never) |  |  | 0.088 |
|  | At least 1/week | 0.99 | (0.84,1.17) |  |
|  | 1-3/month | 0.81 | (0.64,1.03) |  |
|  | < 1/month | 0.82 | (0.65,1.04) |  |
| Year of birth | (Ref: 1998-2005; current age: 18-24) |  |  | <.001 |
|  | 1993-1998; age 25-29 | 1.28 | (0.80,2.05) |  |
|  | 1983-1993; age 30-39 | 1.27 | (0.85,1.89) |  |
|  | 1973-1983; age 40-49 | 1.55 | (1.06,2.26) |  |
|  | 1963-1973; age 50-59 | 2.15 | (1.51,3.08) |  |
|  | 1953-1963; age 60-69 | 2.22 | (1.55,3.19) |  |
|  | 1943-1953; age 70-79 | 2.81 | (1.95,4.05) |  |
|  | 1943 or earlier; age 80+ | 2.75 | (1.83,4.12) |  |
| Gender | (Ref: Male) |  |  | <.001 |
|  | Female | 1.22 | (1.07,1.39) |  |
|  | Other | 1.94 | (1.32,2.84) |  |
| Religious affiliation | (Ref: No religion/Atheist/Agnostic) |  |  | 0.462 |
|  | Christianity | 0.93 | (0.78,1.12) |  |
|  | Collapsed affiliations with prevalence<3% | 0.73 | (0.43,1.25) |  |
| Race/ethnicity | (Ref: Plurality group) |  |  | 0.810 |
|  | Non-plurality groups | 1.01 | (0.88,1.16) |  |

Table S2c. Sensitivity to unmeasured confounding of childhood predictors in Australia

| Variable | Category | E-value for Estimate | E-value for 95% CI |
| --- | --- | --- | --- |
| Relationship with mother | (Ref: Very bad/somewhat bad) |  |  |
|  | Very good/somewhat good | 1.40 | 1.00 |
| Relationship with father | (Ref: Very bad/somewhat bad) |  |  |
|  | Very good/somewhat good | 1.31 | 1.00 |
| Parent marital status | (Ref: Parents married) |  |  |
|  | Divorced | 1.07 | 1.00 |
|  | Parents were never married | 1.58 | 1.00 |
|  | One or both parents had died | 2.16 | 1.00 |
| Subjective financial status of family growing up | (Ref: Got by) |  |  |
|  | Lived comfortably | 1.56 | 1.00 |
|  | Found it difficult | 1.13 | 1.00 |
|  | Found it very difficult | 1.17 | 1.00 |
| Abuse | (Ref: No) |  |  |
|  | Yes | 2.16 | 1.74 |
| Outsider growing up | (Ref: No) |  |  |
|  | Yes | 1.84 | 1.36 |
| Self-rated health growing up | (Ref: Good) |  |  |
|  | Excellent | 2.61 | 2.04 |
|  | Very good | 1.93 | 1.38 |
|  | Fair | 2.25 | 1.63 |
|  | Poor | 2.97 | 2.12 |
| Immigration status | (Ref: Born in this country) |  |  |
|  | Born in another country | 1.61 | 1.00 |
| Age 12 religious service attendance | (Ref: Never) |  |  |
|  | At least 1/week | 1.12 | 1.00 |
|  | 1-3/month | 1.78 | 1.00 |
|  | < 1/month | 1.74 | 1.00 |
| Year of birth | (Ref: 1998-2005; current age: 18-24) |  |  |
|  | 1993-1998; age 25-29 | 1.88 | 1.00 |
|  | 1983-1993; age 30-39 | 1.86 | 1.00 |
|  | 1973-1983; age 40-49 | 2.47 | 1.31 |
|  | 1963-1973; age 50-59 | 3.73 | 2.38 |
|  | 1953-1963; age 60-69 | 3.87 | 2.48 |
|  | 1943-1953; age 70-79 | 5.07 | 3.31 |
|  | 1943 or earlier; age 80+ | 4.93 | 3.06 |
| Gender | (Ref: Male) |  |  |
|  | Female | 1.73 | 1.35 |
|  | Other | 3.29 | 1.98 |
| Religious affiliation | (Ref: No religion/Atheist/Agnostic) |  |  |
|  | Christianity | 1.36 | 1.00 |
|  | Collapsed affiliations with prevalence<3% | 2.07 | 1.00 |
| Race/ethnicity | (Ref: Plurality group) |  |  |
|  | Non-plurality groups | 1.11 | 1.00 |

Table S3a. Nationally representative descriptive statistics for Brazil

| **Characteristic** | **N = 13,204**^1^ |
| --- | --- |
| **Relationship with mother** |  |
| Very good | 8,369 (63%) |
| Somewhat good | 3,559 (27%) |
| Somewhat bad | 483 (3.7%) |
| Very bad | 214 (1.6%) |
| Does not apply | 507 (3.8%) |
| (Missing) | 73 (0.6%) |
| **Relationship with father** |  |
| Very good | 6,364 (48%) |
| Somewhat good | 3,654 (28%) |
| Somewhat bad | 1,035 (7.8%) |
| Very bad | 756 (5.7%) |
| Does not apply | 1,303 (9.9%) |
| (Missing) | 93 (0.7%) |
| **Parent marital status** |  |
| Parents married | 8,546 (65%) |
| Divorced | 1,384 (10%) |
| Parents were never married | 1,985 (15%) |
| One or both parents had died | 508 (3.8%) |
| (Missing) | 781 (5.9%) |
| **Subjective financial status of family growing up** |  |
| Lived comfortably | 4,998 (38%) |
| Got by | 4,616 (35%) |
| Found it difficult | 2,484 (19%) |
| Found it very difficult | 1,027 (7.8%) |
| (Missing) | 79 (0.6%) |
| **Abuse** |  |
| Yes | 2,606 (20%) |
| No | 10,147 (77%) |
| (Missing) | 451 (3.4%) |
| **Outsider growing up** |  |
| Yes | 1,659 (13%) |
| No | 11,234 (85%) |
| (Missing) | 311 (2.4%) |
| **Self-rated health growing up** |  |
| Excellent | 5,312 (40%) |
| Very good | 3,392 (26%) |
| Good | 2,873 (22%) |
| Fair | 1,368 (10%) |
| Poor | 228 (1.7%) |
| (Missing) | 30 (0.2%) |
| **Immigration status** |  |
| Born in this country | 12,688 (96%) |
| Born in another country | 153 (1.2%) |
| (Missing) | 363 (2.7%) |
| **Age 12 religious service attendance** |  |
| At least 1/week | 6,306 (48%) |
| 1-3/month | 2,491 (19%) |
| <1/month | 2,629 (20%) |
| Never | 1,707 (13%) |
| (Missing) | 71 (0.5%) |
| **Year of birth** |  |
| 1998-2005; age 18-24 | 1,986 (15%) |
| 1993-1998; age 25-29 | 1,468 (11%) |
| 1983-1993; age 30-39 | 2,908 (22%) |
| 1973-1983; age 40-49 | 2,638 (20%) |
| 1963-1973; age 50-59 | 2,131 (16%) |
| 1953-1963; age 60-69 | 1,435 (11%) |
| 1943-1953; age 70-79 | 510 (3.9%) |
| 1943 or earlier; age 80+ | 126 (1.0%) |
| (Missing) | 0 (0%) |
| **Gender** |  |
| Male | 6,320 (48%) |
| Female | 6,820 (52%) |
| Other | 35 (0.3%) |
| (Missing) | 30 (0.2%) |
| **Religious affiliation** |  |
| Christianity | 11,403 (86%) |
| Islam | 15 (0.1%) |
| Hinduism | 1 (<0.1%) |
| Buddhism | 27 (0.2%) |
| Judaism | 40 (0.3%) |
| Sikhism | 0 (0%) |
| Baha'i | 1 (<0.1%) |
| Jainism | 4 (<0.1%) |
| Shinto | 4 (<0.1%) |
| Taoism | 1 (<0.1%) |
| Confucianism | 7 (<0.1%) |
| Primal, Animist, or Folk religion | 17 (0.1%) |
| Spiritism | 336 (2.5%) |
| Umbanda, Candomble, and other African-derived religions | 262 (2.0%) |
| Chinese folk/traditional religion | 0 (0%) |
| Some other religion | 87 (0.7%) |
| No religion/Atheist/Agnostic | 908 (6.9%) |
| (Missing) | 94 (0.7%) |
| **Race/Ethnicity** |  |
| Amarela | 238 (1.8%) |
| Branca | 5,169 (39%) |
| Indígena | 131 (1.0%) |
| Other | 61 (0.5%) |
| Parda | 5,125 (39%) |
| Preta | 1,615 (12%) |
| (Missing) | 865 (6.6%) |
| ^1^n (%) | |

Table S3b. Childhood predictors regression for Brazil

| Variable | Category | Risk-Ratio | RR 95% CI | Global p-value |
| --- | --- | --- | --- | --- |
| Relationship with mother | (Ref: Very bad/somewhat bad) |  |  | 0.828 |
|  | Very good/somewhat good | 0.99 | (0.83,1.17) |  |
| Relationship with father | (Ref: Very bad/somewhat bad) |  |  | 0.871 |
|  | Very good/somewhat good | 1.00 | (0.88,1.13) |  |
| Parent marital status | (Ref: Parents married) |  |  | 0.232 |
|  | Divorced | 1.12 | (0.96,1.31) |  |
|  | Parents were never married | 1.13 | (0.97,1.32) |  |
|  | One or both parents had died | 1.06 | (0.83,1.36) |  |
| Subjective financial status of family growing up | (Ref: Got by) |  |  | 0.021 |
|  | Lived comfortably | 1.00 | (0.88,1.13) |  |
|  | Found it difficult | 1.10 | (0.96,1.25) |  |
|  | Found it very difficult | 1.25 | (1.07,1.46) |  |
| Abuse | (Ref: No) |  |  | <.001 |
|  | Yes | 1.30 | (1.17,1.46) |  |
| Outsider growing up | (Ref: No) |  |  | <.001 |
|  | Yes | 1.24 | (1.09,1.40) |  |
| Self-rated health growing up | (Ref: Good) |  |  | <.001 |
|  | Excellent | 0.72 | (0.63,0.82) |  |
|  | Very good | 0.79 | (0.69,0.92) |  |
|  | Fair | 1.61 | (1.41,1.85) |  |
|  | Poor | 1.86 | (1.50,2.29) |  |
| Immigration status | (Ref: Born in this country) |  |  | 0.062 |
|  | Born in another country | 1.42 | (0.98,2.07) |  |
| Age 12 religious service attendance | (Ref: Never) |  |  | 0.107 |
|  | At least 1/week | 0.95 | (0.81,1.10) |  |
|  | 1-3/month | 1.11 | (0.93,1.32) |  |
|  | < 1/month | 0.97 | (0.82,1.16) |  |
| Year of birth | (Ref: 1998-2005; current age: 18-24) |  |  | <.001 |
|  | 1993-1998; age 25-29 | 1.03 | (0.83,1.27) |  |
|  | 1983-1993; age 30-39 | 1.24 | (1.03,1.49) |  |
|  | 1973-1983; age 40-49 | 1.91 | (1.61,2.26) |  |
|  | 1963-1973; age 50-59 | 2.41 | (2.03,2.87) |  |
|  | 1953-1963; age 60-69 | 2.88 | (2.37,3.50) |  |
|  | 1943-1953; age 70-79 | 3.14 | (2.40,4.10) |  |
|  | 1943 or earlier; age 80+ | 2.96 | (1.89,4.64) |  |
| Gender | (Ref: Male) |  |  | 0.256 |
|  | Female | 0.92 | (0.84,1.02) |  |
|  | Other | 1.07 | (0.57,2.04) |  |
| Religious affiliation | (Ref: No religion/Atheist/Agnostic) |  |  | 0.420 |
|  | Christianity | 1.07 | (0.87,1.30) |  |
|  | Collapsed affiliations with prevalence<3% | 1.18 | (0.91,1.54) |  |
| Race/ethnicity | (Ref: Plurality group) |  |  | 0.577 |
|  | Non-plurality groups | 1.03 | (0.93,1.14) |  |

Table S3c. Sensitivity to unmeasured confounding of childhood predictors in Brazil

| Variable | Category | E-value for Estimate | E-value for 95% CI |
| --- | --- | --- | --- |
| Relationship with mother | (Ref: Very bad/somewhat bad) |  |  |
|  | Very good/somewhat good | 1.14 | 1.00 |
| Relationship with father | (Ref: Very bad/somewhat bad) |  |  |
|  | Very good/somewhat good | 1.07 | 1.00 |
| Parent marital status | (Ref: Parents married) |  |  |
|  | Divorced | 1.49 | 1.00 |
|  | Parents were never married | 1.51 | 1.00 |
|  | One or both parents had died | 1.31 | 1.00 |
| Subjective financial status of family growing up | (Ref: Got by) |  |  |
|  | Lived comfortably | 1.06 | 1.00 |
|  | Found it difficult | 1.42 | 1.00 |
|  | Found it very difficult | 1.81 | 1.35 |
| Abuse | (Ref: No) |  |  |
|  | Yes | 1.93 | 1.61 |
| Outsider growing up | (Ref: No) |  |  |
|  | Yes | 1.78 | 1.41 |
| Self-rated health growing up | (Ref: Good) |  |  |
|  | Excellent | 2.13 | 1.73 |
|  | Very good | 1.83 | 1.41 |
|  | Fair | 2.61 | 2.17 |
|  | Poor | 3.12 | 2.38 |
| Immigration status | (Ref: Born in this country) |  |  |
|  | Born in another country | 2.20 | 1.00 |
| Age 12 religious service attendance | (Ref: Never) |  |  |
|  | At least 1/week | 1.30 | 1.00 |
|  | 1-3/month | 1.46 | 1.00 |
|  | < 1/month | 1.20 | 1.00 |
| Year of birth | (Ref: 1998-2005; current age: 18-24) |  |  |
|  | 1993-1998; age 25-29 | 1.19 | 1.00 |
|  | 1983-1993; age 30-39 | 1.78 | 1.22 |
|  | 1973-1983; age 40-49 | 3.22 | 2.60 |
|  | 1963-1973; age 50-59 | 4.26 | 3.48 |
|  | 1953-1963; age 60-69 | 5.20 | 4.17 |
|  | 1943-1953; age 70-79 | 5.73 | 4.24 |
|  | 1943 or earlier; age 80+ | 5.37 | 3.18 |
| Gender | (Ref: Male) |  |  |
|  | Female | 1.38 | 1.00 |
|  | Other | 1.36 | 1.00 |
| Religious affiliation | (Ref: No religion/Atheist/Agnostic) |  |  |
|  | Christianity | 1.34 | 1.00 |
|  | Collapsed affiliations with prevalence<3% | 1.65 | 1.00 |
| Race/ethnicity | (Ref: Plurality group) |  |  |
|  | Non-plurality groups | 1.19 | 1.00 |

Table S4a. Nationally representative descriptive statistics for Egypt

| **Characteristic** | **N = 4,729**^1^ |
| --- | --- |
| **Relationship with mother** |  |
| Very good | 4,110 (87%) |
| Somewhat good | 505 (11%) |
| Somewhat bad | 21 (0.4%) |
| Very bad | 10 (0.2%) |
| Does not apply | 83 (1.8%) |
| (Missing) | 0 (0%) |
| **Relationship with father** |  |
| Very good | 3,713 (79%) |
| Somewhat good | 683 (14%) |
| Somewhat bad | 56 (1.2%) |
| Very bad | 30 (0.6%) |
| Does not apply | 233 (4.9%) |
| (Missing) | 14 (0.3%) |
| **Parent marital status** |  |
| Parents married | 4,049 (86%) |
| Divorced | 131 (2.8%) |
| Parents were never married | 9 (0.2%) |
| One or both parents had died | 485 (10%) |
| (Missing) | 55 (1.2%) |
| **Subjective financial status of family growing up** |  |
| Lived comfortably | 1,251 (26%) |
| Got by | 2,352 (50%) |
| Found it difficult | 857 (18%) |
| Found it very difficult | 268 (5.7%) |
| (Missing) | 1 (<0.1%) |
| **Abuse** |  |
| Yes | 405 (8.6%) |
| No | 4,293 (91%) |
| (Missing) | 30 (0.6%) |
| **Outsider growing up** |  |
| Yes | 260 (5.5%) |
| No | 4,456 (94%) |
| (Missing) | 13 (0.3%) |
| **Self-rated health growing up** |  |
| Excellent | 2,687 (57%) |
| Very good | 1,174 (25%) |
| Good | 497 (11%) |
| Fair | 265 (5.6%) |
| Poor | 106 (2.2%) |
| (Missing) | 1 (<0.1%) |
| **Immigration status** |  |
| Born in this country | 4,713 (100%) |
| Born in another country | 16 (0.3%) |
| (Missing) | 1 (<0.1%) |
| **Age 12 religious service attendance** |  |
| At least 1/week | 2,307 (49%) |
| 1-3/month | 570 (12%) |
| <1/month | 629 (13%) |
| Never | 1,165 (25%) |
| (Missing) | 57 (1.2%) |
| **Year of birth** |  |
| 1998-2005; age 18-24 | 960 (20%) |
| 1993-1998; age 25-29 | 607 (13%) |
| 1983-1993; age 30-39 | 1,204 (25%) |
| 1973-1983; age 40-49 | 897 (19%) |
| 1963-1973; age 50-59 | 613 (13%) |
| 1953-1963; age 60-69 | 387 (8.2%) |
| 1943-1953; age 70-79 | 54 (1.1%) |
| 1943 or earlier; age 80+ | 7 (0.2%) |
| (Missing) | 0 (0%) |
| **Gender** |  |
| Male | 2,394 (51%) |
| Female | 2,334 (49%) |
| Other | 0 (0%) |
| (Missing) | 0 (<0.1%) |
| **Religious affiliation** |  |
| Christianity | 123 (2.6%) |
| Islam | 4,602 (97%) |
| Hinduism | 0 (0%) |
| Buddhism | 0 (0%) |
| Judaism | 0 (0%) |
| Sikhism | 0 (0%) |
| Baha'i | 0 (0%) |
| Jainism | 1 (<0.1%) |
| Shinto | 0 (0%) |
| Taoism | 0 (<0.1%) |
| Confucianism | 0 (0%) |
| Primal, Animist, or Folk religion | 0 (0%) |
| Spiritism | 0 (0%) |
| Umbanda, Candomble, and other African-derived religions | 0 (0%) |
| Chinese folk/traditional religion | 0 (0%) |
| Some other religion | 0 (0%) |
| No religion/Atheist/Agnostic | 0 (0%) |
| (Missing) | 3 (<0.1%) |
| **Race/Ethnicity** |  |
| Arab | 4,585 (97%) |
| Bedouin Arab | 4 (<0.1%) |
| Greek | 1 (<0.1%) |
| Nubian | 27 (0.6%) |
| Turkish | 9 (0.2%) |
| (Missing) | 102 (2.2%) |
| ^1^n (%) | |

Table S4b. Childhood predictors regression for Egypt

| Variable | Category | Risk-Ratio | RR 95% CI | Global p-value |
| --- | --- | --- | --- | --- |
| Relationship with mother | (Ref: Very bad/somewhat bad) |  |  | 0.804 |
|  | Very good/somewhat good | 1.05 | (0.68,1.63) |  |
| Relationship with father | (Ref: Very bad/somewhat bad) |  |  | 0.593 |
|  | Very good/somewhat good | 1.09 | (0.78,1.53) |  |
| Parent marital status | (Ref: Parents married) |  |  | 0.425 |
|  | Divorced | 1.06 | (0.74,1.51) |  |
|  | Parents were never married | 1.44 | (0.45,4.65) |  |
|  | One or both parents had died | 1.16 | (0.96,1.40) |  |
| Subjective financial status of family growing up | (Ref: Got by) |  |  | <.001 |
|  | Lived comfortably | 0.89 | (0.78,1.02) |  |
|  | Found it difficult | 1.29 | (1.14,1.45) |  |
|  | Found it very difficult | 1.05 | (0.85,1.29) |  |
| Abuse | (Ref: No) |  |  | 0.003 |
|  | Yes | 1.32 | (1.10,1.58) |  |
| Outsider growing up | (Ref: No) |  |  | 0.693 |
|  | Yes | 1.04 | (0.86,1.26) |  |
| Self-rated health growing up | (Ref: Good) |  |  | <.001 |
|  | Excellent | 0.83 | (0.71,0.98) |  |
|  | Very good | 0.94 | (0.79,1.13) |  |
|  | Fair | 0.88 | (0.65,1.17) |  |
|  | Poor | 1.65 | (1.24,2.21) |  |
| Immigration status | (Ref: Born in this country) |  |  | 0.764 |
|  | Born in another country | 0.82 | (0.21,3.09) |  |
| Age 12 religious service attendance | (Ref: Never) |  |  | 0.986 |
|  | At least 1/week | 1.00 | (0.87,1.13) |  |
|  | 1-3/month | 1.01 | (0.84,1.22) |  |
|  | < 1/month | 1.02 | (0.86,1.20) |  |
| Year of birth | (Ref: 1998-2005; current age: 18-24) |  |  | <.001 |
|  | 1993-1998; age 25-29 | 0.91 | (0.64,1.27) |  |
|  | 1983-1993; age 30-39 | 1.54 | (1.17,2.04) |  |
|  | 1973-1983; age 40-49 | 2.24 | (1.72,2.91) |  |
|  | 1963-1973; age 50-59 | 3.09 | (2.37,4.03) |  |
|  | 1953-1963; age 60-69 | 3.59 | (2.67,4.82) |  |
|  | 1943-1953; age 70-79 | 4.81 | (3.33,6.95) |  |
|  | 1943 or earlier; age 80+ | 8.77 | (6.33,12.15) |  |
| Gender | (Ref: Male) |  |  | <.001 |
|  | Female | 1.48 | (1.30,1.68) |  |
| Religious affiliation | (Ref: Islam) |  |  | 0.893 |
|  | Collapsed affiliations with prevalence<3% | 1.02 | (0.72,1.44) |  |
| Race/ethnicity | (Ref: Plurality group) |  |  | 0.630 |
|  | Non-plurality groups | 1.10 | (0.74,1.65) |  |

Table S4c. Sensitivity to unmeasured confounding of childhood predictors in Egypt

| Variable | Category | E-value for Estimate | E-value for 95% CI |
| --- | --- | --- | --- |
| Relationship with mother | (Ref: Very bad/somewhat bad) |  |  |
|  | Very good/somewhat good | 1.29 | 1.00 |
| Relationship with father | (Ref: Very bad/somewhat bad) |  |  |
|  | Very good/somewhat good | 1.41 | 1.00 |
| Parent marital status | (Ref: Parents married) |  |  |
|  | Divorced | 1.31 | 1.00 |
|  | Parents were never married | 2.23 | 1.00 |
|  | One or both parents had died | 1.60 | 1.00 |
| Subjective financial status of family growing up | (Ref: Got by) |  |  |
|  | Lived comfortably | 1.49 | 1.00 |
|  | Found it difficult | 1.89 | 1.54 |
|  | Found it very difficult | 1.28 | 1.00 |
| Abuse | (Ref: No) |  |  |
|  | Yes | 1.97 | 1.44 |
| Outsider growing up | (Ref: No) |  |  |
|  | Yes | 1.24 | 1.00 |
| Self-rated health growing up | (Ref: Good) |  |  |
|  | Excellent | 1.69 | 1.18 |
|  | Very good | 1.32 | 1.00 |
|  | Fair | 1.55 | 1.00 |
|  | Poor | 2.69 | 1.77 |
| Immigration status | (Ref: Born in this country) |  |  |
|  | Born in another country | 1.75 | 1.00 |
| Age 12 religious service attendance | (Ref: Never) |  |  |
|  | At least 1/week | 1.07 | 1.00 |
|  | 1-3/month | 1.13 | 1.00 |
|  | < 1/month | 1.14 | 1.00 |
| Year of birth | (Ref: 1998-2005; current age: 18-24) |  |  |
|  | 1993-1998; age 25-29 | 1.44 | 1.00 |
|  | 1983-1993; age 30-39 | 2.46 | 1.61 |
|  | 1973-1983; age 40-49 | 3.91 | 2.84 |
|  | 1963-1973; age 50-59 | 5.63 | 4.16 |
|  | 1953-1963; age 60-69 | 6.63 | 4.78 |
|  | 1943-1953; age 70-79 | 9.09 | 6.12 |
|  | 1943 or earlier; age 80+ | 17.02 | 12.13 |
| Gender | (Ref: Male) |  |  |
|  | Female | 2.32 | 1.93 |
| Religious affiliation | (Ref: Islam) |  |  |
|  | Collapsed affiliations with prevalence<3% | 1.17 | 1.00 |
| Race/ethnicity | (Ref: Plurality group) |  |  |
|  | Non-plurality groups | 1.44 | 1.00 |

Table S5a. Nationally representative descriptive statistics for Germany

| **Characteristic** | **N = 9,506**^1^ |
| --- | --- |
| **Relationship with mother** |  |
| Very good | 5,497 (58%) |
| Somewhat good | 3,031 (32%) |
| Somewhat bad | 496 (5.2%) |
| Very bad | 187 (2.0%) |
| Does not apply | 241 (2.5%) |
| (Missing) | 54 (0.6%) |
| **Relationship with father** |  |
| Very good | 4,652 (49%) |
| Somewhat good | 3,012 (32%) |
| Somewhat bad | 846 (8.9%) |
| Very bad | 385 (4.0%) |
| Does not apply | 538 (5.7%) |
| (Missing) | 73 (0.8%) |
| **Parent marital status** |  |
| Parents married | 7,620 (80%) |
| Divorced | 927 (9.8%) |
| Parents were never married | 578 (6.1%) |
| One or both parents had died | 245 (2.6%) |
| (Missing) | 136 (1.4%) |
| **Subjective financial status of family growing up** |  |
| Lived comfortably | 3,177 (33%) |
| Got by | 4,508 (47%) |
| Found it difficult | 1,481 (16%) |
| Found it very difficult | 314 (3.3%) |
| (Missing) | 26 (0.3%) |
| **Abuse** |  |
| Yes | 1,086 (11%) |
| No | 8,321 (88%) |
| (Missing) | 99 (1.0%) |
| **Outsider growing up** |  |
| Yes | 1,105 (12%) |
| No | 8,262 (87%) |
| (Missing) | 139 (1.5%) |
| **Self-rated health growing up** |  |
| Excellent | 2,633 (28%) |
| Very good | 3,518 (37%) |
| Good | 2,582 (27%) |
| Fair | 612 (6.4%) |
| Poor | 134 (1.4%) |
| (Missing) | 26 (0.3%) |
| **Immigration status** |  |
| Born in this country | 8,722 (92%) |
| Born in another country | 744 (7.8%) |
| (Missing) | 40 (0.4%) |
| **Age 12 religious service attendance** |  |
| At least 1/week | 1,943 (20%) |
| 1-3/month | 1,899 (20%) |
| <1/month | 2,887 (30%) |
| Never | 2,749 (29%) |
| (Missing) | 27 (0.3%) |
| **Year of birth** |  |
| 1998-2005; age 18-24 | 829 (8.7%) |
| 1993-1998; age 25-29 | 774 (8.1%) |
| 1983-1993; age 30-39 | 1,438 (15%) |
| 1973-1983; age 40-49 | 1,494 (16%) |
| 1963-1973; age 50-59 | 1,729 (18%) |
| 1953-1963; age 60-69 | 1,915 (20%) |
| 1943-1953; age 70-79 | 1,137 (12%) |
| 1943 or earlier; age 80+ | 190 (2.0%) |
| (Missing) | 0 (0%) |
| **Gender** |  |
| Male | 4,641 (49%) |
| Female | 4,843 (51%) |
| Other | 11 (0.1%) |
| (Missing) | 11 (0.1%) |
| **Religious affiliation** |  |
| Christianity | 5,751 (61%) |
| Islam | 350 (3.7%) |
| Hinduism | 15 (0.2%) |
| Buddhism | 25 (0.3%) |
| Judaism | 18 (0.2%) |
| Sikhism | 5 (<0.1%) |
| Baha'i | 2 (<0.1%) |
| Jainism | 1 (<0.1%) |
| Shinto | 0 (0%) |
| Taoism | 0 (0%) |
| Confucianism | 4 (<0.1%) |
| Primal, Animist, or Folk religion | 19 (0.2%) |
| Spiritism | 0 (0%) |
| Umbanda, Candomble, and other African-derived religions | 0 (0%) |
| Chinese folk/traditional religion | 0 (0%) |
| Some other religion | 67 (0.7%) |
| No religion/Atheist/Agnostic | 3,163 (33%) |
| (Missing) | 85 (0.9%) |
| ^1^n (%) | |

Table S5b. Childhood predictors regression for Germany

| Variable | Category | Risk-Ratio | RR 95% CI | Global p-value |
| --- | --- | --- | --- | --- |
| Relationship with mother | (Ref: Very bad/somewhat bad) |  |  | 0.482 |
|  | Very good/somewhat good | 0.96 | (0.84,1.09) |  |
| Relationship with father | (Ref: Very bad/somewhat bad) |  |  | 0.181 |
|  | Very good/somewhat good | 1.08 | (0.96,1.21) |  |
| Parent marital status | (Ref: Parents married) |  |  | 0.419 |
|  | Divorced | 1.03 | (0.90,1.17) |  |
|  | Parents were never married | 0.99 | (0.83,1.18) |  |
|  | One or both parents had died | 0.81 | (0.62,1.06) |  |
| Subjective financial status of family growing up | (Ref: Got by) |  |  | 0.047 |
|  | Lived comfortably | 0.97 | (0.87,1.07) |  |
|  | Found it difficult | 1.13 | (1.01,1.26) |  |
|  | Found it very difficult | 1.17 | (0.99,1.39) |  |
| Abuse | (Ref: No) |  |  | <.001 |
|  | Yes | 1.25 | (1.13,1.39) |  |
| Outsider growing up | (Ref: No) |  |  | <.001 |
|  | Yes | 1.26 | (1.13,1.41) |  |
| Self-rated health growing up | (Ref: Good) |  |  | <.001 |
|  | Excellent | 0.72 | (0.63,0.82) |  |
|  | Very good | 0.91 | (0.82,1.01) |  |
|  | Fair | 1.53 | (1.36,1.74) |  |
|  | Poor | 1.43 | (1.12,1.83) |  |
| Immigration status | (Ref: Born in this country) |  |  | <.001 |
|  | Born in another country | 0.73 | (0.61,0.87) |  |
| Age 12 religious service attendance | (Ref: Never) |  |  | 0.793 |
|  | At least 1/week | 1.02 | (0.92,1.14) |  |
|  | 1-3/month | 1.00 | (0.89,1.13) |  |
|  | < 1/month | 0.97 | (0.87,1.08) |  |
| Year of birth | (Ref: 1998-2005; current age: 18-24) |  |  | <.001 |
|  | 1993-1998; age 25-29 | 0.98 | (0.77,1.26) |  |
|  | 1983-1993; age 30-39 | 1.01 | (0.81,1.25) |  |
|  | 1973-1983; age 40-49 | 1.38 | (1.12,1.70) |  |
|  | 1963-1973; age 50-59 | 1.53 | (1.25,1.87) |  |
|  | 1953-1963; age 60-69 | 1.75 | (1.43,2.14) |  |
|  | 1943-1953; age 70-79 | 1.84 | (1.49,2.27) |  |
|  | 1943 or earlier; age 80+ | 1.62 | (1.19,2.20) |  |
| Gender | (Ref: Male) |  |  | 0.690 |
|  | Female | 1.02 | (0.94,1.10) |  |
|  | Other | 0.87 | (0.30,2.57) |  |
| Religious affiliation | (Ref: No religion/Atheist/Agnostic) |  |  | 0.022 |
|  | Islam | 0.98 | (0.75,1.28) |  |
|  | Christianity | 1.10 | (1.00,1.20) |  |
|  | Collapsed affiliations with prevalence<3% | 1.46 | (1.11,1.93) |  |
| Race/ethnicity | (Ref: Plurality group) |  |  |  |

Table S5c. Sensitivity to unmeasured confounding of childhood predictors in Germany

| Variable | Category | E-value for Estimate | E-value for 95% CI |
| --- | --- | --- | --- |
| Relationship with mother | (Ref: Very bad/somewhat bad) |  |  |
|  | Very good/somewhat good | 1.27 | 1.00 |
| Relationship with father | (Ref: Very bad/somewhat bad) |  |  |
|  | Very good/somewhat good | 1.37 | 1.00 |
| Parent marital status | (Ref: Parents married) |  |  |
|  | Divorced | 1.19 | 1.00 |
|  | Parents were never married | 1.11 | 1.00 |
|  | One or both parents had died | 1.78 | 1.00 |
| Subjective financial status of family growing up | (Ref: Got by) |  |  |
|  | Lived comfortably | 1.23 | 1.00 |
|  | Found it difficult | 1.51 | 1.12 |
|  | Found it very difficult | 1.62 | 1.00 |
| Abuse | (Ref: No) |  |  |
|  | Yes | 1.82 | 1.51 |
| Outsider growing up | (Ref: No) |  |  |
|  | Yes | 1.84 | 1.52 |
| Self-rated health growing up | (Ref: Good) |  |  |
|  | Excellent | 2.13 | 1.75 |
|  | Very good | 1.42 | 1.00 |
|  | Fair | 2.44 | 2.05 |
|  | Poor | 2.21 | 1.47 |
| Immigration status | (Ref: Born in this country) |  |  |
|  | Born in another country | 2.09 | 1.55 |
| Age 12 religious service attendance | (Ref: Never) |  |  |
|  | At least 1/week | 1.18 | 1.00 |
|  | 1-3/month | 1.06 | 1.00 |
|  | < 1/month | 1.21 | 1.00 |
| Year of birth | (Ref: 1998-2005; current age: 18-24) |  |  |
|  | 1993-1998; age 25-29 | 1.16 | 1.00 |
|  | 1983-1993; age 30-39 | 1.09 | 1.00 |
|  | 1973-1983; age 40-49 | 2.11 | 1.49 |
|  | 1963-1973; age 50-59 | 2.42 | 1.80 |
|  | 1953-1963; age 60-69 | 2.89 | 2.21 |
|  | 1943-1953; age 70-79 | 3.08 | 2.34 |
|  | 1943 or earlier; age 80+ | 2.63 | 1.68 |
| Gender | (Ref: Male) |  |  |
|  | Female | 1.15 | 1.00 |
|  | Other | 1.55 | 1.00 |
| Religious affiliation | (Ref: No religion/Atheist/Agnostic) |  |  |
|  | Islam | 1.16 | 1.00 |
|  | Christianity | 1.42 | 1.00 |
|  | Collapsed affiliations with prevalence<3% | 2.28 | 1.46 |
| Race/ethnicity | (Ref: Plurality group) |  |  |

Table S6a. Nationally representative descriptive statistics for Hong Kong

| **Characteristic** | **N = 3,012**^1^ |
| --- | --- |
| **Relationship with mother** |  |
| Very good | 1,077 (36%) |
| Somewhat good | 1,164 (39%) |
| Somewhat bad | 293 (9.7%) |
| Very bad | 49 (1.6%) |
| Does not apply | 426 (14%) |
| (Missing) | 3 (<0.1%) |
| **Relationship with father** |  |
| Very good | 868 (29%) |
| Somewhat good | 1,089 (36%) |
| Somewhat bad | 393 (13%) |
| Very bad | 102 (3.4%) |
| Does not apply | 557 (19%) |
| (Missing) | 3 (0.1%) |
| **Parent marital status** |  |
| Parents married | 2,752 (91%) |
| Divorced | 114 (3.8%) |
| Parents were never married | 40 (1.3%) |
| One or both parents had died | 50 (1.7%) |
| (Missing) | 56 (1.8%) |
| **Subjective financial status of family growing up** |  |
| Lived comfortably | 906 (30%) |
| Got by | 1,527 (51%) |
| Found it difficult | 473 (16%) |
| Found it very difficult | 84 (2.8%) |
| (Missing) | 22 (0.7%) |
| **Abuse** |  |
| Yes | 318 (11%) |
| No | 2,688 (89%) |
| (Missing) | 5 (0.2%) |
| **Outsider growing up** |  |
| Yes | 664 (22%) |
| No | 2,224 (74%) |
| (Missing) | 124 (4.1%) |
| **Self-rated health growing up** |  |
| Excellent | 545 (18%) |
| Very good | 1,073 (36%) |
| Good | 863 (29%) |
| Fair | 426 (14%) |
| Poor | 91 (3.0%) |
| (Missing) | 13 (0.4%) |
| **Immigration status** |  |
| Born in this country | 2,637 (88%) |
| Born in another country | 321 (11%) |
| (Missing) | 53 (1.8%) |
| **Age 12 religious service attendance** |  |
| At least 1/week | 432 (14%) |
| 1-3/month | 528 (18%) |
| <1/month | 753 (25%) |
| Never | 1,295 (43%) |
| (Missing) | 4 (0.1%) |
| **Year of birth** |  |
| 1998-2005; age 18-24 | 217 (7.2%) |
| 1993-1998; age 25-29 | 198 (6.6%) |
| 1983-1993; age 30-39 | 507 (17%) |
| 1973-1983; age 40-49 | 580 (19%) |
| 1963-1973; age 50-59 | 711 (24%) |
| 1953-1963; age 60-69 | 620 (21%) |
| 1943-1953; age 70-79 | 164 (5.5%) |
| 1943 or earlier; age 80+ | 15 (0.5%) |
| (Missing) | 0 (0%) |
| **Gender** |  |
| Male | 1,390 (46%) |
| Female | 1,620 (54%) |
| Other | 2 (<0.1%) |
| (Missing) | 0 (0%) |
| **Religious affiliation** |  |
| Christianity | 715 (24%) |
| Islam | 86 (2.9%) |
| Hinduism | 27 (0.9%) |
| Buddhism | 323 (11%) |
| Judaism | 16 (0.5%) |
| Sikhism | 4 (0.1%) |
| Baha'i | 0 (0%) |
| Jainism | 1 (<0.1%) |
| Shinto | 18 (0.6%) |
| Taoism | 81 (2.7%) |
| Confucianism | 10 (0.3%) |
| Primal, Animist, or Folk religion | 15 (0.5%) |
| Spiritism | 0 (0%) |
| Umbanda, Candomble, and other African-derived religions | 0 (0%) |
| Chinese folk/traditional religion | 108 (3.6%) |
| Some other religion | 5 (0.2%) |
| No religion/Atheist/Agnostic | 1,601 (53%) |
| (Missing) | 1 (<0.1%) |
| **Race/Ethnicity** |  |
| Chinese (Cantonese) | 1,930 (64%) |
| Chinese (Chaoshan) | 201 (6.7%) |
| Chinese (Fujianese) | 117 (3.9%) |
| Chinese (Hakka) | 121 (4.0%) |
| Chinese (Other ethnicity) | 264 (8.8%) |
| Chinese (Shanghainese) | 89 (2.9%) |
| East Asian (Korean, Japanese) | 10 (0.3%) |
| Other | 4 (0.1%) |
| South Asian (Indian, Nepalese, Pakistani) | 17 (0.6%) |
| Southeast Asian (Filipino, Indonesian, Thailand) | 46 (1.5%) |
| Taiwanese | 14 (0.4%) |
| White | 15 (0.5%) |
| (Missing) | 184 (6.1%) |
| ^1^n (%) | |

Table S6b. Childhood predictors regression for Hong Kong

| Variable | Category | Risk-Ratio | RR 95% CI | Global p-value |
| --- | --- | --- | --- | --- |
| Relationship with mother | (Ref: Very bad/somewhat bad) |  |  | 0.382 |
|  | Very good/somewhat good | 0.88 | (0.66,1.18) |  |
| Relationship with father | (Ref: Very bad/somewhat bad) |  |  | 0.933 |
|  | Very good/somewhat good | 1.00 | (0.74,1.35) |  |
| Parent marital status | (Ref: Parents married) |  |  | 0.080 |
|  | Divorced | 0.79 | (0.52,1.20) |  |
|  | Parents were never married | 0.83 | (0.46,1.51) |  |
|  | One or both parents had died | 1.83 | (1.01,3.29) |  |
| Subjective financial status of family growing up | (Ref: Got by) |  |  | 0.574 |
|  | Lived comfortably | 0.86 | (0.68,1.07) |  |
|  | Found it difficult | 0.93 | (0.69,1.27) |  |
|  | Found it very difficult | 0.89 | (0.55,1.46) |  |
| Abuse | (Ref: No) |  |  | <.001 |
|  | Yes | 2.29 | (1.87,2.80) |  |
| Outsider growing up | (Ref: No) |  |  | <.001 |
|  | Yes | 2.21 | (1.79,2.73) |  |
| Self-rated health growing up | (Ref: Good) |  |  | <.001 |
|  | Excellent | 1.05 | (0.73,1.51) |  |
|  | Very good | 1.04 | (0.78,1.39) |  |
|  | Fair | 1.45 | (1.06,1.98) |  |
|  | Poor | 2.56 | (1.70,3.86) |  |
| Immigration status | (Ref: Born in this country) |  |  | 0.906 |
|  | Born in another country | 1.01 | (0.72,1.40) |  |
| Age 12 religious service attendance | (Ref: Never) |  |  | 0.004 |
|  | At least 1/week | 0.82 | (0.57,1.20) |  |
|  | 1-3/month | 1.37 | (1.00,1.86) |  |
|  | < 1/month | 1.10 | (0.83,1.47) |  |
| Year of birth | (Ref: 1998-2005; current age: 18-24) |  |  | <.001 |
|  | 1993-1998; age 25-29 | 1.06 | (0.73,1.54) |  |
|  | 1983-1993; age 30-39 | 1.14 | (0.84,1.55) |  |
|  | 1973-1983; age 40-49 | 1.09 | (0.80,1.48) |  |
|  | 1963-1973; age 50-59 | 1.02 | (0.73,1.42) |  |
|  | 1953-1963; age 60-69 | 1.34 | (0.91,1.97) |  |
|  | 1943-1953; age 70-79 | 2.70 | (1.54,4.75) |  |
|  | 1943 or earlier; age 80+ | 0.00 | (0.00,0.00) |  |
| Gender | (Ref: Male) |  |  | <.001 |
|  | Female | 0.83 | (0.69,1.00) |  |
|  | Other | 0.00 | (0.00,0.00) |  |
| Religious affiliation | (Ref: No religion/Atheist/Agnostic) |  |  | <.001 |
|  | Buddhism | 1.84 | (1.37,2.47) |  |
|  | Chinese folk/traditional religion | 1.06 | (0.58,1.95) |  |
|  | Christianity | 1.46 | (1.08,1.98) |  |
|  | Collapsed affiliations with prevalence<3% | 1.64 | (1.19,2.27) |  |
| Race/ethnicity | (Ref: Plurality group) |  |  | 0.331 |
|  | Non-plurality groups | 0.90 | (0.72,1.12) |  |

Table S6c. Sensitivity to unmeasured confounding of childhood predictors in Hong Kong

| Variable | Category | E-value for Estimate | E-value for 95% CI |
| --- | --- | --- | --- |
| Relationship with mother | (Ref: Very bad/somewhat bad) |  |  |
|  | Very good/somewhat good | 1.52 | 1.00 |
| Relationship with father | (Ref: Very bad/somewhat bad) |  |  |
|  | Very good/somewhat good | 1.03 | 1.00 |
| Parent marital status | (Ref: Parents married) |  |  |
|  | Divorced | 1.83 | 1.00 |
|  | Parents were never married | 1.69 | 1.00 |
|  | One or both parents had died | 3.05 | 1.13 |
| Subjective financial status of family growing up | (Ref: Got by) |  |  |
|  | Lived comfortably | 1.61 | 1.00 |
|  | Found it difficult | 1.34 | 1.00 |
|  | Found it very difficult | 1.48 | 1.00 |
| Abuse | (Ref: No) |  |  |
|  | Yes | 4.01 | 3.15 |
| Outsider growing up | (Ref: No) |  |  |
|  | Yes | 3.84 | 2.98 |
| Self-rated health growing up | (Ref: Good) |  |  |
|  | Excellent | 1.27 | 1.00 |
|  | Very good | 1.24 | 1.00 |
|  | Fair | 2.25 | 1.30 |
|  | Poor | 4.56 | 2.79 |
| Immigration status | (Ref: Born in this country) |  |  |
|  | Born in another country | 1.09 | 1.00 |
| Age 12 religious service attendance | (Ref: Never) |  |  |
|  | At least 1/week | 1.72 | 1.00 |
|  | 1-3/month | 2.07 | 1.07 |
|  | < 1/month | 1.44 | 1.00 |
| Year of birth | (Ref: 1998-2005; current age: 18-24) |  |  |
|  | 1993-1998; age 25-29 | 1.32 | 1.00 |
|  | 1983-1993; age 30-39 | 1.54 | 1.00 |
|  | 1973-1983; age 40-49 | 1.40 | 1.00 |
|  | 1963-1973; age 50-59 | 1.17 | 1.00 |
|  | 1953-1963; age 60-69 | 2.01 | 1.00 |
|  | 1943-1953; age 70-79 | 4.85 | 2.45 |
|  | 1943 or earlier; age 80+ | 1290362.06 | 376262.33 |
| Gender | (Ref: Male) |  |  |
|  | Female | 1.70 | 1.00 |
|  | Other | 1017714.82 | 208411.82 |
| Religious affiliation | (Ref: No religion/Atheist/Agnostic) |  |  |
|  | Buddhism | 3.08 | 2.09 |
|  | Chinese folk/traditional religion | 1.32 | 1.00 |
|  | Christianity | 2.28 | 1.38 |
|  | Collapsed affiliations with prevalence<3% | 2.67 | 1.67 |
| Race/ethnicity | (Ref: Plurality group) |  |  |
|  | Non-plurality groups | 1.46 | 1.00 |

Table S7a. Nationally representative descriptive statistics for India

| **Characteristic** | **N = 12,765**^1^ |
| --- | --- |
| **Relationship with mother** |  |
| Very good | 11,465 (90%) |
| Somewhat good | 788 (6.2%) |
| Somewhat bad | 88 (0.7%) |
| Very bad | 73 (0.6%) |
| Does not apply | 269 (2.1%) |
| (Missing) | 82 (0.6%) |
| **Relationship with father** |  |
| Very good | 10,923 (86%) |
| Somewhat good | 995 (7.8%) |
| Somewhat bad | 126 (1.0%) |
| Very bad | 100 (0.8%) |
| Does not apply | 481 (3.8%) |
| (Missing) | 141 (1.1%) |
| **Parent marital status** |  |
| Parents married | 5,578 (44%) |
| Divorced | 236 (1.8%) |
| Parents were never married | 1,055 (8.3%) |
| One or both parents had died | 940 (7.4%) |
| (Missing) | 4,956 (39%) |
| **Subjective financial status of family growing up** |  |
| Lived comfortably | 4,946 (39%) |
| Got by | 3,010 (24%) |
| Found it difficult | 2,703 (21%) |
| Found it very difficult | 2,035 (16%) |
| (Missing) | 70 (0.5%) |
| **Abuse** |  |
| Yes | 1,468 (11%) |
| No | 10,526 (82%) |
| (Missing) | 771 (6.0%) |
| **Outsider growing up** |  |
| Yes | 1,926 (15%) |
| No | 10,780 (84%) |
| (Missing) | 59 (0.5%) |
| **Self-rated health growing up** |  |
| Excellent | 2,182 (17%) |
| Very good | 3,882 (30%) |
| Good | 4,028 (32%) |
| Fair | 2,202 (17%) |
| Poor | 424 (3.3%) |
| (Missing) | 47 (0.4%) |
| **Immigration status** |  |
| Born in this country | 12,629 (99%) |
| Born in another country | 110 (0.9%) |
| (Missing) | 26 (0.2%) |
| **Age 12 religious service attendance** |  |
| At least 1/week | 5,288 (41%) |
| 1-3/month | 2,959 (23%) |
| <1/month | 2,719 (21%) |
| Never | 1,478 (12%) |
| (Missing) | 321 (2.5%) |
| **Year of birth** |  |
| 1998-2005; age 18-24 | 2,543 (20%) |
| 1993-1998; age 25-29 | 1,640 (13%) |
| 1983-1993; age 30-39 | 3,109 (24%) |
| 1973-1983; age 40-49 | 2,275 (18%) |
| 1963-1973; age 50-59 | 1,574 (12%) |
| 1953-1963; age 60-69 | 1,188 (9.3%) |
| 1943-1953; age 70-79 | 370 (2.9%) |
| 1943 or earlier; age 80+ | 67 (0.5%) |
| (Missing) | 0 (0%) |
| **Gender** |  |
| Male | 6,473 (51%) |
| Female | 6,292 (49%) |
| Other | 0 (0%) |
| (Missing) | 0 (0%) |
| **Religious affiliation** |  |
| Christianity | 254 (2.0%) |
| Islam | 1,550 (12%) |
| Hinduism | 10,417 (82%) |
| Buddhism | 180 (1.4%) |
| Judaism | 0 (0%) |
| Sikhism | 126 (1.0%) |
| Baha'i | 0 (0%) |
| Jainism | 9 (<0.1%) |
| Shinto | 4 (<0.1%) |
| Taoism | 0 (0%) |
| Confucianism | 0 (0%) |
| Primal, Animist, or Folk religion | 27 (0.2%) |
| Spiritism | 0 (0%) |
| Umbanda, Candomble, and other African-derived religions | 0 (0%) |
| Chinese folk/traditional religion | 0 (0%) |
| Some other religion | 59 (0.5%) |
| No religion/Atheist/Agnostic | 7 (<0.1%) |
| (Missing) | 131 (1.0%) |
| **Race/Ethnicity** |  |
| General | 3,538 (28%) |
| Other backward caste | 4,177 (33%) |
| Schedule caste | 3,599 (28%) |
| Schedule tribe | 1,185 (9.3%) |
| (Missing) | 267 (2.1%) |
| ^1^n (%) | |

Table S7b. Childhood predictors regression for India

| Variable | Category | Risk-Ratio | RR 95% CI | Global p-value |
| --- | --- | --- | --- | --- |
| Relationship with mother | (Ref: Very bad/somewhat bad) |  |  | 0.248 |
|  | Very good/somewhat good | 1.14 | (0.91,1.42) |  |
| Relationship with father | (Ref: Very bad/somewhat bad) |  |  | 0.487 |
|  | Very good/somewhat good | 0.93 | (0.75,1.15) |  |
| Parent marital status | (Ref: Parents married) |  |  | <.001 |
|  | Divorced | 1.09 | (0.86,1.38) |  |
|  | Parents were never married | 0.67 | (0.58,0.76) |  |
|  | One or both parents had died | 0.94 | (0.84,1.06) |  |
| Subjective financial status of family growing up | (Ref: Got by) |  |  | 0.053 |
|  | Lived comfortably | 0.93 | (0.85,1.01) |  |
|  | Found it difficult | 1.01 | (0.92,1.11) |  |
|  | Found it very difficult | 1.04 | (0.93,1.15) |  |
| Abuse | (Ref: No) |  |  | <.001 |
|  | Yes | 1.92 | (1.77,2.08) |  |
| Outsider growing up | (Ref: No) |  |  | 0.049 |
|  | Yes | 1.09 | (1.00,1.20) |  |
| Self-rated health growing up | (Ref: Good) |  |  | 0.002 |
|  | Excellent | 0.98 | (0.88,1.10) |  |
|  | Very good | 0.93 | (0.85,1.01) |  |
|  | Fair | 1.09 | (0.99,1.20) |  |
|  | Poor | 1.22 | (1.04,1.43) |  |
| Immigration status | (Ref: Born in this country) |  |  | 0.053 |
|  | Born in another country | 0.69 | (0.47,1.00) |  |
| Age 12 religious service attendance | (Ref: Never) |  |  | 0.041 |
|  | At least 1/week | 1.03 | (0.92,1.16) |  |
|  | 1-3/month | 1.12 | (1.00,1.25) |  |
|  | < 1/month | 1.13 | (1.01,1.27) |  |
| Year of birth | (Ref: 1998-2005; current age: 18-24) |  |  | <.001 |
|  | 1993-1998; age 25-29 | 1.20 | (1.03,1.40) |  |
|  | 1983-1993; age 30-39 | 1.35 | (1.18,1.54) |  |
|  | 1973-1983; age 40-49 | 1.71 | (1.50,1.95) |  |
|  | 1963-1973; age 50-59 | 1.94 | (1.68,2.23) |  |
|  | 1953-1963; age 60-69 | 2.35 | (2.04,2.72) |  |
|  | 1943-1953; age 70-79 | 2.56 | (2.14,3.07) |  |
|  | 1943 or earlier; age 80+ | 2.34 | (1.60,3.43) |  |
| Gender | (Ref: Male) |  |  | <.001 |
|  | Female | 1.26 | (1.18,1.34) |  |
| Religious affiliation | (Ref: Hinduism) |  |  | 0.421 |
|  | Islam | 0.95 | (0.82,1.10) |  |
|  | Collapsed affiliations with prevalence<3% | 0.90 | (0.76,1.07) |  |
| Race/ethnicity | (Ref: Plurality group) |  |  | 0.055 |
|  | Non-plurality groups | 0.93 | (0.86,1.00) |  |

Table S7c. Sensitivity to unmeasured confounding of childhood predictors in India

| Variable | Category | E-value for Estimate | E-value for 95% CI |
| --- | --- | --- | --- |
| Relationship with mother | (Ref: Very bad/somewhat bad) |  |  |
|  | Very good/somewhat good | 1.53 | 1.00 |
| Relationship with father | (Ref: Very bad/somewhat bad) |  |  |
|  | Very good/somewhat good | 1.36 | 1.00 |
| Parent marital status | (Ref: Parents married) |  |  |
|  | Divorced | 1.41 | 1.00 |
|  | Parents were never married | 2.37 | 1.95 |
|  | One or both parents had died | 1.31 | 1.00 |
| Subjective financial status of family growing up | (Ref: Got by) |  |  |
|  | Lived comfortably | 1.37 | 1.00 |
|  | Found it difficult | 1.12 | 1.00 |
|  | Found it very difficult | 1.23 | 1.00 |
| Abuse | (Ref: No) |  |  |
|  | Yes | 3.24 | 2.93 |
| Outsider growing up | (Ref: No) |  |  |
|  | Yes | 1.41 | 1.00 |
| Self-rated health growing up | (Ref: Good) |  |  |
|  | Excellent | 1.16 | 1.00 |
|  | Very good | 1.36 | 1.00 |
|  | Fair | 1.40 | 1.00 |
|  | Poor | 1.74 | 1.25 |
| Immigration status | (Ref: Born in this country) |  |  |
|  | Born in another country | 2.26 | 1.00 |
| Age 12 religious service attendance | (Ref: Never) |  |  |
|  | At least 1/week | 1.22 | 1.00 |
|  | 1-3/month | 1.47 | 1.00 |
|  | < 1/month | 1.51 | 1.11 |
| Year of birth | (Ref: 1998-2005; current age: 18-24) |  |  |
|  | 1993-1998; age 25-29 | 1.68 | 1.19 |
|  | 1983-1993; age 30-39 | 2.03 | 1.65 |
|  | 1973-1983; age 40-49 | 2.82 | 2.38 |
|  | 1963-1973; age 50-59 | 3.29 | 2.76 |
|  | 1953-1963; age 60-69 | 4.14 | 3.49 |
|  | 1943-1953; age 70-79 | 4.57 | 3.70 |
|  | 1943 or earlier; age 80+ | 4.12 | 2.58 |
| Gender | (Ref: Male) |  |  |
|  | Female | 1.83 | 1.65 |
| Religious affiliation | (Ref: Hinduism) |  |  |
|  | Islam | 1.28 | 1.00 |
|  | Collapsed affiliations with prevalence<3% | 1.45 | 1.00 |
| Race/ethnicity | (Ref: Plurality group) |  |  |
|  | Non-plurality groups | 1.36 | 1.00 |

Table S8a. Nationally representative descriptive statistics for Indonesia

| **Characteristic** | **N = 6,992**^1^ |
| --- | --- |
| **Relationship with mother** |  |
| Very good | 6,238 (89%) |
| Somewhat good | 583 (8.3%) |
| Somewhat bad | 50 (0.7%) |
| Very bad | 26 (0.4%) |
| Does not apply | 68 (1.0%) |
| (Missing) | 27 (0.4%) |
| **Relationship with father** |  |
| Very good | 6,067 (87%) |
| Somewhat good | 628 (9.0%) |
| Somewhat bad | 68 (1.0%) |
| Very bad | 52 (0.7%) |
| Does not apply | 115 (1.6%) |
| (Missing) | 61 (0.9%) |
| **Parent marital status** |  |
| Parents married | 5,557 (79%) |
| Divorced | 448 (6.4%) |
| Parents were never married | 47 (0.7%) |
| One or both parents had died | 735 (11%) |
| (Missing) | 205 (2.9%) |
| **Subjective financial status of family growing up** |  |
| Lived comfortably | 3,408 (49%) |
| Got by | 2,955 (42%) |
| Found it difficult | 439 (6.3%) |
| Found it very difficult | 181 (2.6%) |
| (Missing) | 9 (0.1%) |
| **Abuse** |  |
| Yes | 486 (6.9%) |
| No | 6,427 (92%) |
| (Missing) | 79 (1.1%) |
| **Outsider growing up** |  |
| Yes | 343 (4.9%) |
| No | 6,639 (95%) |
| (Missing) | 10 (0.1%) |
| **Self-rated health growing up** |  |
| Excellent | 1,246 (18%) |
| Very good | 1,968 (28%) |
| Good | 2,490 (36%) |
| Fair | 1,233 (18%) |
| Poor | 55 (0.8%) |
| (Missing) | 1 (<0.1%) |
| **Immigration status** |  |
| Born in this country | 6,958 (100%) |
| Born in another country | 34 (0.5%) |
| (Missing) | 0 (0%) |
| **Age 12 religious service attendance** |  |
| At least 1/week | 5,363 (77%) |
| 1-3/month | 973 (14%) |
| <1/month | 329 (4.7%) |
| Never | 275 (3.9%) |
| (Missing) | 51 (0.7%) |
| **Year of birth** |  |
| 1998-2005; age 18-24 | 1,216 (17%) |
| 1993-1998; age 25-29 | 849 (12%) |
| 1983-1993; age 30-39 | 1,591 (23%) |
| 1973-1983; age 40-49 | 1,576 (23%) |
| 1963-1973; age 50-59 | 1,169 (17%) |
| 1953-1963; age 60-69 | 490 (7.0%) |
| 1943-1953; age 70-79 | 83 (1.2%) |
| 1943 or earlier; age 80+ | 17 (0.2%) |
| (Missing) | 0 (0%) |
| **Gender** |  |
| Male | 3,461 (50%) |
| Female | 3,513 (50%) |
| Other | 7 (<0.1%) |
| (Missing) | 11 (0.2%) |
| **Religious affiliation** |  |
| Christianity | 528 (7.6%) |
| Islam | 6,373 (91%) |
| Hinduism | 75 (1.1%) |
| Buddhism | 5 (<0.1%) |
| Judaism | 0 (0%) |
| Sikhism | 0 (0%) |
| Baha'i | 0 (0%) |
| Jainism | 1 (<0.1%) |
| Shinto | 0 (0%) |
| Taoism | 0 (<0.1%) |
| Confucianism | 1 (<0.1%) |
| Primal, Animist, or Folk religion | 1 (<0.1%) |
| Spiritism | 0 (0%) |
| Umbanda, Candomble, and other African-derived religions | 0 (0%) |
| Chinese folk/traditional religion | 0 (0%) |
| Some other religion | 0 (0%) |
| No religion/Atheist/Agnostic | 2 (<0.1%) |
| (Missing) | 8 (0.1%) |
| **Race/Ethnicity** |  |
| Bali | 69 (1.0%) |
| Banjar/Melayu Banjar | 320 (4.6%) |
| Batak | 165 (2.4%) |
| Betawi | 251 (3.6%) |
| Bugis | 243 (3.5%) |
| Jawa | 2,846 (41%) |
| Madura | 262 (3.7%) |
| Makasar | 91 (1.3%) |
| Minangkabau | 273 (3.9%) |
| Other | 1,262 (18%) |
| Sunda/Parahyangan | 1,172 (17%) |
| (Missing) | 38 (0.5%) |
| ^1^n (%) | |

Table S8b. Childhood predictors regression for Indonesia

| Variable | Category | Risk-Ratio | RR 95% CI | Global p-value |
| --- | --- | --- | --- | --- |
| Relationship with mother | (Ref: Very bad/somewhat bad) |  |  | 0.449 |
|  | Very good/somewhat good | 0.87 | (0.58,1.30) |  |
| Relationship with father | (Ref: Very bad/somewhat bad) |  |  | 0.044 |
|  | Very good/somewhat good | 1.48 | (1.00,2.17) |  |
| Parent marital status | (Ref: Parents married) |  |  | <.001 |
|  | Divorced | 1.63 | (1.30,2.05) |  |
|  | Parents were never married | 1.90 | (1.10,3.28) |  |
|  | One or both parents had died | 1.43 | (1.19,1.72) |  |
| Subjective financial status of family growing up | (Ref: Got by) |  |  | 0.005 |
|  | Lived comfortably | 0.82 | (0.71,0.96) |  |
|  | Found it difficult | 0.72 | (0.53,0.96) |  |
|  | Found it very difficult | 1.10 | (0.73,1.65) |  |
| Abuse | (Ref: No) |  |  | <.001 |
|  | Yes | 2.18 | (1.80,2.63) |  |
| Outsider growing up | (Ref: No) |  |  | 0.009 |
|  | Yes | 1.40 | (1.09,1.80) |  |
| Self-rated health growing up | (Ref: Good) |  |  | 0.045 |
|  | Excellent | 0.88 | (0.71,1.09) |  |
|  | Very good | 0.89 | (0.73,1.07) |  |
|  | Fair | 1.18 | (0.98,1.42) |  |
|  | Poor | 0.98 | (0.45,2.15) |  |
| Immigration status | (Ref: Born in this country) |  |  | 0.023 |
|  | Born in another country | 1.67 | (1.06,2.65) |  |
| Age 12 religious service attendance | (Ref: Never) |  |  | 0.902 |
|  | At least 1/week | 0.94 | (0.68,1.29) |  |
|  | 1-3/month | 1.00 | (0.70,1.41) |  |
|  | < 1/month | 0.92 | (0.59,1.45) |  |
| Year of birth | (Ref: 1998-2005; current age: 18-24) |  |  | <.001 |
|  | 1993-1998; age 25-29 | 0.73 | (0.53,0.99) |  |
|  | 1983-1993; age 30-39 | 0.83 | (0.63,1.09) |  |
|  | 1973-1983; age 40-49 | 1.24 | (1.00,1.55) |  |
|  | 1963-1973; age 50-59 | 1.92 | (1.51,2.44) |  |
|  | 1953-1963; age 60-69 | 2.08 | (1.55,2.81) |  |
|  | 1943-1953; age 70-79 | 3.35 | (2.16,5.20) |  |
|  | 1943 or earlier; age 80+ | 1.58 | (0.27,9.38) |  |
| Gender | (Ref: Male) |  |  | 0.771 |
|  | Female | 0.98 | (0.84,1.14) |  |
|  | Other | 0.49 | (0.06,3.86) |  |
| Religious affiliation | (Ref: Islam) |  |  | 0.002 |
|  | Christianity | 1.40 | (1.15,1.70) |  |
|  | Collapsed affiliations with prevalence<3% | 0.95 | (0.43,2.14) |  |
| Race/ethnicity | (Ref: Plurality group) |  |  | <.001 |
|  | Non-plurality groups | 1.33 | (1.14,1.54) |  |

Table S8c. Sensitivity to unmeasured confounding of childhood predictors in Indonesia

| Variable | Category | E-value for Estimate | E-value for 95% CI |
| --- | --- | --- | --- |
| Relationship with mother | (Ref: Very bad/somewhat bad) |  |  |
|  | Very good/somewhat good | 1.58 | 1.00 |
| Relationship with father | (Ref: Very bad/somewhat bad) |  |  |
|  | Very good/somewhat good | 2.32 | 1.07 |
| Parent marital status | (Ref: Parents married) |  |  |
|  | Divorced | 2.65 | 1.92 |
|  | Parents were never married | 3.21 | 1.44 |
|  | One or both parents had died | 2.22 | 1.66 |
| Subjective financial status of family growing up | (Ref: Got by) |  |  |
|  | Lived comfortably | 1.72 | 1.25 |
|  | Found it difficult | 2.14 | 1.25 |
|  | Found it very difficult | 1.43 | 1.00 |
| Abuse | (Ref: No) |  |  |
|  | Yes | 3.78 | 3.00 |
| Outsider growing up | (Ref: No) |  |  |
|  | Yes | 2.14 | 1.39 |
| Self-rated health growing up | (Ref: Good) |  |  |
|  | Excellent | 1.54 | 1.00 |
|  | Very good | 1.51 | 1.00 |
|  | Fair | 1.63 | 1.00 |
|  | Poor | 1.14 | 1.00 |
| Immigration status | (Ref: Born in this country) |  |  |
|  | Born in another country | 2.74 | 1.30 |
| Age 12 religious service attendance | (Ref: Never) |  |  |
|  | At least 1/week | 1.34 | 1.00 |
|  | 1-3/month | 1.07 | 1.00 |
|  | < 1/month | 1.38 | 1.00 |
| Year of birth | (Ref: 1998-2005; current age: 18-24) |  |  |
|  | 1993-1998; age 25-29 | 2.10 | 1.09 |
|  | 1983-1993; age 30-39 | 1.71 | 1.00 |
|  | 1973-1983; age 40-49 | 1.79 | 1.00 |
|  | 1963-1973; age 50-59 | 3.25 | 2.38 |
|  | 1953-1963; age 60-69 | 3.59 | 2.46 |
|  | 1943-1953; age 70-79 | 6.16 | 3.74 |
|  | 1943 or earlier; age 80+ | 2.53 | 1.00 |
| Gender | (Ref: Male) |  |  |
|  | Female | 1.18 | 1.00 |
|  | Other | 3.46 | 1.00 |
| Religious affiliation | (Ref: Islam) |  |  |
|  | Christianity | 2.15 | 1.57 |
|  | Collapsed affiliations with prevalence<3% | 1.27 | 1.00 |
| Race/ethnicity | (Ref: Plurality group) |  |  |
|  | Non-plurality groups | 1.98 | 1.54 |

Table 9a. Nationally representative descriptive statistics for Israel

| **Characteristic** | **N = 3,669**^1^ |
| --- | --- |
| **Relationship with mother** |  |
| Very good | 2,686 (73%) |
| Somewhat good | 793 (22%) |
| Somewhat bad | 110 (3.0%) |
| Very bad | 18 (0.5%) |
| Does not apply | 45 (1.2%) |
| (Missing) | 17 (0.5%) |
| **Relationship with father** |  |
| Very good | 2,290 (62%) |
| Somewhat good | 912 (25%) |
| Somewhat bad | 234 (6.4%) |
| Very bad | 37 (1.0%) |
| Does not apply | 171 (4.7%) |
| (Missing) | 25 (0.7%) |
| **Parent marital status** |  |
| Parents married | 3,172 (86%) |
| Divorced | 284 (7.8%) |
| Parents were never married | 36 (1.0%) |
| One or both parents had died | 130 (3.5%) |
| (Missing) | 47 (1.3%) |
| **Subjective financial status of family growing up** |  |
| Lived comfortably | 923 (25%) |
| Got by | 1,822 (50%) |
| Found it difficult | 667 (18%) |
| Found it very difficult | 239 (6.5%) |
| (Missing) | 17 (0.5%) |
| **Abuse* (not administered)** |  |
| Yes | 0 (0%) |
| No | 0 (0%) |
| (Missing) | 3,669 (100%) |
| **Outsider growing up** |  |
| Yes | 371 (10%) |
| No | 3,228 (88%) |
| (Missing) | 70 (1.9%) |
| **Self-rated health growing up** |  |
| Excellent | 1,785 (49%) |
| Very good | 1,284 (35%) |
| Good | 480 (13%) |
| Fair | 105 (2.9%) |
| Poor | 6 (0.2%) |
| (Missing) | 8 (0.2%) |
| **Immigration status** |  |
| Born in this country | 2,796 (76%) |
| Born in another country | 868 (24%) |
| (Missing) | 5 (0.1%) |
| **Age 12 religious service attendance** |  |
| At least 1/week | 867 (24%) |
| 1-3/month | 435 (12%) |
| <1/month | 810 (22%) |
| Never | 1,539 (42%) |
| (Missing) | 17 (0.5%) |
| **Year of birth** |  |
| 1998-2005; age 18-24 | 553 (15%) |
| 1993-1998; age 25-29 | 407 (11%) |
| 1983-1993; age 30-39 | 666 (18%) |
| 1973-1983; age 40-49 | 616 (17%) |
| 1963-1973; age 50-59 | 542 (15%) |
| 1953-1963; age 60-69 | 469 (13%) |
| 1943-1953; age 70-79 | 336 (9.2%) |
| 1943 or earlier; age 80+ | 79 (2.2%) |
| (Missing) | 0 (0%) |
| **Gender** |  |
| Male | 1,791 (49%) |
| Female | 1,872 (51%) |
| Other | 0 (<0.1%) |
| (Missing) | 6 (0.2%) |
| **Religious affiliation** |  |
| Christianity | 60 (1.6%) |
| Islam | 647 (18%) |
| Hinduism | 0 (0%) |
| Buddhism | 0 (0%) |
| Judaism | 2,873 (78%) |
| Sikhism | 1 (<0.1%) |
| Baha'i | 1 (<0.1%) |
| Jainism | 0 (0%) |
| Shinto | 0 (0%) |
| Taoism | 0 (0%) |
| Confucianism | 0 (0%) |
| Primal, Animist, or Folk religion | 3 (<0.1%) |
| Spiritism | 0 (0%) |
| Umbanda, Candomble, and other African-derived religions | 0 (0%) |
| Chinese folk/traditional religion | 0 (0%) |
| Some other religion | 5 (0.1%) |
| No religion/Atheist/Agnostic | 69 (1.9%) |
| (Missing) | 10 (0.3%) |
| **Race/Ethnicity** |  |
| Arab | 674 (18%) |
| Jewish | 2,926 (80%) |
| Other | 39 (1.1%) |
| (Missing) | 30 (0.8%) |
| ^1^n (%) | |

Table S9b. Childhood predictors regression for Israel

| Variable | Category | Risk-Ratio | RR 95% CI | Global p-value |
| --- | --- | --- | --- | --- |
| Relationship with mother | (Ref: Very bad/somewhat bad) |  |  | 0.057 |
|  | Very good/somewhat good | 1.54 | (0.97,2.45) |  |
| Relationship with father | (Ref: Very bad/somewhat bad) |  |  | 0.471 |
|  | Very good/somewhat good | 1.15 | (0.77,1.72) |  |
| Parent marital status | (Ref: Parents married) |  |  | <.001 |
|  | Divorced | 1.06 | (0.72,1.55) |  |
|  | Parents were never married | 3.55 | (2.27,5.53) |  |
|  | One or both parents had died | 1.17 | (0.79,1.73) |  |
| Subjective financial status of family growing up | (Ref: Got by) |  |  | 0.053 |
|  | Lived comfortably | 1.28 | (0.98,1.68) |  |
|  | Found it difficult | 1.13 | (0.89,1.43) |  |
|  | Found it very difficult | 1.39 | (1.06,1.84) |  |
| Outsider growing up | (Ref: No) |  |  | 0.021 |
|  | Yes | 1.38 | (1.05,1.80) |  |
| Self-rated health growing up | (Ref: Good) |  |  | <.001 |
|  | Excellent | 0.53 | (0.40,0.71) |  |
|  | Very good | 0.61 | (0.47,0.79) |  |
|  | Fair | 1.11 | (0.78,1.60) |  |
|  | Poor | 0.78 | (0.21,2.91) |  |
| Immigration status | (Ref: Born in this country) |  |  | 0.735 |
|  | Born in another country | 1.05 | (0.78,1.42) |  |
| Age 12 religious service attendance | (Ref: Never) |  |  | 0.129 |
|  | At least 1/week | 1.21 | (0.96,1.52) |  |
|  | 1-3/month | 0.88 | (0.62,1.25) |  |
|  | < 1/month | 0.92 | (0.72,1.17) |  |
| Year of birth | (Ref: 1998-2005; current age: 18-24) |  |  | <.001 |
|  | 1993-1998; age 25-29 | 1.48 | (0.92,2.41) |  |
|  | 1983-1993; age 30-39 | 1.12 | (0.67,1.88) |  |
|  | 1973-1983; age 40-49 | 1.70 | (1.06,2.73) |  |
|  | 1963-1973; age 50-59 | 2.47 | (1.56,3.92) |  |
|  | 1953-1963; age 60-69 | 3.50 | (2.21,5.56) |  |
|  | 1943-1953; age 70-79 | 5.75 | (3.69,8.95) |  |
|  | 1943 or earlier; age 80+ | 6.25 | (3.57,10.94) |  |
| Gender | (Ref: Male) |  |  | <.001 |
|  | Female | 1.13 | (0.95,1.33) |  |
|  | Other | 0.00 | (0.00,0.00) |  |
| Religious affiliation | (Ref: Judaism) |  |  | 0.050 |
|  | Islam | 2.06 | (1.00,4.24) |  |
|  | Collapsed affiliations with prevalence<3% | 1.15 | (0.72,1.85) |  |
| Race/ethnicity | (Ref: Plurality group) |  |  | 0.770 |
|  | Non-plurality groups | 1.07 | (0.53,2.19) |  |

Table S9c. Sensitivity to unmeasured confounding of childhood predictors in Israel

| Variable | Category | E-value for Estimate | E-value for 95% CI |
| --- | --- | --- | --- |
| Relationship with mother | (Ref: Very bad/somewhat bad) |  |  |
|  | Very good/somewhat good | 2.45 | 1.00 |
| Relationship with father | (Ref: Very bad/somewhat bad) |  |  |
|  | Very good/somewhat good | 1.57 | 1.00 |
| Parent marital status | (Ref: Parents married) |  |  |
|  | Divorced | 1.30 | 1.00 |
|  | Parents were never married | 6.55 | 3.98 |
|  | One or both parents had died | 1.62 | 1.00 |
| Subjective financial status of family growing up | (Ref: Got by) |  |  |
|  | Lived comfortably | 1.89 | 1.00 |
|  | Found it difficult | 1.51 | 1.00 |
|  | Found it very difficult | 2.13 | 1.30 |
| Outsider growing up | (Ref: No) |  |  |
|  | Yes | 2.10 | 1.29 |
| Self-rated health growing up | (Ref: Good) |  |  |
|  | Excellent | 3.16 | 2.17 |
|  | Very good | 2.65 | 1.83 |
|  | Fair | 1.47 | 1.00 |
|  | Poor | 1.90 | 1.00 |
| Immigration status | (Ref: Born in this country) |  |  |
|  | Born in another country | 1.28 | 1.00 |
| Age 12 religious service attendance | (Ref: Never) |  |  |
|  | At least 1/week | 1.71 | 1.00 |
|  | 1-3/month | 1.53 | 1.00 |
|  | < 1/month | 1.40 | 1.00 |
| Year of birth | (Ref: 1998-2005; current age: 18-24) |  |  |
|  | 1993-1998; age 25-29 | 2.33 | 1.00 |
|  | 1983-1993; age 30-39 | 1.49 | 1.00 |
|  | 1973-1983; age 40-49 | 2.80 | 1.32 |
|  | 1963-1973; age 50-59 | 4.38 | 2.50 |
|  | 1953-1963; age 60-69 | 6.47 | 3.84 |
|  | 1943-1953; age 70-79 | 10.97 | 6.84 |
|  | 1943 or earlier; age 80+ | 11.97 | 6.59 |
| Gender | (Ref: Male) |  |  |
|  | Female | 1.51 | 1.00 |
|  | Other | 11954.56 | 1537.66 |
| Religious affiliation | (Ref: Judaism) |  |  |
|  | Islam | 3.54 | 1.05 |
|  | Collapsed affiliations with prevalence<3% | 1.58 | 1.00 |
| Race/ethnicity | (Ref: Plurality group) |  |  |
|  | Non-plurality groups | 1.35 | 1.00 |

Table S10a. Nationally representative descriptive statistics for Japan

| **Characteristic** | **N = 20,543**^1^ |
| --- | --- |
| **Relationship with mother** |  |
| Very good | 5,630 (27%) |
| Somewhat good | 9,461 (46%) |
| Somewhat bad | 2,750 (13%) |
| Very bad | 799 (3.9%) |
| Does not apply | 1,838 (8.9%) |
| (Missing) | 66 (0.3%) |
| **Relationship with father** |  |
| Very good | 4,156 (20%) |
| Somewhat good | 9,081 (44%) |
| Somewhat bad | 3,446 (17%) |
| Very bad | 1,223 (6.0%) |
| Does not apply | 2,580 (13%) |
| (Missing) | 57 (0.3%) |
| **Parent marital status** |  |
| Parents married | 17,713 (86%) |
| Divorced | 1,127 (5.5%) |
| Parents were never married | 591 (2.9%) |
| One or both parents had died | 754 (3.7%) |
| (Missing) | 359 (1.7%) |
| **Subjective financial status of family growing up** |  |
| Lived comfortably | 8,320 (41%) |
| Got by | 8,799 (43%) |
| Found it difficult | 2,398 (12%) |
| Found it very difficult | 973 (4.7%) |
| (Missing) | 52 (0.3%) |
| **Abuse** |  |
| Yes | 1,482 (7.2%) |
| No | 18,964 (92%) |
| (Missing) | 96 (0.5%) |
| **Outsider growing up** |  |
| Yes | 1,963 (9.6%) |
| No | 17,136 (83%) |
| (Missing) | 1,444 (7.0%) |
| **Self-rated health growing up** |  |
| Excellent | 2,711 (13%) |
| Very good | 7,106 (35%) |
| Good | 6,689 (33%) |
| Fair | 3,199 (16%) |
| Poor | 758 (3.7%) |
| (Missing) | 80 (0.4%) |
| **Immigration status** |  |
| Born in this country | 19,548 (95%) |
| Born in another country | 158 (0.8%) |
| (Missing) | 837 (4.1%) |
| **Age 12 religious service attendance** |  |
| At least 1/week | 398 (1.9%) |
| 1-3/month | 883 (4.3%) |
| <1/month | 5,023 (24%) |
| Never | 14,117 (69%) |
| (Missing) | 123 (0.6%) |
| **Year of birth** |  |
| 1998-2005; age 18-24 | 1,589 (7.7%) |
| 1993-1998; age 25-29 | 806 (3.9%) |
| 1983-1993; age 30-39 | 2,851 (14%) |
| 1973-1983; age 40-49 | 3,363 (16%) |
| 1963-1973; age 50-59 | 3,770 (18%) |
| 1953-1963; age 60-69 | 4,118 (20%) |
| 1943-1953; age 70-79 | 3,554 (17%) |
| 1943 or earlier; age 80+ | 493 (2.4%) |
| (Missing) | 0 (0%) |
| **Gender** |  |
| Male | 9,847 (48%) |
| Female | 10,602 (52%) |
| Other | 28 (0.1%) |
| (Missing) | 66 (0.3%) |
| **Religious affiliation** |  |
| Christianity | 343 (1.7%) |
| Islam | 7 (<0.1%) |
| Hinduism | 4 (<0.1%) |
| Buddhism | 6,536 (32%) |
| Judaism | 0 (0%) |
| Sikhism | 0 (0%) |
| Baha'i | 7 (<0.1%) |
| Jainism | 1 (<0.1%) |
| Shinto | 382 (1.9%) |
| Taoism | 14 (<0.1%) |
| Confucianism | 25 (0.1%) |
| Primal, Animist, or Folk religion | 13 (<0.1%) |
| Spiritism | 0 (0%) |
| Umbanda, Candomble, and other African-derived religions | 0 (0%) |
| Chinese folk/traditional religion | 0 (0%) |
| Some other religion | 46 (0.2%) |
| No religion/Atheist/Agnostic | 12,950 (63%) |
| (Missing) | 215 (1.0%) |
| ^1^n (%) | |

Table S10b. Childhood predictors regression for Japan

| Variable | Category | Risk-Ratio | RR 95% CI | Global p-value |
| --- | --- | --- | --- | --- |
| Relationship with mother | (Ref: Very bad/somewhat bad) |  |  | 0.123 |
|  | Very good/somewhat good | 1.08 | (0.98,1.18) |  |
| Relationship with father | (Ref: Very bad/somewhat bad) |  |  | 0.271 |
|  | Very good/somewhat good | 1.05 | (0.96,1.15) |  |
| Parent marital status | (Ref: Parents married) |  |  | 0.005 |
|  | Divorced | 1.00 | (0.86,1.17) |  |
|  | Parents were never married | 1.33 | (1.12,1.58) |  |
|  | One or both parents had died | 1.13 | (0.95,1.33) |  |
| Subjective financial status of family growing up | (Ref: Got by) |  |  | 0.087 |
|  | Lived comfortably | 0.94 | (0.87,1.02) |  |
|  | Found it difficult | 1.06 | (0.96,1.17) |  |
|  | Found it very difficult | 1.09 | (0.95,1.24) |  |
| Abuse | (Ref: No) |  |  | <.001 |
|  | Yes | 1.73 | (1.56,1.92) |  |
| Outsider growing up | (Ref: No) |  |  | <.001 |
|  | Yes | 1.36 | (1.21,1.53) |  |
| Self-rated health growing up | (Ref: Good) |  |  | <.001 |
|  | Excellent | 0.71 | (0.62,0.81) |  |
|  | Very good | 0.81 | (0.74,0.89) |  |
|  | Fair | 1.50 | (1.37,1.64) |  |
|  | Poor | 2.32 | (2.07,2.61) |  |
| Immigration status | (Ref: Born in this country) |  |  | 0.929 |
|  | Born in another country | 1.01 | (0.70,1.45) |  |
| Age 12 religious service attendance | (Ref: Never) |  |  | <.001 |
|  | At least 1/week | 1.21 | (0.98,1.50) |  |
|  | 1-3/month | 1.37 | (1.19,1.58) |  |
|  | < 1/month | 1.10 | (1.01,1.19) |  |
| Year of birth | (Ref: 1998-2005; current age: 18-24) |  |  | <.001 |
|  | 1993-1998; age 25-29 | 0.81 | (0.65,1.01) |  |
|  | 1983-1993; age 30-39 | 0.89 | (0.76,1.05) |  |
|  | 1973-1983; age 40-49 | 1.06 | (0.91,1.24) |  |
|  | 1963-1973; age 50-59 | 1.16 | (0.99,1.34) |  |
|  | 1953-1963; age 60-69 | 1.39 | (1.20,1.61) |  |
|  | 1943-1953; age 70-79 | 1.49 | (1.28,1.74) |  |
|  | 1943 or earlier; age 80+ | 1.79 | (1.45,2.22) |  |
| Gender | (Ref: Male) |  |  | 0.005 |
|  | Female | 0.90 | (0.84,0.96) |  |
|  | Other | 1.27 | (0.76,2.12) |  |
| Religious affiliation | (Ref: No religion/Atheist/Agnostic) |  |  | <.001 |
|  | Buddhism | 1.10 | (1.02,1.18) |  |
|  | Collapsed affiliations with prevalence<3% | 1.29 | (1.11,1.51) |  |
| Race/ethnicity | (Ref: Plurality group) |  |  |  |

Table S10c. Sensitivity to unmeasured confounding of childhood predictors in Japan

| Variable | Category | E-value for Estimate | E-value for 95% CI |
| --- | --- | --- | --- |
| Relationship with mother | (Ref: Very bad/somewhat bad) |  |  |
|  | Very good/somewhat good | 1.36 | 1.00 |
| Relationship with father | (Ref: Very bad/somewhat bad) |  |  |
|  | Very good/somewhat good | 1.28 | 1.00 |
| Parent marital status | (Ref: Parents married) |  |  |
|  | Divorced | 1.05 | 1.00 |
|  | Parents were never married | 2.00 | 1.49 |
|  | One or both parents had died | 1.50 | 1.00 |
| Subjective financial status of family growing up | (Ref: Got by) |  |  |
|  | Lived comfortably | 1.33 | 1.00 |
|  | Found it difficult | 1.30 | 1.00 |
|  | Found it very difficult | 1.40 | 1.00 |
| Abuse | (Ref: No) |  |  |
|  | Yes | 2.85 | 2.50 |
| Outsider growing up | (Ref: No) |  |  |
|  | Yes | 2.05 | 1.71 |
| Self-rated health growing up | (Ref: Good) |  |  |
|  | Excellent | 2.19 | 1.78 |
|  | Very good | 1.77 | 1.50 |
|  | Fair | 2.37 | 2.09 |
|  | Poor | 4.08 | 3.55 |
| Immigration status | (Ref: Born in this country) |  |  |
|  | Born in another country | 1.08 | 1.00 |
| Age 12 religious service attendance | (Ref: Never) |  |  |
|  | At least 1/week | 1.72 | 1.00 |
|  | 1-3/month | 2.09 | 1.67 |
|  | < 1/month | 1.42 | 1.13 |
| Year of birth | (Ref: 1998-2005; current age: 18-24) |  |  |
|  | 1993-1998; age 25-29 | 1.76 | 1.00 |
|  | 1983-1993; age 30-39 | 1.49 | 1.00 |
|  | 1973-1983; age 40-49 | 1.31 | 1.00 |
|  | 1963-1973; age 50-59 | 1.58 | 1.00 |
|  | 1953-1963; age 60-69 | 2.13 | 1.69 |
|  | 1943-1953; age 70-79 | 2.35 | 1.89 |
|  | 1943 or earlier; age 80+ | 2.99 | 2.25 |
| Gender | (Ref: Male) |  |  |
|  | Female | 1.45 | 1.23 |
|  | Other | 1.85 | 1.00 |
| Religious affiliation | (Ref: No religion/Atheist/Agnostic) |  |  |
|  | Buddhism | 1.43 | 1.17 |
|  | Collapsed affiliations with prevalence<3% | 1.91 | 1.45 |
| Race/ethnicity | (Ref: Plurality group) |  |  |

Table S11a. Nationally representative descriptive statistics for Kenya

| **Characteristic** | **N = 11,389**^1^ |
| --- | --- |
| **Relationship with mother** |  |
| Very good | 9,418 (83%) |
| Somewhat good | 1,435 (13%) |
| Somewhat bad | 130 (1.1%) |
| Very bad | 100 (0.9%) |
| Does not apply | 240 (2.1%) |
| (Missing) | 66 (0.6%) |
| **Relationship with father** |  |
| Very good | 7,958 (70%) |
| Somewhat good | 1,896 (17%) |
| Somewhat bad | 216 (1.9%) |
| Very bad | 220 (1.9%) |
| Does not apply | 967 (8.5%) |
| (Missing) | 132 (1.2%) |
| **Parent marital status** |  |
| Parents married | 9,238 (81%) |
| Divorced | 697 (6.1%) |
| Parents were never married | 681 (6.0%) |
| One or both parents had died | 471 (4.1%) |
| (Missing) | 301 (2.6%) |
| **Subjective financial status of family growing up** |  |
| Lived comfortably | 3,026 (27%) |
| Got by | 3,279 (29%) |
| Found it difficult | 4,071 (36%) |
| Found it very difficult | 994 (8.7%) |
| (Missing) | 19 (0.2%) |
| **Abuse** |  |
| Yes | 1,300 (11%) |
| No | 10,039 (88%) |
| (Missing) | 49 (0.4%) |
| **Outsider growing up** |  |
| Yes | 1,223 (11%) |
| No | 10,114 (89%) |
| (Missing) | 52 (0.5%) |
| **Self-rated health growing up** |  |
| Excellent | 4,449 (39%) |
| Very good | 2,598 (23%) |
| Good | 2,582 (23%) |
| Fair | 1,384 (12%) |
| Poor | 349 (3.1%) |
| (Missing) | 26 (0.2%) |
| **Immigration status** |  |
| Born in this country | 11,270 (99%) |
| Born in another country | 117 (1.0%) |
| (Missing) | 2 (<0.1%) |
| **Age 12 religious service attendance** |  |
| At least 1/week | 9,189 (81%) |
| 1-3/month | 1,687 (15%) |
| <1/month | 236 (2.1%) |
| Never | 198 (1.7%) |
| (Missing) | 79 (0.7%) |
| **Year of birth** |  |
| 1998-2005; age 18-24 | 2,868 (25%) |
| 1993-1998; age 25-29 | 2,035 (18%) |
| 1983-1993; age 30-39 | 2,564 (23%) |
| 1973-1983; age 40-49 | 1,708 (15%) |
| 1963-1973; age 50-59 | 1,072 (9.4%) |
| 1953-1963; age 60-69 | 710 (6.2%) |
| 1943-1953; age 70-79 | 360 (3.2%) |
| 1943 or earlier; age 80+ | 67 (0.6%) |
| (Missing) | 5 (<0.1%) |
| **Gender** |  |
| Male | 5,567 (49%) |
| Female | 5,813 (51%) |
| Other | 2 (<0.1%) |
| (Missing) | 7 (<0.1%) |
| **Religious affiliation** |  |
| Christianity | 10,369 (91%) |
| Islam | 916 (8.0%) |
| Hinduism | 0 (0%) |
| Buddhism | 5 (<0.1%) |
| Judaism | 6 (<0.1%) |
| Sikhism | 0 (<0.1%) |
| Baha'i | 3 (<0.1%) |
| Jainism | 1 (<0.1%) |
| Shinto | 0 (0%) |
| Taoism | 0 (0%) |
| Confucianism | 0 (0%) |
| Primal, Animist, or Folk religion | 13 (0.1%) |
| Spiritism | 0 (0%) |
| Umbanda, Candomble, and other African-derived religions | 0 (0%) |
| Chinese folk/traditional religion | 0 (0%) |
| Some other religion | 0 (<0.1%) |
| No religion/Atheist/Agnostic | 67 (0.6%) |
| (Missing) | 9 (<0.1%) |
| **Race/Ethnicity** |  |
| Embu | 197 (1.7%) |
| Kalenjin | 1,377 (12%) |
| Kamba | 1,299 (11%) |
| Kenyan Somali/Somali | 396 (3.5%) |
| Kikuyu | 2,119 (19%) |
| Kisii | 789 (6.9%) |
| Luhya | 1,943 (17%) |
| Luo | 1,120 (9.8%) |
| Maasai | 237 (2.1%) |
| Meru | 630 (5.5%) |
| Miji Kenda tribes | 708 (6.2%) |
| Other | 548 (4.8%) |
| (Missing) | 27 (0.2%) |
| ^1^n (%) | |

Table S11b. Childhood predictors regression for Kenya

| Variable | Category | Risk-Ratio | RR 95% CI | Global p-value |
| --- | --- | --- | --- | --- |
| Relationship with mother | (Ref: Very bad/somewhat bad) |  |  | 0.422 |
|  | Very good/somewhat good | 0.89 | (0.67,1.19) |  |
| Relationship with father | (Ref: Very bad/somewhat bad) |  |  | 0.803 |
|  | Very good/somewhat good | 1.02 | (0.82,1.29) |  |
| Parent marital status | (Ref: Parents married) |  |  | 0.002 |
|  | Divorced | 1.08 | (0.87,1.35) |  |
|  | Parents were never married | 1.43 | (1.17,1.75) |  |
|  | One or both parents had died | 1.32 | (1.01,1.71) |  |
| Subjective financial status of family growing up | (Ref: Got by) |  |  | 0.467 |
|  | Lived comfortably | 0.97 | (0.83,1.13) |  |
|  | Found it difficult | 1.03 | (0.89,1.18) |  |
|  | Found it very difficult | 1.14 | (0.93,1.38) |  |
| Abuse | (Ref: No) |  |  | <.001 |
|  | Yes | 2.19 | (1.91,2.50) |  |
| Outsider growing up | (Ref: No) |  |  | 0.221 |
|  | Yes | 1.11 | (0.94,1.31) |  |
| Self-rated health growing up | (Ref: Good) |  |  | 0.020 |
|  | Excellent | 0.93 | (0.79,1.09) |  |
|  | Very good | 0.88 | (0.74,1.04) |  |
|  | Fair | 1.14 | (0.93,1.40) |  |
|  | Poor | 1.38 | (1.02,1.87) |  |
| Immigration status | (Ref: Born in this country) |  |  | 0.596 |
|  | Born in another country | 1.14 | (0.70,1.86) |  |
| Age 12 religious service attendance | (Ref: Never) |  |  | 0.115 |
|  | At least 1/week | 1.54 | (0.98,2.44) |  |
|  | 1-3/month | 1.55 | (0.97,2.47) |  |
|  | < 1/month | 1.94 | (1.09,3.44) |  |
| Year of birth | (Ref: 1998-2005; current age: 18-24) |  |  | <.001 |
|  | 1993-1998; age 25-29 | 1.05 | (0.87,1.27) |  |
|  | 1983-1993; age 30-39 | 1.37 | (1.16,1.63) |  |
|  | 1973-1983; age 40-49 | 1.99 | (1.65,2.41) |  |
|  | 1963-1973; age 50-59 | 2.85 | (2.33,3.50) |  |
|  | 1953-1963; age 60-69 | 3.80 | (2.99,4.82) |  |
|  | 1943-1953; age 70-79 | 5.56 | (4.40,7.02) |  |
|  | 1943 or earlier; age 80+ | 6.64 | (3.83,11.52) |  |
| Gender | (Ref: Male) |  |  | <.001 |
|  | Female | 1.37 | (1.22,1.55) |  |
|  | Other | 0.00 | (0.00,0.00) |  |
| Religious affiliation | (Ref: Christianity) |  |  | 0.225 |
|  | Islam | 1.22 | (0.97,1.54) |  |
|  | Collapsed affiliations with prevalence<3% | 0.91 | (0.46,1.81) |  |
| Race/ethnicity | (Ref: Plurality group) |  |  | 0.003 |
|  | Non-plurality groups | 1.28 | (1.09,1.50) |  |

Table S11c. Sensitivity to unmeasured confounding of childhood predictors in Kenya

| Variable | Category | E-value for Estimate | E-value for 95% CI |
| --- | --- | --- | --- |
| Relationship with mother | (Ref: Very bad/somewhat bad) |  |  |
|  | Very good/somewhat good | 1.49 | 1.00 |
| Relationship with father | (Ref: Very bad/somewhat bad) |  |  |
|  | Very good/somewhat good | 1.18 | 1.00 |
| Parent marital status | (Ref: Parents married) |  |  |
|  | Divorced | 1.38 | 1.00 |
|  | Parents were never married | 2.21 | 1.62 |
|  | One or both parents had died | 1.96 | 1.13 |
| Subjective financial status of family growing up | (Ref: Got by) |  |  |
|  | Lived comfortably | 1.23 | 1.00 |
|  | Found it difficult | 1.19 | 1.00 |
|  | Found it very difficult | 1.53 | 1.00 |
| Abuse | (Ref: No) |  |  |
|  | Yes | 3.80 | 3.24 |
| Outsider growing up | (Ref: No) |  |  |
|  | Yes | 1.46 | 1.00 |
| Self-rated health growing up | (Ref: Good) |  |  |
|  | Excellent | 1.36 | 1.00 |
|  | Very good | 1.52 | 1.00 |
|  | Fair | 1.54 | 1.00 |
|  | Poor | 2.11 | 1.17 |
| Immigration status | (Ref: Born in this country) |  |  |
|  | Born in another country | 1.54 | 1.00 |
| Age 12 religious service attendance | (Ref: Never) |  |  |
|  | At least 1/week | 2.46 | 1.00 |
|  | 1-3/month | 2.46 | 1.00 |
|  | < 1/month | 3.29 | 1.42 |
| Year of birth | (Ref: 1998-2005; current age: 18-24) |  |  |
|  | 1993-1998; age 25-29 | 1.29 | 1.00 |
|  | 1983-1993; age 30-39 | 2.09 | 1.59 |
|  | 1973-1983; age 40-49 | 3.40 | 2.68 |
|  | 1963-1973; age 50-59 | 5.15 | 4.08 |
|  | 1953-1963; age 60-69 | 7.06 | 5.44 |
|  | 1943-1953; age 70-79 | 10.59 | 8.27 |
|  | 1943 or earlier; age 80+ | 12.76 | 7.12 |
| Gender | (Ref: Male) |  |  |
|  | Female | 2.09 | 1.73 |
|  | Other | 35484.31 | 6850.37 |
| Religious affiliation | (Ref: Christianity) |  |  |
|  | Islam | 1.74 | 1.00 |
|  | Collapsed affiliations with prevalence<3% | 1.42 | 1.00 |
| Race/ethnicity | (Ref: Plurality group) |  |  |
|  | Non-plurality groups | 1.88 | 1.41 |

Table S12a. Nationally representative descriptive statistics for Mexico

| **Characteristic** | **N = 5,776**^1^ |
| --- | --- |
| **Relationship with mother** |  |
| Very good | 3,912 (68%) |
| Somewhat good | 1,340 (23%) |
| Somewhat bad | 177 (3.1%) |
| Very bad | 90 (1.6%) |
| Does not apply | 177 (3.1%) |
| (Missing) | 80 (1.4%) |
| **Relationship with father** |  |
| Very good | 3,089 (53%) |
| Somewhat good | 1,556 (27%) |
| Somewhat bad | 335 (5.8%) |
| Very bad | 267 (4.6%) |
| Does not apply | 470 (8.1%) |
| (Missing) | 60 (1.0%) |
| **Parent marital status** |  |
| Parents married | 3,999 (69%) |
| Divorced | 341 (5.9%) |
| Parents were never married | 827 (14%) |
| One or both parents had died | 176 (3.0%) |
| (Missing) | 432 (7.5%) |
| **Subjective financial status of family growing up** |  |
| Lived comfortably | 1,775 (31%) |
| Got by | 1,872 (32%) |
| Found it difficult | 1,712 (30%) |
| Found it very difficult | 369 (6.4%) |
| (Missing) | 48 (0.8%) |
| **Abuse** |  |
| Yes | 905 (16%) |
| No | 4,604 (80%) |
| (Missing) | 267 (4.6%) |
| **Outsider growing up** |  |
| Yes | 772 (13%) |
| No | 4,897 (85%) |
| (Missing) | 107 (1.9%) |
| **Self-rated health growing up** |  |
| Excellent | 1,860 (32%) |
| Very good | 1,350 (23%) |
| Good | 1,677 (29%) |
| Fair | 743 (13%) |
| Poor | 133 (2.3%) |
| (Missing) | 14 (0.2%) |
| **Immigration status** |  |
| Born in this country | 5,517 (96%) |
| Born in another country | 108 (1.9%) |
| (Missing) | 151 (2.6%) |
| **Age 12 religious service attendance** |  |
| At least 1/week | 2,514 (44%) |
| 1-3/month | 1,162 (20%) |
| <1/month | 1,087 (19%) |
| Never | 944 (16%) |
| (Missing) | 69 (1.2%) |
| **Year of birth** |  |
| 1998-2005; age 18-24 | 986 (17%) |
| 1993-1998; age 25-29 | 623 (11%) |
| 1983-1993; age 30-39 | 1,312 (23%) |
| 1973-1983; age 40-49 | 1,027 (18%) |
| 1963-1973; age 50-59 | 873 (15%) |
| 1953-1963; age 60-69 | 611 (11%) |
| 1943-1953; age 70-79 | 277 (4.8%) |
| 1943 or earlier; age 80+ | 68 (1.2%) |
| (Missing) | 0 (0%) |
| **Gender** |  |
| Male | 2,755 (48%) |
| Female | 2,997 (52%) |
| Other | 3 (<0.1%) |
| (Missing) | 21 (0.4%) |
| **Religious affiliation** |  |
| Christianity | 5,337 (92%) |
| Islam | 6 (<0.1%) |
| Hinduism | 1 (<0.1%) |
| Buddhism | 1 (<0.1%) |
| Judaism | 8 (0.1%) |
| Sikhism | 4 (<0.1%) |
| Baha'i | 1 (<0.1%) |
| Jainism | 0 (0%) |
| Shinto | 2 (<0.1%) |
| Taoism | 5 (<0.1%) |
| Confucianism | 0 (0%) |
| Primal, Animist, or Folk religion | 2 (<0.1%) |
| Spiritism | 0 (0%) |
| Umbanda, Candomble, and other African-derived religions | 0 (0%) |
| Chinese folk/traditional religion | 0 (0%) |
| Some other religion | 7 (0.1%) |
| No religion/Atheist/Agnostic | 328 (5.7%) |
| (Missing) | 74 (1.3%) |
| **Race/Ethnicity** |  |
| Black | 108 (1.9%) |
| Indigenous | 594 (10%) |
| Mestizo | 2,762 (48%) |
| Mulatto | 63 (1.1%) |
| Other | 339 (5.9%) |
| White | 1,116 (19%) |
| (Missing) | 794 (14%) |
| ^1^n (%) | |

Table S12b. Childhood predictors regression for Mexico

| Variable | Category | Risk-Ratio | RR 95% CI | Global p-value |
| --- | --- | --- | --- | --- |
| Relationship with mother | (Ref: Very bad/somewhat bad) |  |  | 0.812 |
|  | Very good/somewhat good | 0.98 | (0.75,1.26) |  |
| Relationship with father | (Ref: Very bad/somewhat bad) |  |  | 0.440 |
|  | Very good/somewhat good | 1.09 | (0.86,1.37) |  |
| Parent marital status | (Ref: Parents married) |  |  | 0.628 |
|  | Divorced | 1.09 | (0.84,1.41) |  |
|  | Parents were never married | 0.95 | (0.77,1.19) |  |
|  | One or both parents had died | 0.84 | (0.55,1.29) |  |
| Subjective financial status of family growing up | (Ref: Got by) |  |  | 0.033 |
|  | Lived comfortably | 1.09 | (0.87,1.36) |  |
|  | Found it difficult | 1.26 | (1.04,1.52) |  |
|  | Found it very difficult | 1.43 | (1.10,1.86) |  |
| Abuse | (Ref: No) |  |  | 0.004 |
|  | Yes | 1.27 | (1.08,1.50) |  |
| Outsider growing up | (Ref: No) |  |  | <.001 |
|  | Yes | 1.37 | (1.14,1.64) |  |
| Self-rated health growing up | (Ref: Good) |  |  | <.001 |
|  | Excellent | 0.73 | (0.60,0.89) |  |
|  | Very good | 0.81 | (0.65,1.01) |  |
|  | Fair | 1.24 | (1.03,1.49) |  |
|  | Poor | 1.49 | (1.04,2.15) |  |
| Immigration status | (Ref: Born in this country) |  |  | 0.656 |
|  | Born in another country | 1.13 | (0.66,1.94) |  |
| Age 12 religious service attendance | (Ref: Never) |  |  | 0.223 |
|  | At least 1/week | 1.10 | (0.86,1.40) |  |
|  | 1-3/month | 1.11 | (0.85,1.47) |  |
|  | < 1/month | 0.89 | (0.67,1.19) |  |
| Year of birth | (Ref: 1998-2005; current age: 18-24) |  |  | <.001 |
|  | 1993-1998; age 25-29 | 1.19 | (0.81,1.75) |  |
|  | 1983-1993; age 30-39 | 1.22 | (0.88,1.68) |  |
|  | 1973-1983; age 40-49 | 2.15 | (1.58,2.94) |  |
|  | 1963-1973; age 50-59 | 2.75 | (2.00,3.77) |  |
|  | 1953-1963; age 60-69 | 4.19 | (3.09,5.68) |  |
|  | 1943-1953; age 70-79 | 4.72 | (3.39,6.57) |  |
|  | 1943 or earlier; age 80+ | 4.88 | (3.00,7.95) |  |
| Gender | (Ref: Male) |  |  | 0.012 |
|  | Female | 1.25 | (1.07,1.45) |  |
|  | Other | 1.10 | (0.23,5.26) |  |
| Religious affiliation | (Ref: No religion/Atheist/Agnostic) |  |  | 0.105 |
|  | Christianity | 0.96 | (0.70,1.32) |  |
|  | Collapsed affiliations with prevalence<3% | 1.57 | (0.91,2.72) |  |
| Race/ethnicity | (Ref: Plurality group) |  |  | 0.040 |
|  | Non-plurality groups | 1.16 | (0.98,1.36) |  |

Table S12c. Sensitivity to unmeasured confounding of childhood predictors in Mexico

| Variable | Category | E-value for Estimate | E-value for 95% CI |
| --- | --- | --- | --- |
| Relationship with mother | (Ref: Very bad/somewhat bad) |  |  |
|  | Very good/somewhat good | 1.19 | 1.00 |
| Relationship with father | (Ref: Very bad/somewhat bad) |  |  |
|  | Very good/somewhat good | 1.39 | 1.00 |
| Parent marital status | (Ref: Parents married) |  |  |
|  | Divorced | 1.40 | 1.00 |
|  | Parents were never married | 1.27 | 1.00 |
|  | One or both parents had died | 1.66 | 1.00 |
| Subjective financial status of family growing up | (Ref: Got by) |  |  |
|  | Lived comfortably | 1.41 | 1.00 |
|  | Found it difficult | 1.82 | 1.24 |
|  | Found it very difficult | 2.21 | 1.42 |
| Abuse | (Ref: No) |  |  |
|  | Yes | 1.86 | 1.36 |
| Outsider growing up | (Ref: No) |  |  |
|  | Yes | 2.08 | 1.54 |
| Self-rated health growing up | (Ref: Good) |  |  |
|  | Excellent | 2.09 | 1.50 |
|  | Very good | 1.76 | 1.00 |
|  | Fair | 1.78 | 1.19 |
|  | Poor | 2.35 | 1.23 |
| Immigration status | (Ref: Born in this country) |  |  |
|  | Born in another country | 1.51 | 1.00 |
| Age 12 religious service attendance | (Ref: Never) |  |  |
|  | At least 1/week | 1.43 | 1.00 |
|  | 1-3/month | 1.47 | 1.00 |
|  | < 1/month | 1.50 | 1.00 |
| Year of birth | (Ref: 1998-2005; current age: 18-24) |  |  |
|  | 1993-1998; age 25-29 | 1.66 | 1.00 |
|  | 1983-1993; age 30-39 | 1.73 | 1.00 |
|  | 1973-1983; age 40-49 | 3.73 | 2.53 |
|  | 1963-1973; age 50-59 | 4.94 | 3.41 |
|  | 1953-1963; age 60-69 | 7.84 | 5.62 |
|  | 1943-1953; age 70-79 | 8.91 | 6.23 |
|  | 1943 or earlier; age 80+ | 9.24 | 5.44 |
| Gender | (Ref: Male) |  |  |
|  | Female | 1.80 | 1.35 |
|  | Other | 1.42 | 1.00 |
| Religious affiliation | (Ref: No religion/Atheist/Agnostic) |  |  |
|  | Christianity | 1.23 | 1.00 |
|  | Collapsed affiliations with prevalence<3% | 2.52 | 1.00 |
| Race/ethnicity | (Ref: Plurality group) |  |  |
|  | Non-plurality groups | 1.58 | 1.00 |

Table S13a. Nationally representative descriptive statistics for Nigeria

| **Characteristic** | **N = 6,827**^1^ |
| --- | --- |
| **Relationship with mother** |  |
| Very good | 5,986 (88%) |
| Somewhat good | 648 (9.5%) |
| Somewhat bad | 62 (0.9%) |
| Very bad | 18 (0.3%) |
| Does not apply | 104 (1.5%) |
| (Missing) | 9 (0.1%) |
| **Relationship with father** |  |
| Very good | 5,578 (82%) |
| Somewhat good | 924 (14%) |
| Somewhat bad | 76 (1.1%) |
| Very bad | 43 (0.6%) |
| Does not apply | 177 (2.6%) |
| (Missing) | 29 (0.4%) |
| **Parent marital status** |  |
| Parents married | 5,568 (82%) |
| Divorced | 307 (4.5%) |
| Parents were never married | 335 (4.9%) |
| One or both parents had died | 462 (6.8%) |
| (Missing) | 154 (2.3%) |
| **Subjective financial status of family growing up** |  |
| Lived comfortably | 2,192 (32%) |
| Got by | 2,381 (35%) |
| Found it difficult | 1,661 (24%) |
| Found it very difficult | 563 (8.3%) |
| (Missing) | 29 (0.4%) |
| **Abuse** |  |
| Yes | 880 (13%) |
| No | 5,851 (86%) |
| (Missing) | 96 (1.4%) |
| **Outsider growing up** |  |
| Yes | 669 (9.8%) |
| No | 6,059 (89%) |
| (Missing) | 99 (1.5%) |
| **Self-rated health growing up** |  |
| Excellent | 2,644 (39%) |
| Very good | 2,613 (38%) |
| Good | 1,152 (17%) |
| Fair | 306 (4.5%) |
| Poor | 98 (1.4%) |
| (Missing) | 14 (0.2%) |
| **Immigration status** |  |
| Born in this country | 6,779 (99%) |
| Born in another country | 47 (0.7%) |
| (Missing) | 1 (<0.1%) |
| **Age 12 religious service attendance** |  |
| At least 1/week | 5,907 (87%) |
| 1-3/month | 600 (8.8%) |
| <1/month | 136 (2.0%) |
| Never | 138 (2.0%) |
| (Missing) | 45 (0.7%) |
| **Year of birth** |  |
| 1998-2005; age 18-24 | 1,533 (22%) |
| 1993-1998; age 25-29 | 1,193 (17%) |
| 1983-1993; age 30-39 | 1,943 (28%) |
| 1973-1983; age 40-49 | 1,059 (16%) |
| 1963-1973; age 50-59 | 619 (9.1%) |
| 1953-1963; age 60-69 | 296 (4.3%) |
| 1943-1953; age 70-79 | 133 (2.0%) |
| 1943 or earlier; age 80+ | 50 (0.7%) |
| (Missing) | 0 (0%) |
| **Gender** |  |
| Male | 3,371 (49%) |
| Female | 3,456 (51%) |
| Other | 0 (<0.1%) |
| (Missing) | 0 (0%) |
| **Religious affiliation** |  |
| Christianity | 3,463 (51%) |
| Islam | 3,314 (49%) |
| Hinduism | 0 (0%) |
| Buddhism | 0 (<0.1%) |
| Judaism | 0 (0%) |
| Sikhism | 0 (0%) |
| Baha'i | 0 (0%) |
| Jainism | 0 (0%) |
| Shinto | 0 (0%) |
| Taoism | 0 (0%) |
| Confucianism | 0 (<0.1%) |
| Primal, Animist, or Folk religion | 17 (0.3%) |
| Spiritism | 0 (0%) |
| Umbanda, Candomble, and other African-derived religions | 0 (0%) |
| Chinese folk/traditional religion | 0 (0%) |
| Some other religion | 0 (0%) |
| No religion/Atheist/Agnostic | 19 (0.3%) |
| (Missing) | 14 (0.2%) |
| **Race/Ethnicity** |  |
| Edo | 116 (1.7%) |
| Efik | 48 (0.7%) |
| Fulani | 266 (3.9%) |
| Hausa | 2,342 (34%) |
| Ibibio | 180 (2.6%) |
| Idoma | 61 (0.9%) |
| Igala | 77 (1.1%) |
| Igbo (Ibo) | 1,111 (16%) |
| Ijaw | 110 (1.6%) |
| Kanuri | 31 (0.5%) |
| Other | 1,014 (15%) |
| Tiv | 198 (2.9%) |
| Urhobo | 38 (0.6%) |
| Yoruba | 1,230 (18%) |
| (Missing) | 4 (<0.1%) |
| ^1^n (%) | |

Table S13b. Childhood predictors regression for Nigeria

| Variable | Category | Risk-Ratio | RR 95% CI | Global p-value |
| --- | --- | --- | --- | --- |
| Relationship with mother | (Ref: Very bad/somewhat bad) |  |  | 0.475 |
|  | Very good/somewhat good | 1.31 | (0.59,2.94) |  |
| Relationship with father | (Ref: Very bad/somewhat bad) |  |  | 0.253 |
|  | Very good/somewhat good | 1.39 | (0.79,2.47) |  |
| Parent marital status | (Ref: Parents married) |  |  | 0.062 |
|  | Divorced | 1.18 | (0.83,1.66) |  |
|  | Parents were never married | 1.51 | (1.11,2.05) |  |
|  | One or both parents had died | 1.10 | (0.78,1.54) |  |
| Subjective financial status of family growing up | (Ref: Got by) |  |  | 0.908 |
|  | Lived comfortably | 0.99 | (0.79,1.25) |  |
|  | Found it difficult | 1.04 | (0.82,1.31) |  |
|  | Found it very difficult | 1.11 | (0.81,1.52) |  |
| Abuse | (Ref: No) |  |  | <.001 |
|  | Yes | 1.98 | (1.62,2.42) |  |
| Outsider growing up | (Ref: No) |  |  | 0.107 |
|  | Yes | 1.24 | (0.95,1.61) |  |
| Self-rated health growing up | (Ref: Good) |  |  | 0.243 |
|  | Excellent | 1.22 | (0.93,1.61) |  |
|  | Very good | 1.12 | (0.87,1.45) |  |
|  | Fair | 0.79 | (0.46,1.33) |  |
|  | Poor | 1.44 | (0.79,2.61) |  |
| Immigration status | (Ref: Born in this country) |  |  | 0.033 |
|  | Born in another country | 1.95 | (1.06,3.59) |  |
| Age 12 religious service attendance | (Ref: Never) |  |  | 0.025 |
|  | At least 1/week | 1.03 | (0.45,2.37) |  |
|  | 1-3/month | 1.34 | (0.57,3.13) |  |
|  | < 1/month | 1.69 | (0.68,4.21) |  |
| Year of birth | (Ref: 1998-2005; current age: 18-24) |  |  | <.001 |
|  | 1993-1998; age 25-29 | 1.02 | (0.77,1.33) |  |
|  | 1983-1993; age 30-39 | 0.98 | (0.76,1.26) |  |
|  | 1973-1983; age 40-49 | 1.71 | (1.31,2.23) |  |
|  | 1963-1973; age 50-59 | 2.54 | (1.85,3.48) |  |
|  | 1953-1963; age 60-69 | 3.69 | (2.61,5.22) |  |
|  | 1943-1953; age 70-79 | 3.28 | (2.10,5.13) |  |
|  | 1943 or earlier; age 80+ | 8.00 | (5.39,11.88) |  |
| Gender | (Ref: Male) |  |  | <.001 |
|  | Female | 1.06 | (0.90,1.26) |  |
|  | Other | 0.00 | (0.00,0.00) |  |
| Religious affiliation | (Ref: Christianity) |  |  | 0.074 |
|  | Islam | 0.72 | (0.52,0.99) |  |
|  | Collapsed affiliations with prevalence<3% | 0.56 | (0.17,1.85) |  |
| Race/ethnicity | (Ref: Plurality group) |  |  | 0.784 |
|  | Non-plurality groups | 1.04 | (0.73,1.50) |  |

Table S13c. Sensitivity to unmeasured confounding of childhood predictors in Nigeria

| Variable | Category | E-value for Estimate | E-value for 95% CI |
| --- | --- | --- | --- |
| Relationship with mother | (Ref: Very bad/somewhat bad) |  |  |
|  | Very good/somewhat good | 1.96 | 1.00 |
| Relationship with father | (Ref: Very bad/somewhat bad) |  |  |
|  | Very good/somewhat good | 2.14 | 1.00 |
| Parent marital status | (Ref: Parents married) |  |  |
|  | Divorced | 1.63 | 1.00 |
|  | Parents were never married | 2.38 | 1.46 |
|  | One or both parents had died | 1.42 | 1.00 |
| Subjective financial status of family growing up | (Ref: Got by) |  |  |
|  | Lived comfortably | 1.09 | 1.00 |
|  | Found it difficult | 1.24 | 1.00 |
|  | Found it very difficult | 1.46 | 1.00 |
| Abuse | (Ref: No) |  |  |
|  | Yes | 3.37 | 2.62 |
| Outsider growing up | (Ref: No) |  |  |
|  | Yes | 1.78 | 1.00 |
| Self-rated health growing up | (Ref: Good) |  |  |
|  | Excellent | 1.74 | 1.00 |
|  | Very good | 1.49 | 1.00 |
|  | Fair | 1.86 | 1.00 |
|  | Poor | 2.23 | 1.00 |
| Immigration status | (Ref: Born in this country) |  |  |
|  | Born in another country | 3.31 | 1.31 |
| Age 12 religious service attendance | (Ref: Never) |  |  |
|  | At least 1/week | 1.22 | 1.00 |
|  | 1-3/month | 2.01 | 1.00 |
|  | < 1/month | 2.77 | 1.00 |
| Year of birth | (Ref: 1998-2005; current age: 18-24) |  |  |
|  | 1993-1998; age 25-29 | 1.15 | 1.00 |
|  | 1983-1993; age 30-39 | 1.16 | 1.00 |
|  | 1973-1983; age 40-49 | 2.81 | 1.95 |
|  | 1963-1973; age 50-59 | 4.52 | 3.11 |
|  | 1953-1963; age 60-69 | 6.84 | 4.65 |
|  | 1943-1953; age 70-79 | 6.01 | 3.62 |
|  | 1943 or earlier; age 80+ | 15.49 | 10.26 |
| Gender | (Ref: Male) |  |  |
|  | Female | 1.33 | 1.00 |
|  | Other | 26282.06 | 3634.09 |
| Religious affiliation | (Ref: Christianity) |  |  |
|  | Islam | 2.13 | 1.11 |
|  | Collapsed affiliations with prevalence<3% | 2.97 | 1.00 |
| Race/ethnicity | (Ref: Plurality group) |  |  |
|  | Non-plurality groups | 1.25 | 1.00 |

Table S14a. Nationally representative descriptive statistics for Philippines

| **Characteristic** | **N = 5,292**^1^ |
| --- | --- |
| **Relationship with mother** |  |
| Very good | 3,333 (63%) |
| Somewhat good | 1,703 (32%) |
| Somewhat bad | 124 (2.3%) |
| Very bad | 39 (0.7%) |
| Does not apply | 59 (1.1%) |
| (Missing) | 35 (0.7%) |
| **Relationship with father** |  |
| Very good | 3,443 (65%) |
| Somewhat good | 1,429 (27%) |
| Somewhat bad | 159 (3.0%) |
| Very bad | 58 (1.1%) |
| Does not apply | 108 (2.0%) |
| (Missing) | 95 (1.8%) |
| **Parent marital status** |  |
| Parents married | 4,575 (86%) |
| Divorced | 64 (1.2%) |
| Parents were never married | 517 (9.8%) |
| One or both parents had died | 51 (1.0%) |
| (Missing) | 85 (1.6%) |
| **Subjective financial status of family growing up** |  |
| Lived comfortably | 937 (18%) |
| Got by | 3,006 (57%) |
| Found it difficult | 1,055 (20%) |
| Found it very difficult | 291 (5.5%) |
| (Missing) | 3 (<0.1%) |
| **Abuse** |  |
| Yes | 420 (7.9%) |
| No | 4,837 (91%) |
| (Missing) | 35 (0.7%) |
| **Outsider growing up** |  |
| Yes | 395 (7.5%) |
| No | 4,884 (92%) |
| (Missing) | 13 (0.2%) |
| **Self-rated health growing up** |  |
| Excellent | 1,041 (20%) |
| Very good | 559 (11%) |
| Good | 2,174 (41%) |
| Fair | 1,246 (24%) |
| Poor | 272 (5.1%) |
| (Missing) | 0 (<0.1%) |
| **Immigration status** |  |
| Born in this country | 5,284 (100%) |
| Born in another country | 8 (0.1%) |
| (Missing) | 0 (0%) |
| **Age 12 religious service attendance** |  |
| At least 1/week | 2,453 (46%) |
| 1-3/month | 1,699 (32%) |
| <1/month | 892 (17%) |
| Never | 201 (3.8%) |
| (Missing) | 47 (0.9%) |
| **Year of birth** |  |
| 1998-2005; age 18-24 | 1,073 (20%) |
| 1993-1998; age 25-29 | 695 (13%) |
| 1983-1993; age 30-39 | 1,160 (22%) |
| 1973-1983; age 40-49 | 972 (18%) |
| 1963-1973; age 50-59 | 732 (14%) |
| 1953-1963; age 60-69 | 495 (9.4%) |
| 1943-1953; age 70-79 | 143 (2.7%) |
| 1943 or earlier; age 80+ | 23 (0.4%) |
| (Missing) | 0 (0%) |
| **Gender** |  |
| Male | 2,625 (50%) |
| Female | 2,643 (50%) |
| Other | 13 (0.2%) |
| (Missing) | 11 (0.2%) |
| **Religious affiliation** |  |
| Christianity | 4,968 (94%) |
| Islam | 276 (5.2%) |
| Hinduism | 0 (0%) |
| Buddhism | 1 (<0.1%) |
| Judaism | 0 (0%) |
| Sikhism | 4 (<0.1%) |
| Baha'i | 1 (<0.1%) |
| Jainism | 0 (0%) |
| Shinto | 0 (0%) |
| Taoism | 0 (0%) |
| Confucianism | 0 (0%) |
| Primal, Animist, or Folk religion | 14 (0.3%) |
| Spiritism | 0 (0%) |
| Umbanda, Candomble, and other African-derived religions | 0 (0%) |
| Chinese folk/traditional religion | 0 (0%) |
| Some other religion | 9 (0.2%) |
| No religion/Atheist/Agnostic | 9 (0.2%) |
| (Missing) | 11 (0.2%) |
| **Race/Ethnicity** |  |
| Aeta | 1 (<0.1%) |
| Badjao | 2 (<0.1%) |
| Bicolano/Bikolano | 300 (5.7%) |
| Cebuano | 656 (12%) |
| Chinese-Filipino | 3 (<0.1%) |
| Igorot | 42 (0.8%) |
| Ilocano/Ilokano | 429 (8.1%) |
| Ilonggo/Hiligaynon | 428 (8.1%) |
| Kapampangan | 107 (2.0%) |
| Maguindanaoan | 84 (1.6%) |
| Mangyan | 2 (<0.1%) |
| Maranao | 39 (0.7%) |
| Masbateno | 54 (1.0%) |
| Other | 244 (4.6%) |
| Pangasinense | 107 (2.0%) |
| Tagalog | 1,691 (32%) |
| Tausug | 94 (1.8%) |
| Visayan/Bisaya | 739 (14%) |
| Waray | 216 (4.1%) |
| Zamboangueno | 51 (1.0%) |
| (Missing) | 3 (<0.1%) |
| ^1^n (%) | |

Table S14b. Childhood predictors regression for Philippines

| Variable | Category | Risk-Ratio | RR 95% CI | Global p-value |
| --- | --- | --- | --- | --- |
| Relationship with mother | (Ref: Very bad/somewhat bad) |  |  | 0.228 |
|  | Very good/somewhat good | 0.88 | (0.72,1.08) |  |
| Relationship with father | (Ref: Very bad/somewhat bad) |  |  | 0.429 |
|  | Very good/somewhat good | 0.93 | (0.77,1.12) |  |
| Parent marital status | (Ref: Parents married) |  |  | 0.630 |
|  | Divorced | 0.80 | (0.53,1.21) |  |
|  | Parents were never married | 1.01 | (0.87,1.17) |  |
|  | One or both parents had died | 1.13 | (0.77,1.66) |  |
| Subjective financial status of family growing up | (Ref: Got by) |  |  | 0.047 |
|  | Lived comfortably | 1.12 | (0.97,1.30) |  |
|  | Found it difficult | 1.16 | (1.04,1.29) |  |
|  | Found it very difficult | 1.04 | (0.87,1.25) |  |
| Abuse | (Ref: No) |  |  | <.001 |
|  | Yes | 1.51 | (1.33,1.71) |  |
| Outsider growing up | (Ref: No) |  |  | 0.709 |
|  | Yes | 1.03 | (0.88,1.20) |  |
| Self-rated health growing up | (Ref: Good) |  |  | <.001 |
|  | Excellent | 0.95 | (0.83,1.08) |  |
|  | Very good | 1.06 | (0.91,1.25) |  |
|  | Fair | 1.28 | (1.13,1.43) |  |
|  | Poor | 1.69 | (1.44,1.98) |  |
| Immigration status | (Ref: Born in this country) |  |  | 0.547 |
|  | Born in another country | 0.61 | (0.12,3.04) |  |
| Age 12 religious service attendance | (Ref: Never) |  |  | 0.144 |
|  | At least 1/week | 0.82 | (0.67,1.00) |  |
|  | 1-3/month | 0.88 | (0.71,1.08) |  |
|  | < 1/month | 0.79 | (0.62,1.00) |  |
| Year of birth | (Ref: 1998-2005; current age: 18-24) |  |  | <.001 |
|  | 1993-1998; age 25-29 | 0.93 | (0.75,1.17) |  |
|  | 1983-1993; age 30-39 | 1.15 | (0.96,1.37) |  |
|  | 1973-1983; age 40-49 | 1.43 | (1.21,1.68) |  |
|  | 1963-1973; age 50-59 | 1.59 | (1.34,1.89) |  |
|  | 1953-1963; age 60-69 | 1.91 | (1.58,2.32) |  |
|  | 1943-1953; age 70-79 | 1.98 | (1.54,2.54) |  |
|  | 1943 or earlier; age 80+ | 2.27 | (1.39,3.72) |  |
| Gender | (Ref: Male) |  |  | 0.052 |
|  | Female | 0.95 | (0.87,1.04) |  |
|  | Other | 1.61 | (1.00,2.59) |  |
| Religious affiliation | (Ref: Christianity) |  |  | 0.001 |
|  | Islam | 1.20 | (1.04,1.39) |  |
|  | Collapsed affiliations with prevalence<3% | 1.61 | (1.15,2.25) |  |
| Race/ethnicity | (Ref: Plurality group) |  |  | 0.003 |
|  | Non-plurality groups | 1.19 | (1.06,1.33) |  |

Table S14c. Sensitivity to unmeasured confounding of childhood predictors in Philippines

| Variable | Category | E-value for Estimate | E-value for 95% CI |
| --- | --- | --- | --- |
| Relationship with mother | (Ref: Very bad/somewhat bad) |  |  |
|  | Very good/somewhat good | 1.52 | 1.00 |
| Relationship with father | (Ref: Very bad/somewhat bad) |  |  |
|  | Very good/somewhat good | 1.37 | 1.00 |
| Parent marital status | (Ref: Parents married) |  |  |
|  | Divorced | 1.80 | 1.00 |
|  | Parents were never married | 1.13 | 1.00 |
|  | One or both parents had died | 1.52 | 1.00 |
| Subjective financial status of family growing up | (Ref: Got by) |  |  |
|  | Lived comfortably | 1.49 | 1.00 |
|  | Found it difficult | 1.59 | 1.26 |
|  | Found it very difficult | 1.25 | 1.00 |
| Abuse | (Ref: No) |  |  |
|  | Yes | 2.39 | 2.00 |
| Outsider growing up | (Ref: No) |  |  |
|  | Yes | 1.21 | 1.00 |
| Self-rated health growing up | (Ref: Good) |  |  |
|  | Excellent | 1.29 | 1.00 |
|  | Very good | 1.32 | 1.00 |
|  | Fair | 1.87 | 1.52 |
|  | Poor | 2.77 | 2.24 |
| Immigration status | (Ref: Born in this country) |  |  |
|  | Born in another country | 2.66 | 1.00 |
| Age 12 religious service attendance | (Ref: Never) |  |  |
|  | At least 1/week | 1.74 | 1.00 |
|  | 1-3/month | 1.54 | 1.00 |
|  | < 1/month | 1.85 | 1.00 |
| Year of birth | (Ref: 1998-2005; current age: 18-24) |  |  |
|  | 1993-1998; age 25-29 | 1.35 | 1.00 |
|  | 1983-1993; age 30-39 | 1.56 | 1.00 |
|  | 1973-1983; age 40-49 | 2.21 | 1.72 |
|  | 1963-1973; age 50-59 | 2.56 | 2.00 |
|  | 1953-1963; age 60-69 | 3.24 | 2.54 |
|  | 1943-1953; age 70-79 | 3.37 | 2.45 |
|  | 1943 or earlier; age 80+ | 3.97 | 2.13 |
| Gender | (Ref: Male) |  |  |
|  | Female | 1.27 | 1.00 |
|  | Other | 2.60 | 1.05 |
| Religious affiliation | (Ref: Christianity) |  |  |
|  | Islam | 1.70 | 1.24 |
|  | Collapsed affiliations with prevalence<3% | 2.60 | 1.57 |
| Race/ethnicity | (Ref: Plurality group) |  |  |
|  | Non-plurality groups | 1.66 | 1.31 |

Table S15a. Nationally representative descriptive statistics for Poland

| **Characteristic** | **N = 10,389**^1^ |
| --- | --- |
| **Relationship with mother** |  |
| Very good | 4,879 (47%) |
| Somewhat good | 4,973 (48%) |
| Somewhat bad | 285 (2.7%) |
| Very bad | 58 (0.6%) |
| Does not apply | 80 (0.8%) |
| (Missing) | 112 (1.1%) |
| **Relationship with father** |  |
| Very good | 4,231 (41%) |
| Somewhat good | 4,984 (48%) |
| Somewhat bad | 516 (5.0%) |
| Very bad | 78 (0.7%) |
| Does not apply | 407 (3.9%) |
| (Missing) | 173 (1.7%) |
| **Parent marital status** |  |
| Parents married | 8,972 (86%) |
| Divorced | 587 (5.7%) |
| Parents were never married | 193 (1.9%) |
| One or both parents had died | 313 (3.0%) |
| (Missing) | 324 (3.1%) |
| **Subjective financial status of family growing up** |  |
| Lived comfortably | 1,384 (13%) |
| Got by | 6,257 (60%) |
| Found it difficult | 2,133 (21%) |
| Found it very difficult | 509 (4.9%) |
| (Missing) | 106 (1.0%) |
| **Abuse** |  |
| Yes | 325 (3.1%) |
| No | 10,009 (96%) |
| (Missing) | 55 (0.5%) |
| **Outsider growing up** |  |
| Yes | 490 (4.7%) |
| No | 9,615 (93%) |
| (Missing) | 284 (2.7%) |
| **Self-rated health growing up** |  |
| Excellent | 2,676 (26%) |
| Very good | 5,371 (52%) |
| Good | 1,779 (17%) |
| Fair | 406 (3.9%) |
| Poor | 123 (1.2%) |
| (Missing) | 34 (0.3%) |
| **Immigration status** |  |
| Born in this country | 10,258 (99%) |
| Born in another country | 108 (1.0%) |
| (Missing) | 23 (0.2%) |
| **Age 12 religious service attendance** |  |
| At least 1/week | 4,751 (46%) |
| 1-3/month | 2,689 (26%) |
| <1/month | 2,161 (21%) |
| Never | 354 (3.4%) |
| (Missing) | 434 (4.2%) |
| **Year of birth** |  |
| 1998-2005; age 18-24 | 955 (9.2%) |
| 1993-1998; age 25-29 | 761 (7.3%) |
| 1983-1993; age 30-39 | 2,159 (21%) |
| 1973-1983; age 40-49 | 1,956 (19%) |
| 1963-1973; age 50-59 | 1,670 (16%) |
| 1953-1963; age 60-69 | 1,909 (18%) |
| 1943-1953; age 70-79 | 833 (8.0%) |
| 1943 or earlier; age 80+ | 145 (1.4%) |
| (Missing) | 1 (<0.1%) |
| **Gender** |  |
| Male | 4,974 (48%) |
| Female | 5,387 (52%) |
| Other | 3 (<0.1%) |
| (Missing) | 26 (0.2%) |
| **Religious affiliation** |  |
| Christianity | 9,861 (95%) |
| Islam | 3 (<0.1%) |
| Hinduism | 0 (0%) |
| Buddhism | 2 (<0.1%) |
| Judaism | 0 (0%) |
| Sikhism | 1 (<0.1%) |
| Baha'i | 0 (0%) |
| Jainism | 0 (0%) |
| Shinto | 0 (0%) |
| Taoism | 0 (0%) |
| Confucianism | 0 (0%) |
| Primal, Animist, or Folk religion | 5 (<0.1%) |
| Spiritism | 0 (0%) |
| Umbanda, Candomble, and other African-derived religions | 0 (0%) |
| Chinese folk/traditional religion | 0 (0%) |
| Some other religion | 0 (0%) |
| No religion/Atheist/Agnostic | 482 (4.6%) |
| (Missing) | 35 (0.3%) |
| **Race/Ethnicity** |  |
| Belarussian | 2 (<0.1%) |
| German | 4 (<0.1%) |
| Kashubians | 3 (<0.1%) |
| Other | 4 (<0.1%) |
| Polish | 10,309 (99%) |
| Silesia | 14 (0.1%) |
| Ukrainian | 38 (0.4%) |
| (Missing) | 14 (0.1%) |
| ^1^n (%) | |

Table S15b. Childhood predictors regression for Poland

| Variable | Category | Risk-Ratio | RR 95% CI | Global p-value |
| --- | --- | --- | --- | --- |
| Relationship with mother | (Ref: Very bad/somewhat bad) |  |  | 0.717 |
|  | Very good/somewhat good | 0.94 | (0.58,1.50) |  |
| Relationship with father | (Ref: Very bad/somewhat bad) |  |  | 0.528 |
|  | Very good/somewhat good | 0.90 | (0.61,1.31) |  |
| Parent marital status | (Ref: Parents married) |  |  | <.001 |
|  | Divorced | 1.36 | (0.97,1.91) |  |
|  | Parents were never married | 1.59 | (0.85,2.99) |  |
|  | One or both parents had died | 1.99 | (1.43,2.77) |  |
| Subjective financial status of family growing up | (Ref: Got by) |  |  | 0.005 |
|  | Lived comfortably | 1.01 | (0.75,1.35) |  |
|  | Found it difficult | 1.23 | (1.02,1.49) |  |
|  | Found it very difficult | 1.66 | (1.24,2.22) |  |
| Abuse | (Ref: No) |  |  | <.001 |
|  | Yes | 1.79 | (1.33,2.40) |  |
| Outsider growing up | (Ref: No) |  |  | 0.549 |
|  | Yes | 0.93 | (0.65,1.34) |  |
| Self-rated health growing up | (Ref: Good) |  |  | <.001 |
|  | Excellent | 0.74 | (0.55,0.99) |  |
|  | Very good | 0.77 | (0.63,0.93) |  |
|  | Fair | 1.49 | (1.08,2.06) |  |
|  | Poor | 1.06 | (0.66,1.70) |  |
| Immigration status | (Ref: Born in this country) |  |  | 0.220 |
|  | Born in another country | 1.39 | (0.79,2.43) |  |
| Age 12 religious service attendance | (Ref: Never) |  |  | 0.436 |
|  | At least 1/week | 0.80 | (0.53,1.20) |  |
|  | 1-3/month | 0.90 | (0.59,1.36) |  |
|  | < 1/month | 0.79 | (0.51,1.22) |  |
| Year of birth | (Ref: 1998-2005; current age: 18-24) |  |  | <.001 |
|  | 1993-1998; age 25-29 | 1.39 | (0.80,2.43) |  |
|  | 1983-1993; age 30-39 | 1.61 | (0.97,2.65) |  |
|  | 1973-1983; age 40-49 | 2.19 | (1.32,3.63) |  |
|  | 1963-1973; age 50-59 | 4.27 | (2.62,6.95) |  |
|  | 1953-1963; age 60-69 | 8.62 | (5.33,13.96) |  |
|  | 1943-1953; age 70-79 | 10.80 | (6.56,17.75) |  |
|  | 1943 or earlier; age 80+ | 11.49 | (6.18,21.34) |  |
| Gender | (Ref: Male) |  |  | <.001 |
|  | Female | 1.03 | (0.88,1.19) |  |
|  | Other | 0.00 | (0.00,1639.80) |  |
| Religious affiliation | (Ref: No religion/Atheist/Agnostic) |  |  | 0.390 |
|  | Christianity | 0.86 | (0.59,1.24) |  |
|  | Collapsed affiliations with prevalence<3% | 1.62 | (0.55,4.78) |  |
| Race/ethnicity | (Ref: Plurality group) |  |  | 0.176 |
|  | Non-plurality groups | 0.40 | (0.11,1.51) |  |

Table S15c. Sensitivity to unmeasured confounding of childhood predictors in Poland

| Variable | Category | E-value for Estimate | E-value for 95% CI |
| --- | --- | --- | --- |
| Relationship with mother | (Ref: Very bad/somewhat bad) |  |  |
|  | Very good/somewhat good | 1.33 | 1.00 |
| Relationship with father | (Ref: Very bad/somewhat bad) |  |  |
|  | Very good/somewhat good | 1.48 | 1.00 |
| Parent marital status | (Ref: Parents married) |  |  |
|  | Divorced | 2.07 | 1.00 |
|  | Parents were never married | 2.56 | 1.00 |
|  | One or both parents had died | 3.39 | 2.21 |
| Subjective financial status of family growing up | (Ref: Got by) |  |  |
|  | Lived comfortably | 1.11 | 1.00 |
|  | Found it difficult | 1.77 | 1.16 |
|  | Found it very difficult | 2.71 | 1.79 |
| Abuse | (Ref: No) |  |  |
|  | Yes | 2.98 | 2.00 |
| Outsider growing up | (Ref: No) |  |  |
|  | Yes | 1.35 | 1.00 |
| Self-rated health growing up | (Ref: Good) |  |  |
|  | Excellent | 2.04 | 1.11 |
|  | Very good | 1.93 | 1.36 |
|  | Fair | 2.35 | 1.37 |
|  | Poor | 1.31 | 1.00 |
| Immigration status | (Ref: Born in this country) |  |  |
|  | Born in another country | 2.12 | 1.00 |
| Age 12 religious service attendance | (Ref: Never) |  |  |
|  | At least 1/week | 1.82 | 1.00 |
|  | 1-3/month | 1.47 | 1.00 |
|  | < 1/month | 1.85 | 1.00 |
| Year of birth | (Ref: 1998-2005; current age: 18-24) |  |  |
|  | 1993-1998; age 25-29 | 2.13 | 1.00 |
|  | 1983-1993; age 30-39 | 2.60 | 1.00 |
|  | 1973-1983; age 40-49 | 3.80 | 1.97 |
|  | 1963-1973; age 50-59 | 8.00 | 4.68 |
|  | 1953-1963; age 60-69 | 16.73 | 10.13 |
|  | 1943-1953; age 70-79 | 21.08 | 12.61 |
|  | 1943 or earlier; age 80+ | 22.46 | 11.84 |
| Gender | (Ref: Male) |  |  |
|  | Female | 1.20 | 1.00 |
|  | Other | 5108.34 | 1.00 |
| Religious affiliation | (Ref: No religion/Atheist/Agnostic) |  |  |
|  | Christianity | 1.61 | 1.00 |
|  | Collapsed affiliations with prevalence<3% | 2.61 | 1.00 |
| Race/ethnicity | (Ref: Plurality group) |  |  |
|  | Non-plurality groups | 4.42 | 1.00 |

Table S16a. Nationally representative descriptive statistics for South Africa

| **Characteristic** | **N = 2,651**^1^ |
| --- | --- |
| **Relationship with mother** |  |
| Very good | 2,186 (82%) |
| Somewhat good | 263 (9.9%) |
| Somewhat bad | 51 (1.9%) |
| Very bad | 39 (1.5%) |
| Does not apply | 90 (3.4%) |
| (Missing) | 21 (0.8%) |
| **Relationship with father** |  |
| Very good | 1,656 (62%) |
| Somewhat good | 333 (13%) |
| Somewhat bad | 86 (3.3%) |
| Very bad | 159 (6.0%) |
| Does not apply | 331 (12%) |
| (Missing) | 85 (3.2%) |
| **Parent marital status** |  |
| Parents married | 1,321 (50%) |
| Divorced | 131 (5.0%) |
| Parents were never married | 904 (34%) |
| One or both parents had died | 140 (5.3%) |
| (Missing) | 155 (5.8%) |
| **Subjective financial status of family growing up** |  |
| Lived comfortably | 1,050 (40%) |
| Got by | 875 (33%) |
| Found it difficult | 432 (16%) |
| Found it very difficult | 289 (11%) |
| (Missing) | 5 (0.2%) |
| **Abuse** |  |
| Yes | 450 (17%) |
| No | 2,149 (81%) |
| (Missing) | 52 (2.0%) |
| **Outsider growing up** |  |
| Yes | 434 (16%) |
| No | 2,211 (83%) |
| (Missing) | 6 (0.2%) |
| **Self-rated health growing up** |  |
| Excellent | 1,225 (46%) |
| Very good | 590 (22%) |
| Good | 370 (14%) |
| Fair | 266 (10%) |
| Poor | 183 (6.9%) |
| (Missing) | 17 (0.6%) |
| **Immigration status** |  |
| Born in this country | 2,511 (95%) |
| Born in another country | 139 (5.2%) |
| (Missing) | 1 (<0.1%) |
| **Age 12 religious service attendance** |  |
| At least 1/week | 1,681 (63%) |
| 1-3/month | 552 (21%) |
| <1/month | 175 (6.6%) |
| Never | 217 (8.2%) |
| (Missing) | 26 (1.0%) |
| **Year of birth** |  |
| 1998-2005; age 18-24 | 461 (17%) |
| 1993-1998; age 25-29 | 364 (14%) |
| 1983-1993; age 30-39 | 655 (25%) |
| 1973-1983; age 40-49 | 522 (20%) |
| 1963-1973; age 50-59 | 309 (12%) |
| 1953-1963; age 60-69 | 195 (7.4%) |
| 1943-1953; age 70-79 | 120 (4.5%) |
| 1943 or earlier; age 80+ | 17 (0.6%) |
| (Missing) | 9 (0.3%) |
| **Gender** |  |
| Male | 1,288 (49%) |
| Female | 1,356 (51%) |
| Other | 2 (<0.1%) |
| (Missing) | 4 (0.2%) |
| **Religious affiliation** |  |
| Christianity | 2,323 (88%) |
| Islam | 52 (2.0%) |
| Hinduism | 2 (<0.1%) |
| Buddhism | 11 (0.4%) |
| Judaism | 0 (0%) |
| Sikhism | 0 (0%) |
| Baha'i | 0 (0%) |
| Jainism | 0 (0%) |
| Shinto | 2 (<0.1%) |
| Taoism | 1 (<0.1%) |
| Confucianism | 0 (0%) |
| Primal, Animist, or Folk religion | 117 (4.4%) |
| Spiritism | 0 (0%) |
| Umbanda, Candomble, and other African-derived religions | 0 (0%) |
| Chinese folk/traditional religion | 0 (0%) |
| Some other religion | 7 (0.3%) |
| No religion/Atheist/Agnostic | 107 (4.1%) |
| (Missing) | 27 (1.0%) |
| **Race/Ethnicity** |  |
| Asian/Indian | 6 (0.2%) |
| Black | 2,381 (90%) |
| Colored | 252 (9.5%) |
| Other | 1 (<0.1%) |
| White | 8 (0.3%) |
| (Missing) | 3 (0.1%) |
| ^1^n (%) | |

Table S16b. Childhood predictors regression for South Africa

| Variable | Category | Risk-Ratio | RR 95% CI | Global p-value |
| --- | --- | --- | --- | --- |
| Relationship with mother | (Ref: Very bad/somewhat bad) |  |  | 0.751 |
|  | Very good/somewhat good | 1.07 | (0.70,1.64) |  |
| Relationship with father | (Ref: Very bad/somewhat bad) |  |  | 0.826 |
|  | Very good/somewhat good | 0.99 | (0.75,1.30) |  |
| Parent marital status | (Ref: Parents married) |  |  | 0.014 |
|  | Divorced | 1.12 | (0.70,1.79) |  |
|  | Parents were never married | 1.21 | (0.97,1.50) |  |
|  | One or both parents had died | 1.71 | (1.21,2.40) |  |
| Subjective financial status of family growing up | (Ref: Got by) |  |  | 0.705 |
|  | Lived comfortably | 1.02 | (0.79,1.33) |  |
|  | Found it difficult | 0.86 | (0.63,1.19) |  |
|  | Found it very difficult | 0.92 | (0.64,1.32) |  |
| Abuse | (Ref: No) |  |  | <.001 |
|  | Yes | 1.81 | (1.45,2.25) |  |
| Outsider growing up | (Ref: No) |  |  | 0.054 |
|  | Yes | 1.27 | (0.99,1.63) |  |
| Self-rated health growing up | (Ref: Good) |  |  | 0.166 |
|  | Excellent | 0.86 | (0.65,1.14) |  |
|  | Very good | 0.76 | (0.57,1.02) |  |
|  | Fair | 1.01 | (0.72,1.42) |  |
|  | Poor | 1.17 | (0.82,1.68) |  |
| Immigration status | (Ref: Born in this country) |  |  | 0.549 |
|  | Born in another country | 0.85 | (0.49,1.45) |  |
| Age 12 religious service attendance | (Ref: Never) |  |  | 0.146 |
|  | At least 1/week | 1.52 | (0.80,2.89) |  |
|  | 1-3/month | 1.27 | (0.65,2.49) |  |
|  | < 1/month | 1.00 | (0.49,2.04) |  |
| Year of birth | (Ref: 1998-2005; current age: 18-24) |  |  | <.001 |
|  | 1993-1998; age 25-29 | 1.09 | (0.75,1.59) |  |
|  | 1983-1993; age 30-39 | 1.17 | (0.82,1.66) |  |
|  | 1973-1983; age 40-49 | 1.62 | (1.12,2.34) |  |
|  | 1963-1973; age 50-59 | 2.06 | (1.41,3.01) |  |
|  | 1953-1963; age 60-69 | 3.30 | (2.21,4.93) |  |
|  | 1943-1953; age 70-79 | 2.62 | (1.58,4.35) |  |
|  | 1943 or earlier; age 80+ | 4.24 | (1.85,9.69) |  |
| Gender | (Ref: Male) |  |  | 0.038 |
|  | Female | 1.21 | (1.01,1.45) |  |
|  | Other | 2.40 | (0.51,11.34) |  |
| Religious affiliation | (Ref: No religion/Atheist/Agnostic) |  |  | 0.115 |
|  | Primal, Animist, or Folk religion | 0.95 | (0.34,2.71) |  |
|  | Christianity | 1.22 | (0.50,2.95) |  |
|  | Collapsed affiliations with prevalence<3% | 2.07 | (0.78,5.49) |  |
| Race/ethnicity | (Ref: Plurality group) |  |  | 0.858 |
|  | Non-plurality groups | 0.99 | (0.69,1.42) |  |

Table S16c. Sensitivity to unmeasured confounding of childhood predictors in South Africa

| Variable | Category | E-value for Estimate | E-value for 95% CI |
| --- | --- | --- | --- |
| Relationship with mother | (Ref: Very bad/somewhat bad) |  |  |
|  | Very good/somewhat good | 1.34 | 1.00 |
| Relationship with father | (Ref: Very bad/somewhat bad) |  |  |
|  | Very good/somewhat good | 1.14 | 1.00 |
| Parent marital status | (Ref: Parents married) |  |  |
|  | Divorced | 1.49 | 1.00 |
|  | Parents were never married | 1.71 | 1.00 |
|  | One or both parents had died | 2.81 | 1.72 |
| Subjective financial status of family growing up | (Ref: Got by) |  |  |
|  | Lived comfortably | 1.18 | 1.00 |
|  | Found it difficult | 1.58 | 1.00 |
|  | Found it very difficult | 1.40 | 1.00 |
| Abuse | (Ref: No) |  |  |
|  | Yes | 3.02 | 2.27 |
| Outsider growing up | (Ref: No) |  |  |
|  | Yes | 1.86 | 1.00 |
| Self-rated health growing up | (Ref: Good) |  |  |
|  | Excellent | 1.60 | 1.00 |
|  | Very good | 1.96 | 1.00 |
|  | Fair | 1.12 | 1.00 |
|  | Poor | 1.62 | 1.00 |
| Immigration status | (Ref: Born in this country) |  |  |
|  | Born in another country | 1.64 | 1.00 |
| Age 12 religious service attendance | (Ref: Never) |  |  |
|  | At least 1/week | 2.41 | 1.00 |
|  | 1-3/month | 1.86 | 1.00 |
|  | < 1/month | 1.05 | 1.00 |
| Year of birth | (Ref: 1998-2005; current age: 18-24) |  |  |
|  | 1993-1998; age 25-29 | 1.42 | 1.00 |
|  | 1983-1993; age 30-39 | 1.61 | 1.00 |
|  | 1973-1983; age 40-49 | 2.61 | 1.48 |
|  | 1963-1973; age 50-59 | 3.54 | 2.18 |
|  | 1953-1963; age 60-69 | 6.06 | 3.85 |
|  | 1943-1953; age 70-79 | 4.69 | 2.54 |
|  | 1943 or earlier; age 80+ | 7.94 | 3.11 |
| Gender | (Ref: Male) |  |  |
|  | Female | 1.71 | 1.12 |
|  | Other | 4.24 | 1.00 |
| Religious affiliation | (Ref: No religion/Atheist/Agnostic) |  |  |
|  | Primal, Animist, or Folk religion | 1.27 | 1.00 |
|  | Christianity | 1.74 | 1.00 |
|  | Collapsed affiliations with prevalence<3% | 3.55 | 1.00 |
| Race/ethnicity | (Ref: Plurality group) |  |  |
|  | Non-plurality groups | 1.12 | 1.00 |

Table S17a. Nationally representative descriptive statistics for Spain

| **Characteristic** | **N = 6,290**^1^ |
| --- | --- |
| **Relationship with mother** |  |
| Very good | 4,557 (72%) |
| Somewhat good | 1,258 (20%) |
| Somewhat bad | 248 (3.9%) |
| Very bad | 92 (1.5%) |
| Does not apply | 107 (1.7%) |
| (Missing) | 28 (0.4%) |
| **Relationship with father** |  |
| Very good | 4,131 (66%) |
| Somewhat good | 1,397 (22%) |
| Somewhat bad | 309 (4.9%) |
| Very bad | 178 (2.8%) |
| Does not apply | 243 (3.9%) |
| (Missing) | 33 (0.5%) |
| **Parent marital status** |  |
| Parents married | 5,285 (84%) |
| Divorced | 378 (6.0%) |
| Parents were never married | 312 (5.0%) |
| One or both parents had died | 126 (2.0%) |
| (Missing) | 188 (3.0%) |
| **Subjective financial status of family growing up** |  |
| Lived comfortably | 2,041 (32%) |
| Got by | 2,956 (47%) |
| Found it difficult | 1,154 (18%) |
| Found it very difficult | 110 (1.7%) |
| (Missing) | 29 (0.5%) |
| **Abuse** |  |
| Yes | 659 (10%) |
| No | 5,510 (88%) |
| (Missing) | 122 (1.9%) |
| **Outsider growing up** |  |
| Yes | 579 (9.2%) |
| No | 5,637 (90%) |
| (Missing) | 75 (1.2%) |
| **Self-rated health growing up** |  |
| Excellent | 2,450 (39%) |
| Very good | 2,286 (36%) |
| Good | 1,235 (20%) |
| Fair | 164 (2.6%) |
| Poor | 135 (2.1%) |
| (Missing) | 20 (0.3%) |
| **Immigration status** |  |
| Born in this country | 5,479 (87%) |
| Born in another country | 788 (13%) |
| (Missing) | 23 (0.4%) |
| **Age 12 religious service attendance** |  |
| At least 1/week | 2,391 (38%) |
| 1-3/month | 1,132 (18%) |
| <1/month | 1,287 (20%) |
| Never | 1,445 (23%) |
| (Missing) | 36 (0.6%) |
| **Year of birth** |  |
| 1998-2005; age 18-24 | 594 (9.4%) |
| 1993-1998; age 25-29 | 450 (7.2%) |
| 1983-1993; age 30-39 | 1,111 (18%) |
| 1973-1983; age 40-49 | 1,396 (22%) |
| 1963-1973; age 50-59 | 1,252 (20%) |
| 1953-1963; age 60-69 | 977 (16%) |
| 1943-1953; age 70-79 | 467 (7.4%) |
| 1943 or earlier; age 80+ | 43 (0.7%) |
| (Missing) | 0 (0%) |
| **Gender** |  |
| Male | 3,142 (50%) |
| Female | 3,119 (50%) |
| Other | 6 (0.1%) |
| (Missing) | 22 (0.4%) |
| **Religious affiliation** |  |
| Christianity | 5,119 (81%) |
| Islam | 132 (2.1%) |
| Hinduism | 5 (<0.1%) |
| Buddhism | 8 (0.1%) |
| Judaism | 5 (<0.1%) |
| Sikhism | 2 (<0.1%) |
| Baha'i | 0 (0%) |
| Jainism | 0 (0%) |
| Shinto | 0 (0%) |
| Taoism | 0 (0%) |
| Confucianism | 1 (<0.1%) |
| Primal, Animist, or Folk religion | 4 (<0.1%) |
| Spiritism | 0 (0%) |
| Umbanda, Candomble, and other African-derived religions | 0 (0%) |
| Chinese folk/traditional religion | 0 (0%) |
| Some other religion | 13 (0.2%) |
| No religion/Atheist/Agnostic | 972 (15%) |
| (Missing) | 29 (0.5%) |
| ^1^n (%) | |

Table S17b. Childhood predictors regression for Spain

| Variable | Category | Risk-Ratio | RR 95% CI | Global p-value |
| --- | --- | --- | --- | --- |
| Relationship with mother | (Ref: Very bad/somewhat bad) |  |  | 0.190 |
|  | Very good/somewhat good | 1.18 | (0.91,1.52) |  |
| Relationship with father | (Ref: Very bad/somewhat bad) |  |  | 0.170 |
|  | Very good/somewhat good | 1.17 | (0.93,1.46) |  |
| Parent marital status | (Ref: Parents married) |  |  | 0.061 |
|  | Divorced | 1.37 | (1.07,1.76) |  |
|  | Parents were never married | 0.98 | (0.72,1.33) |  |
|  | One or both parents had died | 1.09 | (0.74,1.60) |  |
| Subjective financial status of family growing up | (Ref: Got by) |  |  | 0.286 |
|  | Lived comfortably | 1.05 | (0.89,1.24) |  |
|  | Found it difficult | 1.18 | (0.98,1.42) |  |
|  | Found it very difficult | 1.25 | (0.86,1.81) |  |
| Abuse | (Ref: No) |  |  | <.001 |
|  | Yes | 1.47 | (1.24,1.75) |  |
| Outsider growing up | (Ref: No) |  |  | <.001 |
|  | Yes | 1.60 | (1.33,1.93) |  |
| Self-rated health growing up | (Ref: Good) |  |  | <.001 |
|  | Excellent | 0.74 | (0.61,0.89) |  |
|  | Very good | 0.76 | (0.63,0.92) |  |
|  | Fair | 1.38 | (1.00,1.90) |  |
|  | Poor | 1.50 | (1.07,2.10) |  |
| Immigration status | (Ref: Born in this country) |  |  | 0.017 |
|  | Born in another country | 0.78 | (0.63,0.96) |  |
| Age 12 religious service attendance | (Ref: Never) |  |  | 0.022 |
|  | At least 1/week | 1.18 | (0.97,1.43) |  |
|  | 1-3/month | 0.88 | (0.70,1.11) |  |
|  | < 1/month | 1.04 | (0.84,1.29) |  |
| Year of birth | (Ref: 1998-2005; current age: 18-24) |  |  | <.001 |
|  | 1993-1998; age 25-29 | 1.27 | (0.89,1.81) |  |
|  | 1983-1993; age 30-39 | 1.23 | (0.90,1.67) |  |
|  | 1973-1983; age 40-49 | 1.68 | (1.25,2.25) |  |
|  | 1963-1973; age 50-59 | 2.16 | (1.60,2.90) |  |
|  | 1953-1963; age 60-69 | 2.25 | (1.64,3.10) |  |
|  | 1943-1953; age 70-79 | 3.08 | (2.16,4.38) |  |
|  | 1943 or earlier; age 80+ | 4.11 | (2.20,7.69) |  |
| Gender | (Ref: Male) |  |  | 0.200 |
|  | Female | 1.01 | (0.88,1.16) |  |
|  | Other | 1.74 | (0.82,3.69) |  |
| Religious affiliation | (Ref: No religion/Atheist/Agnostic) |  |  | 0.604 |
|  | Christianity | 1.12 | (0.90,1.39) |  |
|  | Collapsed affiliations with prevalence<3% | 1.07 | (0.66,1.73) |  |
| Race/ethnicity | (Ref: Plurality group) |  |  |  |

Table S17c. Sensitivity to unmeasured confounding of childhood predictors in Spain

| Variable | Category | E-value for Estimate | E-value for 95% CI |
| --- | --- | --- | --- |
| Relationship with mother | (Ref: Very bad/somewhat bad) |  |  |
|  | Very good/somewhat good | 1.64 | 1.00 |
| Relationship with father | (Ref: Very bad/somewhat bad) |  |  |
|  | Very good/somewhat good | 1.60 | 1.00 |
| Parent marital status | (Ref: Parents married) |  |  |
|  | Divorced | 2.09 | 1.35 |
|  | Parents were never married | 1.16 | 1.00 |
|  | One or both parents had died | 1.39 | 1.00 |
| Subjective financial status of family growing up | (Ref: Got by) |  |  |
|  | Lived comfortably | 1.28 | 1.00 |
|  | Found it difficult | 1.64 | 1.00 |
|  | Found it very difficult | 1.81 | 1.00 |
| Abuse | (Ref: No) |  |  |
|  | Yes | 2.31 | 1.77 |
| Outsider growing up | (Ref: No) |  |  |
|  | Yes | 2.59 | 2.00 |
| Self-rated health growing up | (Ref: Good) |  |  |
|  | Excellent | 2.06 | 1.50 |
|  | Very good | 1.96 | 1.41 |
|  | Fair | 2.11 | 1.05 |
|  | Poor | 2.36 | 1.34 |
| Immigration status | (Ref: Born in this country) |  |  |
|  | Born in another country | 1.90 | 1.26 |
| Age 12 religious service attendance | (Ref: Never) |  |  |
|  | At least 1/week | 1.64 | 1.00 |
|  | 1-3/month | 1.52 | 1.00 |
|  | < 1/month | 1.24 | 1.00 |
| Year of birth | (Ref: 1998-2005; current age: 18-24) |  |  |
|  | 1993-1998; age 25-29 | 1.85 | 1.00 |
|  | 1983-1993; age 30-39 | 1.75 | 1.00 |
|  | 1973-1983; age 40-49 | 2.74 | 1.82 |
|  | 1963-1973; age 50-59 | 3.73 | 2.59 |
|  | 1953-1963; age 60-69 | 3.93 | 2.66 |
|  | 1943-1953; age 70-79 | 5.60 | 3.74 |
|  | 1943 or earlier; age 80+ | 7.69 | 3.82 |
| Gender | (Ref: Male) |  |  |
|  | Female | 1.11 | 1.00 |
|  | Other | 2.88 | 1.00 |
| Religious affiliation | (Ref: No religion/Atheist/Agnostic) |  |  |
|  | Christianity | 1.47 | 1.00 |
|  | Collapsed affiliations with prevalence<3% | 1.35 | 1.00 |
| Race/ethnicity | (Ref: Plurality group) |  |  |

Table S18a. Nationally representative descriptive statistics for Sweden

| **Characteristic** | **N = 15,068**^1^ |
| --- | --- |
| **Relationship with mother** |  |
| Very good | 8,743 (58%) |
| Somewhat good | 4,513 (30%) |
| Somewhat bad | 1,194 (7.9%) |
| Very bad | 371 (2.5%) |
| Does not apply | 216 (1.4%) |
| (Missing) | 30 (0.2%) |
| **Relationship with father** |  |
| Very good | 7,134 (47%) |
| Somewhat good | 4,885 (32%) |
| Somewhat bad | 1,588 (11%) |
| Very bad | 725 (4.8%) |
| Does not apply | 720 (4.8%) |
| (Missing) | 16 (0.1%) |
| **Parent marital status** |  |
| Parents married | 10,887 (72%) |
| Divorced | 1,927 (13%) |
| Parents were never married | 1,747 (12%) |
| One or both parents had died | 362 (2.4%) |
| (Missing) | 145 (1.0%) |
| **Subjective financial status of family growing up** |  |
| Lived comfortably | 5,951 (39%) |
| Got by | 7,717 (51%) |
| Found it difficult | 1,238 (8.2%) |
| Found it very difficult | 140 (0.9%) |
| (Missing) | 22 (0.1%) |
| **Abuse** |  |
| Yes | 2,288 (15%) |
| No | 12,735 (85%) |
| (Missing) | 45 (0.3%) |
| **Outsider growing up** |  |
| Yes | 1,867 (12%) |
| No | 13,034 (86%) |
| (Missing) | 168 (1.1%) |
| **Self-rated health growing up** |  |
| Excellent | 5,733 (38%) |
| Very good | 5,124 (34%) |
| Good | 2,669 (18%) |
| Fair | 1,108 (7.4%) |
| Poor | 397 (2.6%) |
| (Missing) | 38 (0.2%) |
| **Immigration status** |  |
| Born in this country | 13,922 (92%) |
| Born in another country | 1,052 (7.0%) |
| (Missing) | 94 (0.6%) |
| **Age 12 religious service attendance** |  |
| At least 1/week | 955 (6.3%) |
| 1-3/month | 1,362 (9.0%) |
| <1/month | 6,224 (41%) |
| Never | 6,472 (43%) |
| (Missing) | 54 (0.4%) |
| **Year of birth** |  |
| 1998-2005; age 18-24 | 1,515 (10%) |
| 1993-1998; age 25-29 | 1,399 (9.3%) |
| 1983-1993; age 30-39 | 2,398 (16%) |
| 1973-1983; age 40-49 | 2,221 (15%) |
| 1963-1973; age 50-59 | 2,493 (17%) |
| 1953-1963; age 60-69 | 2,168 (14%) |
| 1943-1953; age 70-79 | 2,253 (15%) |
| 1943 or earlier; age 80+ | 621 (4.1%) |
| (Missing) | 0 (0%) |
| **Gender** |  |
| Male | 7,536 (50%) |
| Female | 7,493 (50%) |
| Other | 27 (0.2%) |
| (Missing) | 12 (<0.1%) |
| **Religious affiliation** |  |
| Christianity | 10,617 (70%) |
| Islam | 462 (3.1%) |
| Hinduism | 16 (0.1%) |
| Buddhism | 41 (0.3%) |
| Judaism | 51 (0.3%) |
| Sikhism | 9 (<0.1%) |
| Baha'i | 3 (<0.1%) |
| Jainism | 0 (0%) |
| Shinto | 1 (<0.1%) |
| Taoism | 0 (0%) |
| Confucianism | 4 (<0.1%) |
| Primal, Animist, or Folk religion | 31 (0.2%) |
| Spiritism | 0 (0%) |
| Umbanda, Candomble, and other African-derived religions | 0 (0%) |
| Chinese folk/traditional religion | 0 (0%) |
| Some other religion | 69 (0.5%) |
| No religion/Atheist/Agnostic | 3,738 (25%) |
| (Missing) | 26 (0.2%) |
| ^1^n (%) | |

Table S18b. Childhood predictors regression for Sweden

| Variable | Category | Risk-Ratio | RR 95% CI | Global p-value |
| --- | --- | --- | --- | --- |
| Relationship with mother | (Ref: Very bad/somewhat bad) |  |  | 0.189 |
|  | Very good/somewhat good | 1.07 | (0.97,1.18) |  |
| Relationship with father | (Ref: Very bad/somewhat bad) |  |  | 0.762 |
|  | Very good/somewhat good | 1.01 | (0.93,1.11) |  |
| Parent marital status | (Ref: Parents married) |  |  | 0.013 |
|  | Divorced | 0.95 | (0.86,1.05) |  |
|  | Parents were never married | 1.16 | (1.04,1.29) |  |
|  | One or both parents had died | 1.05 | (0.85,1.30) |  |
| Subjective financial status of family growing up | (Ref: Got by) |  |  | <.001 |
|  | Lived comfortably | 0.88 | (0.82,0.95) |  |
|  | Found it difficult | 1.15 | (1.04,1.27) |  |
|  | Found it very difficult | 1.15 | (0.91,1.47) |  |
| Abuse | (Ref: No) |  |  | <.001 |
|  | Yes | 1.32 | (1.22,1.44) |  |
| Outsider growing up | (Ref: No) |  |  | 0.014 |
|  | Yes | 1.13 | (1.02,1.25) |  |
| Self-rated health growing up | (Ref: Good) |  |  | <.001 |
|  | Excellent | 0.56 | (0.51,0.62) |  |
|  | Very good | 0.75 | (0.68,0.82) |  |
|  | Fair | 1.28 | (1.16,1.42) |  |
|  | Poor | 1.61 | (1.41,1.84) |  |
| Immigration status | (Ref: Born in this country) |  |  | 0.673 |
|  | Born in another country | 0.97 | (0.85,1.11) |  |
| Age 12 religious service attendance | (Ref: Never) |  |  | 0.002 |
|  | At least 1/week | 1.17 | (1.02,1.34) |  |
|  | 1-3/month | 1.15 | (1.03,1.29) |  |
|  | < 1/month | 0.96 | (0.89,1.03) |  |
| Year of birth | (Ref: 1998-2005; current age: 18-24) |  |  | <.001 |
|  | 1993-1998; age 25-29 | 1.02 | (0.86,1.21) |  |
|  | 1983-1993; age 30-39 | 1.08 | (0.93,1.25) |  |
|  | 1973-1983; age 40-49 | 1.35 | (1.16,1.57) |  |
|  | 1963-1973; age 50-59 | 1.71 | (1.48,1.98) |  |
|  | 1953-1963; age 60-69 | 2.27 | (1.97,2.63) |  |
|  | 1943-1953; age 70-79 | 2.16 | (1.86,2.51) |  |
|  | 1943 or earlier; age 80+ | 2.29 | (1.89,2.76) |  |
| Gender | (Ref: Male) |  |  | <.001 |
|  | Female | 1.21 | (1.13,1.29) |  |
|  | Other | 2.79 | (2.00,3.91) |  |
| Religious affiliation | (Ref: No religion/Atheist/Agnostic) |  |  | 0.099 |
|  | Islam | 0.98 | (0.75,1.28) |  |
|  | Christianity | 0.91 | (0.84,0.98) |  |
|  | Collapsed affiliations with prevalence<3% | 0.87 | (0.65,1.16) |  |
| Race/ethnicity | (Ref: Plurality group) |  |  |  |

Table S18c. Sensitivity to unmeasured confounding of childhood predictors in Sweden

| Variable | Category | E-value for Estimate | E-value for 95% CI |
| --- | --- | --- | --- |
| Relationship with mother | (Ref: Very bad/somewhat bad) |  |  |
|  | Very good/somewhat good | 1.34 | 1.00 |
| Relationship with father | (Ref: Very bad/somewhat bad) |  |  |
|  | Very good/somewhat good | 1.13 | 1.00 |
| Parent marital status | (Ref: Parents married) |  |  |
|  | Divorced | 1.30 | 1.00 |
|  | Parents were never married | 1.59 | 1.24 |
|  | One or both parents had died | 1.28 | 1.00 |
| Subjective financial status of family growing up | (Ref: Got by) |  |  |
|  | Lived comfortably | 1.52 | 1.28 |
|  | Found it difficult | 1.57 | 1.24 |
|  | Found it very difficult | 1.57 | 1.00 |
| Abuse | (Ref: No) |  |  |
|  | Yes | 1.98 | 1.74 |
| Outsider growing up | (Ref: No) |  |  |
|  | Yes | 1.51 | 1.18 |
| Self-rated health growing up | (Ref: Good) |  |  |
|  | Excellent | 2.97 | 2.63 |
|  | Very good | 2.01 | 1.75 |
|  | Fair | 1.89 | 1.59 |
|  | Poor | 2.60 | 2.16 |
| Immigration status | (Ref: Born in this country) |  |  |
|  | Born in another country | 1.20 | 1.00 |
| Age 12 religious service attendance | (Ref: Never) |  |  |
|  | At least 1/week | 1.61 | 1.16 |
|  | 1-3/month | 1.57 | 1.19 |
|  | < 1/month | 1.24 | 1.00 |
| Year of birth | (Ref: 1998-2005; current age: 18-24) |  |  |
|  | 1993-1998; age 25-29 | 1.15 | 1.00 |
|  | 1983-1993; age 30-39 | 1.36 | 1.00 |
|  | 1973-1983; age 40-49 | 2.04 | 1.60 |
|  | 1963-1973; age 50-59 | 2.82 | 2.33 |
|  | 1953-1963; age 60-69 | 3.97 | 3.35 |
|  | 1943-1953; age 70-79 | 3.75 | 3.12 |
|  | 1943 or earlier; age 80+ | 4.00 | 3.20 |
| Gender | (Ref: Male) |  |  |
|  | Female | 1.71 | 1.51 |
|  | Other | 5.03 | 3.41 |
| Religious affiliation | (Ref: No religion/Atheist/Agnostic) |  |  |
|  | Islam | 1.18 | 1.00 |
|  | Christianity | 1.44 | 1.16 |
|  | Collapsed affiliations with prevalence<3% | 1.58 | 1.00 |
| Race/ethnicity | (Ref: Plurality group) |  |  |

Table S19a. Nationally representative descriptive statistics for Tanzania

| **Characteristic** | **N = 9,075**^1^ |
| --- | --- |
| **Relationship with mother** |  |
| Very good | 7,739 (85%) |
| Somewhat good | 796 (8.8%) |
| Somewhat bad | 84 (0.9%) |
| Very bad | 84 (0.9%) |
| Does not apply | 303 (3.3%) |
| (Missing) | 70 (0.8%) |
| **Relationship with father** |  |
| Very good | 6,831 (75%) |
| Somewhat good | 1,101 (12%) |
| Somewhat bad | 203 (2.2%) |
| Very bad | 247 (2.7%) |
| Does not apply | 550 (6.1%) |
| (Missing) | 142 (1.6%) |
| **Parent marital status** |  |
| Parents married | 6,929 (76%) |
| Divorced | 678 (7.5%) |
| Parents were never married | 751 (8.3%) |
| One or both parents had died | 313 (3.4%) |
| (Missing) | 404 (4.4%) |
| **Subjective financial status of family growing up** |  |
| Lived comfortably | 2,611 (29%) |
| Got by | 2,909 (32%) |
| Found it difficult | 2,679 (30%) |
| Found it very difficult | 814 (9.0%) |
| (Missing) | 61 (0.7%) |
| **Abuse** |  |
| Yes | 716 (7.9%) |
| No | 8,328 (92%) |
| (Missing) | 32 (0.3%) |
| **Outsider growing up** |  |
| Yes | 734 (8.1%) |
| No | 8,320 (92%) |
| (Missing) | 22 (0.2%) |
| **Self-rated health growing up** |  |
| Excellent | 2,406 (27%) |
| Very good | 2,036 (22%) |
| Good | 2,946 (32%) |
| Fair | 1,177 (13%) |
| Poor | 456 (5.0%) |
| (Missing) | 54 (0.6%) |
| **Immigration status** |  |
| Born in this country | 9,048 (100%) |
| Born in another country | 25 (0.3%) |
| (Missing) | 1 (<0.1%) |
| **Age 12 religious service attendance** |  |
| At least 1/week | 5,580 (61%) |
| 1-3/month | 2,383 (26%) |
| <1/month | 333 (3.7%) |
| Never | 595 (6.6%) |
| (Missing) | 184 (2.0%) |
| **Year of birth** |  |
| 1998-2005; age 18-24 | 2,284 (25%) |
| 1993-1998; age 25-29 | 1,349 (15%) |
| 1983-1993; age 30-39 | 2,060 (23%) |
| 1973-1983; age 40-49 | 1,503 (17%) |
| 1963-1973; age 50-59 | 912 (10%) |
| 1953-1963; age 60-69 | 575 (6.3%) |
| 1943-1953; age 70-79 | 297 (3.3%) |
| 1943 or earlier; age 80+ | 93 (1.0%) |
| (Missing) | 2 (<0.1%) |
| **Gender** |  |
| Male | 4,299 (47%) |
| Female | 4,776 (53%) |
| Other | 0 (0%) |
| (Missing) | 0 (0%) |
| **Religious affiliation** |  |
| Christianity | 5,651 (62%) |
| Islam | 3,060 (34%) |
| Hinduism | 0 (0%) |
| Buddhism | 0 (0%) |
| Judaism | 0 (0%) |
| Sikhism | 0 (0%) |
| Baha'i | 1 (<0.1%) |
| Jainism | 0 (0%) |
| Shinto | 0 (0%) |
| Taoism | 0 (0%) |
| Confucianism | 0 (0%) |
| Primal, Animist, or Folk religion | 11 (0.1%) |
| Spiritism | 0 (0%) |
| Umbanda, Candomble, and other African-derived religions | 0 (0%) |
| Chinese folk/traditional religion | 0 (0%) |
| Some other religion | 0 (0%) |
| No religion/Atheist/Agnostic | 345 (3.8%) |
| (Missing) | 7 (<0.1%) |
| **Race/Ethnicity** |  |
| African | 9,060 (100%) |
| Arab | 11 (0.1%) |
| Indian | 3 (<0.1%) |
| (Missing) | 2 (<0.1%) |
| ^1^n (%) | |

Table S19b. Childhood predictors regression for Tanzania

| Variable | Category | Risk-Ratio | RR 95% CI | Global p-value |
| --- | --- | --- | --- | --- |
| Relationship with mother | (Ref: Very bad/somewhat bad) |  |  | 0.242 |
|  | Very good/somewhat good | 0.84 | (0.62,1.14) |  |
| Relationship with father | (Ref: Very bad/somewhat bad) |  |  | 0.817 |
|  | Very good/somewhat good | 1.02 | (0.80,1.30) |  |
| Parent marital status | (Ref: Parents married) |  |  | 0.009 |
|  | Divorced | 1.09 | (0.86,1.39) |  |
|  | Parents were never married | 1.03 | (0.81,1.31) |  |
|  | One or both parents had died | 1.50 | (1.18,1.90) |  |
| Subjective financial status of family growing up | (Ref: Got by) |  |  | 0.517 |
|  | Lived comfortably | 1.07 | (0.92,1.24) |  |
|  | Found it difficult | 1.10 | (0.96,1.26) |  |
|  | Found it very difficult | 1.09 | (0.87,1.37) |  |
| Abuse | (Ref: No) |  |  | <.001 |
|  | Yes | 1.94 | (1.66,2.27) |  |
| Outsider growing up | (Ref: No) |  |  | 0.037 |
|  | Yes | 1.24 | (1.01,1.51) |  |
| Self-rated health growing up | (Ref: Good) |  |  | <.001 |
|  | Excellent | 0.86 | (0.73,1.01) |  |
|  | Very good | 0.96 | (0.83,1.10) |  |
|  | Fair | 0.97 | (0.81,1.16) |  |
|  | Poor | 1.75 | (1.37,2.25) |  |
| Immigration status | (Ref: Born in this country) |  |  | 0.929 |
|  | Born in another country | 0.92 | (0.16,5.50) |  |
| Age 12 religious service attendance | (Ref: Never) |  |  | 0.072 |
|  | At least 1/week | 0.84 | (0.64,1.10) |  |
|  | 1-3/month | 0.99 | (0.75,1.31) |  |
|  | < 1/month | 1.07 | (0.75,1.52) |  |
| Year of birth | (Ref: 1998-2005; current age: 18-24) |  |  | <.001 |
|  | 1993-1998; age 25-29 | 1.67 | (1.27,2.22) |  |
|  | 1983-1993; age 30-39 | 1.90 | (1.50,2.40) |  |
|  | 1973-1983; age 40-49 | 2.63 | (2.05,3.39) |  |
|  | 1963-1973; age 50-59 | 4.34 | (3.39,5.56) |  |
|  | 1953-1963; age 60-69 | 6.51 | (5.00,8.46) |  |
|  | 1943-1953; age 70-79 | 9.64 | (7.21,12.89) |  |
|  | 1943 or earlier; age 80+ | 11.92 | (8.40,16.93) |  |
| Gender | (Ref: Male) |  |  | <.001 |
|  | Female | 1.62 | (1.43,1.84) |  |
| Religious affiliation | (Ref: No religion/Atheist/Agnostic) |  |  | 0.397 |
|  | Islam | 1.18 | (0.84,1.65) |  |
|  | Christianity | 1.17 | (0.84,1.64) |  |
|  | Collapsed affiliations with prevalence<3% | 1.96 | (0.82,4.66) |  |
| Race/ethnicity | (Ref: Plurality group) |  |  | 0.236 |
|  | Non-plurality groups | 1.86 | (0.67,5.16) |  |

Table S19c. Sensitivity to unmeasured confounding of childhood predictors in Tanzania

| Variable | Category | E-value for Estimate | E-value for 95% CI |
| --- | --- | --- | --- |
| Relationship with mother | (Ref: Very bad/somewhat bad) |  |  |
|  | Very good/somewhat good | 1.68 | 1.00 |
| Relationship with father | (Ref: Very bad/somewhat bad) |  |  |
|  | Very good/somewhat good | 1.17 | 1.00 |
| Parent marital status | (Ref: Parents married) |  |  |
|  | Divorced | 1.41 | 1.00 |
|  | Parents were never married | 1.20 | 1.00 |
|  | One or both parents had died | 2.36 | 1.63 |
| Subjective financial status of family growing up | (Ref: Got by) |  |  |
|  | Lived comfortably | 1.33 | 1.00 |
|  | Found it difficult | 1.44 | 1.00 |
|  | Found it very difficult | 1.41 | 1.00 |
| Abuse | (Ref: No) |  |  |
|  | Yes | 3.29 | 2.70 |
| Outsider growing up | (Ref: No) |  |  |
|  | Yes | 1.78 | 1.14 |
| Self-rated health growing up | (Ref: Good) |  |  |
|  | Excellent | 1.60 | 1.00 |
|  | Very good | 1.25 | 1.00 |
|  | Fair | 1.22 | 1.00 |
|  | Poor | 2.90 | 2.07 |
| Immigration status | (Ref: Born in this country) |  |  |
|  | Born in another country | 1.38 | 1.00 |
| Age 12 religious service attendance | (Ref: Never) |  |  |
|  | At least 1/week | 1.67 | 1.00 |
|  | 1-3/month | 1.12 | 1.00 |
|  | < 1/month | 1.34 | 1.00 |
| Year of birth | (Ref: 1998-2005; current age: 18-24) |  |  |
|  | 1993-1998; age 25-29 | 2.74 | 1.84 |
|  | 1983-1993; age 30-39 | 3.20 | 2.36 |
|  | 1973-1983; age 40-49 | 4.71 | 3.51 |
|  | 1963-1973; age 50-59 | 8.15 | 6.24 |
|  | 1953-1963; age 60-69 | 12.50 | 9.48 |
|  | 1943-1953; age 70-79 | 18.76 | 13.90 |
|  | 1943 or earlier; age 80+ | 23.34 | 16.29 |
| Gender | (Ref: Male) |  |  |
|  | Female | 2.62 | 2.21 |
| Religious affiliation | (Ref: No religion/Atheist/Agnostic) |  |  |
|  | Islam | 1.63 | 1.00 |
|  | Christianity | 1.62 | 1.00 |
|  | Collapsed affiliations with prevalence<3% | 3.33 | 1.00 |
| Race/ethnicity | (Ref: Plurality group) |  |  |
|  | Non-plurality groups | 3.12 | 1.00 |

Table S20a. Nationally representative descriptive statistics for Turkey

| **Characteristic** | **N = 1,473**^1^ |
| --- | --- |
| **Relationship with mother** |  |
| Very good | 970 (66%) |
| Somewhat good | 401 (27%) |
| Somewhat bad | 48 (3.2%) |
| Very bad | 26 (1.8%) |
| Does not apply | 21 (1.4%) |
| (Missing) | 7 (0.5%) |
| **Relationship with father** |  |
| Very good | 795 (54%) |
| Somewhat good | 425 (29%) |
| Somewhat bad | 73 (5.0%) |
| Very bad | 95 (6.5%) |
| Does not apply | 60 (4.1%) |
| (Missing) | 25 (1.7%) |
| **Parent marital status** |  |
| Parents married | 1,325 (90%) |
| Divorced | 57 (3.9%) |
| Parents were never married | 7 (0.5%) |
| One or both parents had died | 61 (4.1%) |
| (Missing) | 23 (1.5%) |
| **Subjective financial status of family growing up** |  |
| Lived comfortably | 498 (34%) |
| Got by | 647 (44%) |
| Found it difficult | 218 (15%) |
| Found it very difficult | 108 (7.3%) |
| (Missing) | 2 (0.1%) |
| **Abuse** |  |
| Yes | 158 (11%) |
| No | 1,290 (88%) |
| (Missing) | 25 (1.7%) |
| **Outsider growing up** |  |
| Yes | 157 (11%) |
| No | 1,306 (89%) |
| (Missing) | 9 (0.6%) |
| **Self-rated health growing up** |  |
| Excellent | 377 (26%) |
| Very good | 410 (28%) |
| Good | 419 (28%) |
| Fair | 220 (15%) |
| Poor | 47 (3.2%) |
| (Missing) | 0 (<0.1%) |
| **Immigration status** |  |
| Born in this country | 1,415 (96%) |
| Born in another country | 58 (4.0%) |
| (Missing) | 0 (0%) |
| **Age 12 religious service attendance** |  |
| At least 1/week | 609 (41%) |
| 1-3/month | 238 (16%) |
| <1/month | 225 (15%) |
| Never | 383 (26%) |
| (Missing) | 18 (1.2%) |
| **Year of birth** |  |
| 1998-2005; age 18-24 | 222 (15%) |
| 1993-1998; age 25-29 | 152 (10%) |
| 1983-1993; age 30-39 | 315 (21%) |
| 1973-1983; age 40-49 | 312 (21%) |
| 1963-1973; age 50-59 | 225 (15%) |
| 1953-1963; age 60-69 | 164 (11%) |
| 1943-1953; age 70-79 | 65 (4.4%) |
| 1943 or earlier; age 80+ | 18 (1.2%) |
| (Missing) | 0 (0%) |
| **Gender** |  |
| Male | 754 (51%) |
| Female | 719 (49%) |
| Other | 0 (0%) |
| (Missing) | 0 (0%) |
| **Religious affiliation** |  |
| Christianity | 1 (<0.1%) |
| Islam | 1,439 (98%) |
| Hinduism | 0 (0%) |
| Buddhism | 0 (0%) |
| Judaism | 1 (<0.1%) |
| Sikhism | 0 (0%) |
| Baha'i | 0 (0%) |
| Jainism | 0 (0%) |
| Shinto | 0 (0%) |
| Taoism | 0 (0%) |
| Confucianism | 0 (0%) |
| Primal, Animist, or Folk religion | 0 (0%) |
| Spiritism | 0 (0%) |
| Umbanda, Candomble, and other African-derived religions | 0 (0%) |
| Chinese folk/traditional religion | 0 (0%) |
| Some other religion | 0 (0%) |
| No religion/Atheist/Agnostic | 13 (0.9%) |
| (Missing) | 19 (1.3%) |
| **Race/Ethnicity** |  |
| Albanian | 8 (0.5%) |
| Arab | 51 (3.5%) |
| Armenian | 1 (<0.1%) |
| Azeri | 9 (0.6%) |
| Bosnian | 5 (0.3%) |
| Circassian | 19 (1.3%) |
| Georgian | 4 (0.3%) |
| Greek | 1 (<0.1%) |
| Kurdish/Zaza | 252 (17%) |
| Laz | 25 (1.7%) |
| Other | 58 (3.9%) |
| Turkish | 1,030 (70%) |
| Uyghur | 1 (<0.1%) |
| (Missing) | 9 (0.6%) |
| ^1^n (%) | |

Table S20b. Childhood predictors regression for Turkey

| Variable | Category | Risk-Ratio | RR 95% CI | Global p-value |
| --- | --- | --- | --- | --- |
| Relationship with mother | (Ref: Very bad/somewhat bad) |  |  | 0.151 |
|  | Very good/somewhat good | 1.57 | (0.83,3.00) |  |
| Relationship with father | (Ref: Very bad/somewhat bad) |  |  | 0.367 |
|  | Very good/somewhat good | 1.24 | (0.77,2.01) |  |
| Parent marital status | (Ref: Parents married) |  |  | 0.762 |
|  | Divorced | 1.04 | (0.36,2.96) |  |
|  | Parents were never married | 1.49 | (0.25,8.77) |  |
|  | One or both parents had died | 0.69 | (0.31,1.53) |  |
| Subjective financial status of family growing up | (Ref: Got by) |  |  | 0.203 |
|  | Lived comfortably | 1.02 | (0.70,1.47) |  |
|  | Found it difficult | 0.59 | (0.35,1.00) |  |
|  | Found it very difficult | 0.75 | (0.37,1.51) |  |
| Abuse | (Ref: No) |  |  | 0.861 |
|  | Yes | 0.96 | (0.59,1.58) |  |
| Outsider growing up | (Ref: No) |  |  | 0.585 |
|  | Yes | 1.13 | (0.73,1.75) |  |
| Self-rated health growing up | (Ref: Good) |  |  | <.001 |
|  | Excellent | 0.66 | (0.39,1.11) |  |
|  | Very good | 0.73 | (0.43,1.24) |  |
|  | Fair | 1.73 | (1.10,2.72) |  |
|  | Poor | 5.33 | (3.43,8.28) |  |
| Immigration status | (Ref: Born in this country) |  |  | 0.909 |
|  | Born in another country | 0.95 | (0.35,2.60) |  |
| Age 12 religious service attendance | (Ref: Never) |  |  | 0.519 |
|  | At least 1/week | 0.82 | (0.53,1.27) |  |
|  | 1-3/month | 0.71 | (0.41,1.24) |  |
|  | < 1/month | 0.70 | (0.40,1.22) |  |
| Year of birth | (Ref: 1998-2005; current age: 18-24) |  |  | <.001 |
|  | 1993-1998; age 25-29 | 0.56 | (0.27,1.20) |  |
|  | 1983-1993; age 30-39 | 1.05 | (0.62,1.78) |  |
|  | 1973-1983; age 40-49 | 1.35 | (0.84,2.19) |  |
|  | 1963-1973; age 50-59 | 1.11 | (0.60,2.06) |  |
|  | 1953-1963; age 60-69 | 1.89 | (1.06,3.36) |  |
|  | 1943-1953; age 70-79 | 2.94 | (1.30,6.67) |  |
|  | 1943 or earlier; age 80+ | 0.00 | (0.00,20737.28) |  |
| Gender | (Ref: Male) |  |  | 0.140 |
|  | Female | 1.28 | (0.92,1.78) |  |
| Religious affiliation | (Ref: Islam) |  |  | 0.165 |
|  | Collapsed affiliations with prevalence<3% | 0.26 | (0.03,2.22) |  |
| Race/ethnicity | (Ref: Plurality group) |  |  | 0.614 |
|  | Non-plurality groups | 0.94 | (0.62,1.41) |  |

Table S20c. Sensitivity to unmeasured confounding of childhood predictors in Turkey

| Variable | Category | E-value for Estimate | E-value for 95% CI |
| --- | --- | --- | --- |
| Relationship with mother | (Ref: Very bad/somewhat bad) |  |  |
|  | Very good/somewhat good | 2.52 | 1.00 |
| Relationship with father | (Ref: Very bad/somewhat bad) |  |  |
|  | Very good/somewhat good | 1.79 | 1.00 |
| Parent marital status | (Ref: Parents married) |  |  |
|  | Divorced | 1.23 | 1.00 |
|  | Parents were never married | 2.34 | 1.00 |
|  | One or both parents had died | 2.24 | 1.00 |
| Subjective financial status of family growing up | (Ref: Got by) |  |  |
|  | Lived comfortably | 1.16 | 1.00 |
|  | Found it difficult | 2.78 | 1.00 |
|  | Found it very difficult | 2.00 | 1.00 |
| Abuse | (Ref: No) |  |  |
|  | Yes | 1.23 | 1.00 |
| Outsider growing up | (Ref: No) |  |  |
|  | Yes | 1.51 | 1.00 |
| Self-rated health growing up | (Ref: Good) |  |  |
|  | Excellent | 2.42 | 1.00 |
|  | Very good | 2.08 | 1.00 |
|  | Fair | 2.86 | 1.44 |
|  | Poor | 10.14 | 6.32 |
| Immigration status | (Ref: Born in this country) |  |  |
|  | Born in another country | 1.30 | 1.00 |
| Age 12 religious service attendance | (Ref: Never) |  |  |
|  | At least 1/week | 1.73 | 1.00 |
|  | 1-3/month | 2.15 | 1.00 |
|  | < 1/month | 2.21 | 1.00 |
| Year of birth | (Ref: 1998-2005; current age: 18-24) |  |  |
|  | 1993-1998; age 25-29 | 2.95 | 1.00 |
|  | 1983-1993; age 30-39 | 1.29 | 1.00 |
|  | 1973-1983; age 40-49 | 2.05 | 1.00 |
|  | 1963-1973; age 50-59 | 1.45 | 1.00 |
|  | 1953-1963; age 60-69 | 3.18 | 1.32 |
|  | 1943-1953; age 70-79 | 5.33 | 1.91 |
|  | 1943 or earlier; age 80+ | 135437.94 | 1.00 |
| Gender | (Ref: Male) |  |  |
|  | Female | 1.87 | 1.00 |
| Religious affiliation | (Ref: Islam) |  |  |
|  | Collapsed affiliations with prevalence<3% | 7.16 | 1.00 |
| Race/ethnicity | (Ref: Plurality group) |  |  |
|  | Non-plurality groups | 1.33 | 1.00 |

Table S21a. Nationally representative descriptive statistics for United Kingdom

| **Characteristic** | **N = 5,368**^1^ |
| --- | --- |
| **Relationship with mother** |  |
| Very good | 3,435 (64%) |
| Somewhat good | 1,338 (25%) |
| Somewhat bad | 325 (6.1%) |
| Very bad | 150 (2.8%) |
| Does not apply | 92 (1.7%) |
| (Missing) | 27 (0.5%) |
| **Relationship with father** |  |
| Very good | 2,907 (54%) |
| Somewhat good | 1,383 (26%) |
| Somewhat bad | 407 (7.6%) |
| Very bad | 321 (6.0%) |
| Does not apply | 321 (6.0%) |
| (Missing) | 29 (0.5%) |
| **Parent marital status** |  |
| Parents married | 4,343 (81%) |
| Divorced | 481 (9.0%) |
| Parents were never married | 315 (5.9%) |
| One or both parents had died | 154 (2.9%) |
| (Missing) | 75 (1.4%) |
| **Subjective financial status of family growing up** |  |
| Lived comfortably | 2,552 (48%) |
| Got by | 1,933 (36%) |
| Found it difficult | 632 (12%) |
| Found it very difficult | 230 (4.3%) |
| (Missing) | 22 (0.4%) |
| **Abuse** |  |
| Yes | 864 (16%) |
| No | 4,455 (83%) |
| (Missing) | 49 (0.9%) |
| **Outsider growing up** |  |
| Yes | 1,017 (19%) |
| No | 4,308 (80%) |
| (Missing) | 43 (0.8%) |
| **Self-rated health growing up** |  |
| Excellent | 2,154 (40%) |
| Very good | 1,736 (32%) |
| Good | 995 (19%) |
| Fair | 332 (6.2%) |
| Poor | 130 (2.4%) |
| (Missing) | 20 (0.4%) |
| **Immigration status** |  |
| Born in this country | 4,659 (87%) |
| Born in another country | 682 (13%) |
| (Missing) | 27 (0.5%) |
| **Age 12 religious service attendance** |  |
| At least 1/week | 1,732 (32%) |
| 1-3/month | 733 (14%) |
| <1/month | 903 (17%) |
| Never | 1,972 (37%) |
| (Missing) | 28 (0.5%) |
| **Year of birth** |  |
| 1998-2005; age 18-24 | 490 (9.1%) |
| 1993-1998; age 25-29 | 391 (7.3%) |
| 1983-1993; age 30-39 | 946 (18%) |
| 1973-1983; age 40-49 | 827 (15%) |
| 1963-1973; age 50-59 | 949 (18%) |
| 1953-1963; age 60-69 | 889 (17%) |
| 1943-1953; age 70-79 | 711 (13%) |
| 1943 or earlier; age 80+ | 163 (3.0%) |
| (Missing) | 1 (<0.1%) |
| **Gender** |  |
| Male | 2,557 (48%) |
| Female | 2,789 (52%) |
| Other | 14 (0.3%) |
| (Missing) | 9 (0.2%) |
| **Religious affiliation** |  |
| Christianity | 3,461 (64%) |
| Islam | 230 (4.3%) |
| Hinduism | 88 (1.6%) |
| Buddhism | 15 (0.3%) |
| Judaism | 59 (1.1%) |
| Sikhism | 30 (0.6%) |
| Baha'i | 5 (<0.1%) |
| Jainism | 0 (<0.1%) |
| Shinto | 0 (0%) |
| Taoism | 2 (<0.1%) |
| Confucianism | 3 (<0.1%) |
| Primal, Animist, or Folk religion | 22 (0.4%) |
| Spiritism | 0 (0%) |
| Umbanda, Candomble, and other African-derived religions | 0 (0%) |
| Chinese folk/traditional religion | 0 (0%) |
| Some other religion | 24 (0.5%) |
| No religion/Atheist/Agnostic | 1,409 (26%) |
| (Missing) | 21 (0.4%) |
| **Race/Ethnicity** |  |
| Asian | 426 (7.9%) |
| Black | 152 (2.8%) |
| Other | 96 (1.8%) |
| White | 4,647 (87%) |
| (Missing) | 47 (0.9%) |
| ^1^n (%) | |

Table S21b. Childhood predictors regression for United Kingdom

| Variable | Category | Risk-Ratio | RR 95% CI | Global p-value |
| --- | --- | --- | --- | --- |
| Relationship with mother | (Ref: Very bad/somewhat bad) |  |  | 0.885 |
|  | Very good/somewhat good | 1.01 | (0.86,1.19) |  |
| Relationship with father | (Ref: Very bad/somewhat bad) |  |  | 0.745 |
|  | Very good/somewhat good | 1.02 | (0.88,1.18) |  |
| Parent marital status | (Ref: Parents married) |  |  | 0.958 |
|  | Divorced | 1.01 | (0.84,1.22) |  |
|  | Parents were never married | 1.06 | (0.79,1.42) |  |
|  | One or both parents had died | 1.04 | (0.76,1.41) |  |
| Subjective financial status of family growing up | (Ref: Got by) |  |  | 0.142 |
|  | Lived comfortably | 0.88 | (0.78,0.99) |  |
|  | Found it difficult | 0.88 | (0.75,1.03) |  |
|  | Found it very difficult | 0.93 | (0.75,1.17) |  |
| Abuse | (Ref: No) |  |  | <.001 |
|  | Yes | 1.62 | (1.43,1.84) |  |
| Outsider growing up | (Ref: No) |  |  | 0.077 |
|  | Yes | 1.13 | (0.99,1.29) |  |
| Self-rated health growing up | (Ref: Good) |  |  | <.001 |
|  | Excellent | 0.57 | (0.50,0.66) |  |
|  | Very good | 0.66 | (0.57,0.76) |  |
|  | Fair | 1.04 | (0.87,1.24) |  |
|  | Poor | 1.32 | (1.06,1.64) |  |
| Immigration status | (Ref: Born in this country) |  |  | <.001 |
|  | Born in another country | 0.66 | (0.54,0.80) |  |
| Age 12 religious service attendance | (Ref: Never) |  |  | 0.143 |
|  | At least 1/week | 1.02 | (0.89,1.18) |  |
|  | 1-3/month | 0.86 | (0.71,1.06) |  |
|  | < 1/month | 0.89 | (0.75,1.05) |  |
| Year of birth | (Ref: 1998-2005; current age: 18-24) |  |  | <.001 |
|  | 1993-1998; age 25-29 | 1.07 | (0.74,1.54) |  |
|  | 1983-1993; age 30-39 | 1.07 | (0.79,1.44) |  |
|  | 1973-1983; age 40-49 | 1.13 | (0.84,1.52) |  |
|  | 1963-1973; age 50-59 | 1.35 | (1.01,1.81) |  |
|  | 1953-1963; age 60-69 | 1.91 | (1.43,2.54) |  |
|  | 1943-1953; age 70-79 | 1.87 | (1.39,2.51) |  |
|  | 1943 or earlier; age 80+ | 1.95 | (1.36,2.80) |  |
| Gender | (Ref: Male) |  |  | 0.002 |
|  | Female | 1.16 | (1.04,1.29) |  |
|  | Other | 2.28 | (1.24,4.20) |  |
| Religious affiliation | (Ref: No religion/Atheist/Agnostic) |  |  | 0.877 |
|  | Islam | 1.09 | (0.74,1.61) |  |
|  | Christianity | 1.05 | (0.92,1.20) |  |
|  | Collapsed affiliations with prevalence<3% | 1.08 | (0.80,1.44) |  |
| Race/ethnicity | (Ref: Plurality group) |  |  | 0.095 |
|  | Non-plurality groups | 0.82 | (0.63,1.05) |  |

Table S21c. Sensitivity to unmeasured confounding of childhood predictors in United Kingdom

| Variable | Category | E-value for Estimate | E-value for 95% CI |
| --- | --- | --- | --- |
| Relationship with mother | (Ref: Very bad/somewhat bad) |  |  |
|  | Very good/somewhat good | 1.11 | 1.00 |
| Relationship with father | (Ref: Very bad/somewhat bad) |  |  |
|  | Very good/somewhat good | 1.16 | 1.00 |
| Parent marital status | (Ref: Parents married) |  |  |
|  | Divorced | 1.13 | 1.00 |
|  | Parents were never married | 1.31 | 1.00 |
|  | One or both parents had died | 1.23 | 1.00 |
| Subjective financial status of family growing up | (Ref: Got by) |  |  |
|  | Lived comfortably | 1.54 | 1.11 |
|  | Found it difficult | 1.53 | 1.00 |
|  | Found it very difficult | 1.35 | 1.00 |
| Abuse | (Ref: No) |  |  |
|  | Yes | 2.63 | 2.22 |
| Outsider growing up | (Ref: No) |  |  |
|  | Yes | 1.50 | 1.00 |
| Self-rated health growing up | (Ref: Good) |  |  |
|  | Excellent | 2.89 | 2.39 |
|  | Very good | 2.40 | 1.96 |
|  | Fair | 1.25 | 1.00 |
|  | Poor | 1.96 | 1.31 |
| Immigration status | (Ref: Born in this country) |  |  |
|  | Born in another country | 2.41 | 1.80 |
| Age 12 religious service attendance | (Ref: Never) |  |  |
|  | At least 1/week | 1.17 | 1.00 |
|  | 1-3/month | 1.58 | 1.00 |
|  | < 1/month | 1.50 | 1.00 |
| Year of birth | (Ref: 1998-2005; current age: 18-24) |  |  |
|  | 1993-1998; age 25-29 | 1.34 | 1.00 |
|  | 1983-1993; age 30-39 | 1.34 | 1.00 |
|  | 1973-1983; age 40-49 | 1.51 | 1.00 |
|  | 1963-1973; age 50-59 | 2.05 | 1.12 |
|  | 1953-1963; age 60-69 | 3.22 | 2.22 |
|  | 1943-1953; age 70-79 | 3.14 | 2.13 |
|  | 1943 or earlier; age 80+ | 3.31 | 2.06 |
| Gender | (Ref: Male) |  |  |
|  | Female | 1.58 | 1.24 |
|  | Other | 3.99 | 1.78 |
| Religious affiliation | (Ref: No religion/Atheist/Agnostic) |  |  |
|  | Islam | 1.41 | 1.00 |
|  | Christianity | 1.28 | 1.00 |
|  | Collapsed affiliations with prevalence<3% | 1.36 | 1.00 |
| Race/ethnicity | (Ref: Plurality group) |  |  |
|  | Non-plurality groups | 1.75 | 1.00 |

Table S22a. Nationally representative descriptive statistics for United States

| **Characteristic** | **N = 38,312**^1^ |
| --- | --- |
| **Relationship with mother** |  |
| Very good | 20,590 (54%) |
| Somewhat good | 11,525 (30%) |
| Somewhat bad | 3,523 (9.2%) |
| Very bad | 1,874 (4.9%) |
| Does not apply | 694 (1.8%) |
| (Missing) | 106 (0.3%) |
| **Relationship with father** |  |
| Very good | 15,313 (40%) |
| Somewhat good | 12,665 (33%) |
| Somewhat bad | 4,879 (13%) |
| Very bad | 2,604 (6.8%) |
| Does not apply | 2,811 (7.3%) |
| (Missing) | 38 (0.1%) |
| **Parent marital status** |  |
| Parents married | 27,415 (72%) |
| Divorced | 6,325 (17%) |
| Parents were never married | 3,048 (8.0%) |
| One or both parents had died | 1,024 (2.7%) |
| (Missing) | 500 (1.3%) |
| **Subjective financial status of family growing up** |  |
| Lived comfortably | 15,116 (39%) |
| Got by | 15,682 (41%) |
| Found it difficult | 5,152 (13%) |
| Found it very difficult | 2,342 (6.1%) |
| (Missing) | 19 (<0.1%) |
| **Abuse** |  |
| Yes | 10,026 (26%) |
| No | 28,045 (73%) |
| (Missing) | 242 (0.6%) |
| **Outsider growing up** |  |
| Yes | 10,185 (27%) |
| No | 27,714 (72%) |
| (Missing) | 413 (1.1%) |
| **Self-rated health growing up** |  |
| Excellent | 16,866 (44%) |
| Very good | 12,108 (32%) |
| Good | 6,444 (17%) |
| Fair | 2,303 (6.0%) |
| Poor | 520 (1.4%) |
| (Missing) | 71 (0.2%) |
| **Immigration status** |  |
| Born in this country | 34,865 (91%) |
| Born in another country | 3,020 (7.9%) |
| (Missing) | 427 (1.1%) |
| **Age 12 religious service attendance** |  |
| At least 1/week | 18,609 (49%) |
| 1-3/month | 6,644 (17%) |
| <1/month | 5,829 (15%) |
| Never | 7,085 (18%) |
| (Missing) | 145 (0.4%) |
| **Year of birth** |  |
| 1998-2005; age 18-24 | 2,682 (7.0%) |
| 1993-1998; age 25-29 | 3,540 (9.2%) |
| 1983-1993; age 30-39 | 7,284 (19%) |
| 1973-1983; age 40-49 | 5,649 (15%) |
| 1963-1973; age 50-59 | 6,745 (18%) |
| 1953-1963; age 60-69 | 6,832 (18%) |
| 1943-1953; age 70-79 | 4,054 (11%) |
| 1943 or earlier; age 80+ | 1,525 (4.0%) |
| (Missing) | 0 (0%) |
| **Gender** |  |
| Male | 18,222 (48%) |
| Female | 19,562 (51%) |
| Other | 392 (1.0%) |
| (Missing) | 136 (0.4%) |
| **Religious affiliation** |  |
| Christianity | 30,444 (79%) |
| Islam | 220 (0.6%) |
| Hinduism | 203 (0.5%) |
| Buddhism | 172 (0.4%) |
| Judaism | 787 (2.1%) |
| Sikhism | 47 (0.1%) |
| Baha'i | 4 (<0.1%) |
| Jainism | 18 (<0.1%) |
| Shinto | 6 (<0.1%) |
| Taoism | 17 (<0.1%) |
| Confucianism | 8 (<0.1%) |
| Primal, Animist, or Folk religion | 67 (0.2%) |
| Spiritism | 0 (0%) |
| Umbanda, Candomble, and other African-derived religions | 0 (0%) |
| Chinese folk/traditional religion | 0 (0%) |
| Some other religion | 359 (0.9%) |
| No religion/Atheist/Agnostic | 5,845 (15%) |
| (Missing) | 115 (0.3%) |
| **Race/Ethnicity** |  |
| Asian | 2,466 (6.4%) |
| Black | 4,501 (12%) |
| Hispanic | 6,724 (18%) |
| Other | 997 (2.6%) |
| White | 23,605 (62%) |
| (Missing) | 20 (<0.1%) |
| ^1^n (%) | |

Table S22b. Childhood predictors regression for United States

| Variable | Category | Risk-Ratio | RR 95% CI | Global p-value |
| --- | --- | --- | --- | --- |
| Relationship with mother | (Ref: Very bad/somewhat bad) |  |  | 0.919 |
|  | Very good/somewhat good | 1.00 | (0.89,1.11) |  |
| Relationship with father | (Ref: Very bad/somewhat bad) |  |  | 0.025 |
|  | Very good/somewhat good | 0.89 | (0.80,0.99) |  |
| Parent marital status | (Ref: Parents married) |  |  | 0.552 |
|  | Divorced | 0.99 | (0.88,1.12) |  |
|  | Parents were never married | 1.14 | (0.92,1.41) |  |
|  | One or both parents had died | 0.96 | (0.76,1.21) |  |
| Subjective financial status of family growing up | (Ref: Got by) |  |  | 0.043 |
|  | Lived comfortably | 0.95 | (0.87,1.03) |  |
|  | Found it difficult | 1.05 | (0.94,1.18) |  |
|  | Found it very difficult | 1.24 | (1.03,1.49) |  |
| Abuse | (Ref: No) |  |  | <.001 |
|  | Yes | 1.37 | (1.25,1.49) |  |
| Outsider growing up | (Ref: No) |  |  | <.001 |
|  | Yes | 1.28 | (1.16,1.41) |  |
| Self-rated health growing up | (Ref: Good) |  |  | <.001 |
|  | Excellent | 0.51 | (0.46,0.57) |  |
|  | Very good | 0.70 | (0.63,0.78) |  |
|  | Fair | 1.29 | (1.10,1.52) |  |
|  | Poor | 1.64 | (1.35,1.99) |  |
| Immigration status | (Ref: Born in this country) |  |  | 0.002 |
|  | Born in another country | 0.63 | (0.48,0.84) |  |
| Age 12 religious service attendance | (Ref: Never) |  |  | 0.503 |
|  | At least 1/week | 0.94 | (0.83,1.06) |  |
|  | 1-3/month | 0.89 | (0.77,1.04) |  |
|  | < 1/month | 0.92 | (0.80,1.07) |  |
| Year of birth | (Ref: 1998-2005; current age: 18-24) |  |  | <.001 |
|  | 1993-1998; age 25-29 | 1.15 | (0.80,1.65) |  |
|  | 1983-1993; age 30-39 | 0.96 | (0.68,1.36) |  |
|  | 1973-1983; age 40-49 | 1.26 | (0.90,1.76) |  |
|  | 1963-1973; age 50-59 | 1.68 | (1.21,2.33) |  |
|  | 1953-1963; age 60-69 | 1.90 | (1.37,2.62) |  |
|  | 1943-1953; age 70-79 | 2.16 | (1.56,2.99) |  |
|  | 1943 or earlier; age 80+ | 2.64 | (1.89,3.68) |  |
| Gender | (Ref: Male) |  |  | 0.035 |
|  | Female | 1.10 | (1.01,1.19) |  |
|  | Other | 1.31 | (0.93,1.84) |  |
| Religious affiliation | (Ref: No religion/Atheist/Agnostic) |  |  | 0.117 |
|  | Christianity | 1.14 | (0.99,1.31) |  |
|  | Collapsed affiliations with prevalence<3% | 1.23 | (0.99,1.51) |  |
| Race/ethnicity | (Ref: Plurality group) |  |  | 0.117 |
|  | Non-plurality groups | 0.93 | (0.84,1.02) |  |

Table S22c. Sensitivity to unmeasured confounding of childhood predictors in United States

| Variable | Category | E-value for Estimate | E-value for 95% CI |
| --- | --- | --- | --- |
| Relationship with mother | (Ref: Very bad/somewhat bad) |  |  |
|  | Very good/somewhat good | 1.08 | 1.00 |
| Relationship with father | (Ref: Very bad/somewhat bad) |  |  |
|  | Very good/somewhat good | 1.51 | 1.14 |
| Parent marital status | (Ref: Parents married) |  |  |
|  | Divorced | 1.10 | 1.00 |
|  | Parents were never married | 1.54 | 1.00 |
|  | One or both parents had died | 1.25 | 1.00 |
| Subjective financial status of family growing up | (Ref: Got by) |  |  |
|  | Lived comfortably | 1.30 | 1.00 |
|  | Found it difficult | 1.28 | 1.00 |
|  | Found it very difficult | 1.78 | 1.21 |
| Abuse | (Ref: No) |  |  |
|  | Yes | 2.07 | 1.81 |
| Outsider growing up | (Ref: No) |  |  |
|  | Yes | 1.87 | 1.59 |
| Self-rated health growing up | (Ref: Good) |  |  |
|  | Excellent | 3.30 | 2.87 |
|  | Very good | 2.21 | 1.88 |
|  | Fair | 1.90 | 1.43 |
|  | Poor | 2.66 | 2.04 |
| Immigration status | (Ref: Born in this country) |  |  |
|  | Born in another country | 2.54 | 1.66 |
| Age 12 religious service attendance | (Ref: Never) |  |  |
|  | At least 1/week | 1.34 | 1.00 |
|  | 1-3/month | 1.49 | 1.00 |
|  | < 1/month | 1.38 | 1.00 |
| Year of birth | (Ref: 1998-2005; current age: 18-24) |  |  |
|  | 1993-1998; age 25-29 | 1.56 | 1.00 |
|  | 1983-1993; age 30-39 | 1.23 | 1.00 |
|  | 1973-1983; age 40-49 | 1.83 | 1.00 |
|  | 1963-1973; age 50-59 | 2.74 | 1.70 |
|  | 1953-1963; age 60-69 | 3.20 | 2.09 |
|  | 1943-1953; age 70-79 | 3.75 | 2.50 |
|  | 1943 or earlier; age 80+ | 4.71 | 3.18 |
| Gender | (Ref: Male) |  |  |
|  | Female | 1.43 | 1.14 |
|  | Other | 1.95 | 1.00 |
| Religious affiliation | (Ref: No religion/Atheist/Agnostic) |  |  |
|  | Christianity | 1.53 | 1.00 |
|  | Collapsed affiliations with prevalence<3% | 1.75 | 1.00 |
| Race/ethnicity | (Ref: Plurality group) |  |  |
|  | Non-plurality groups | 1.37 | 1.00 |

Table S23. Population weighted meta-analysis of regression results and sensitivity of estimates to unmeasured confounding.

| Variable | Category | RR | 95% CI | E-value for Estimate | E-value for 95% CI |
| --- | --- | --- | --- | --- | --- |
| Relationship with mother | (Ref: Very bad/somewhat bad) |  |  |  |  |
|  | Very good/somewhat good | 1.06 | (0.96,1.18) | 1.32 | 1.00 |
| Relationship with father | (Ref: Very bad/somewhat bad) |  |  |  |  |
|  | Very good/somewhat good | 1.02 | (0.92,1.12) | 1.16 | 1.00 |
| Parent marital status | (Ref: Parents married) |  |  |  |  |
|  | Divorced | 1.10 | (0.99,1.23) | 1.44 | 1.00 |
|  | Single, never married | 0.98 | (0.89,1.07) | 1.18 | 1.00 |
|  | One or both parents had died | 1.03 | (0.96,1.10) | 1.20 | 1.00 |
| Subjective financial status of family growing up | (Ref: Got by) |  |  |  |  |
|  | Lived comfortably | 0.95 | (0.91,0.99) | 1.29 | 1.12 |
|  | Found it difficult | 1.01 | (0.96,1.06) | 1.10 | 1.00 |
|  | Found it very difficult | 1.10 | (1.03,1.17) | 1.42 | 1.20 |
| Abuse | (Ref: No) |  |  |  |  |
|  | Yes | 1.68 | (1.61,1.75) | 2.75 | 2.61 |
| Outsider growing up | (Ref: No) |  |  |  |  |
|  | Yes | 1.19 | (1.13,1.25) | 1.66 | 1.52 |
| Self-rated health growing up | (Ref: Good) |  |  |  |  |
|  | Excellent | 0.82 | (0.78,0.87) | 1.72 | 1.56 |
|  | Very good | 0.87 | (0.83,0.91) | 1.58 | 1.44 |
|  | Fair | 1.19 | (1.12,1.25) | 1.66 | 1.50 |
|  | Poor | 1.44 | (1.30,1.58) | 2.23 | 1.92 |
| Immigration status | (Ref: Born in this country) |  |  |  |  |
|  | Born in another country | 0.87 | (0.73,1.03) | 1.57 | 1.00 |
| Age 12 religious service attendance | (Ref: Never) |  |  |  |  |
|  | At least 1/week | 1.01 | (0.94,1.08) | 1.11 | 1.00 |
|  | 1-3/month | 1.06 | (0.98,1.14) | 1.30 | 1.00 |
|  | < 1/month | 1.04 | (0.96,1.12) | 1.24 | 1.00 |
| Year of birth | (Ref: 1998-2005; age 18-24) |  |  |  |  |
|  | 1993-1998; age 25-29 | 1.06 | (0.98,1.16) | 1.32 | 1.00 |
|  | 1983-1993; age 30-39 | 1.17 | (1.09,1.26) | 1.62 | 1.39 |
|  | 1973-1983; age 40-49 | 1.57 | (1.47,1.69) | 2.53 | 2.29 |
|  | 1963-1973; age 50-59 | 1.94 | (1.80,2.09) | 3.29 | 3.00 |
|  | 1953-1963; age 60-69 | 2.40 | (2.22,2.60) | 4.24 | 3.87 |
|  | 1943-1953; age 70-79 | 2.75 | (2.50,3.02) | 4.94 | 4.43 |
|  | 1943 or earlier; age 80+ | 1.95 | (1.31,2.92) | 3.31 | 1.94 |
| Gender | (Ref: Male) |  |  |  |  |
|  | Female | 1.14 | (1.11,1.18) | 1.55 | 1.45 |
|  | Other | 0.32 | (0.20,0.51) | 5.70 | 3.35 |


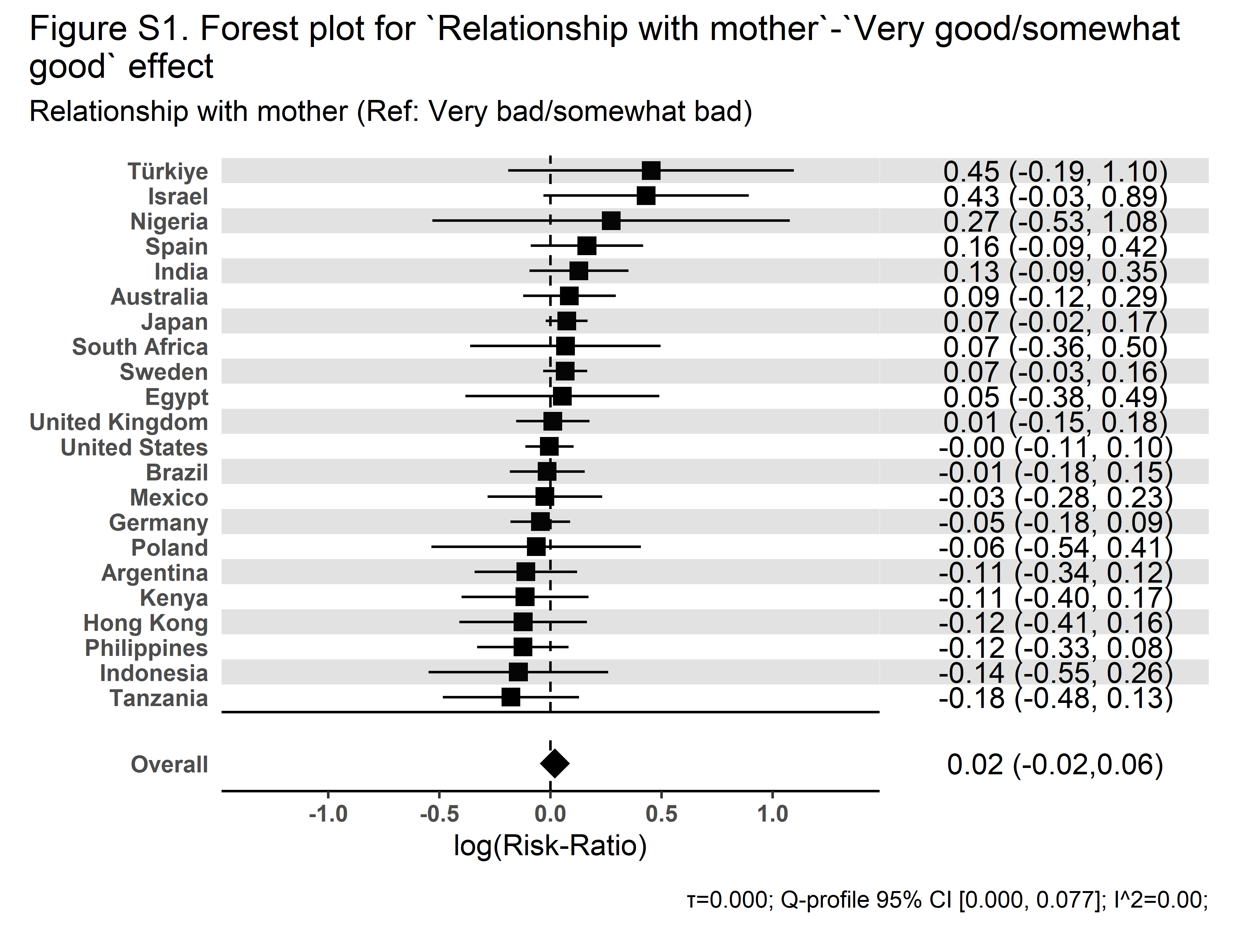

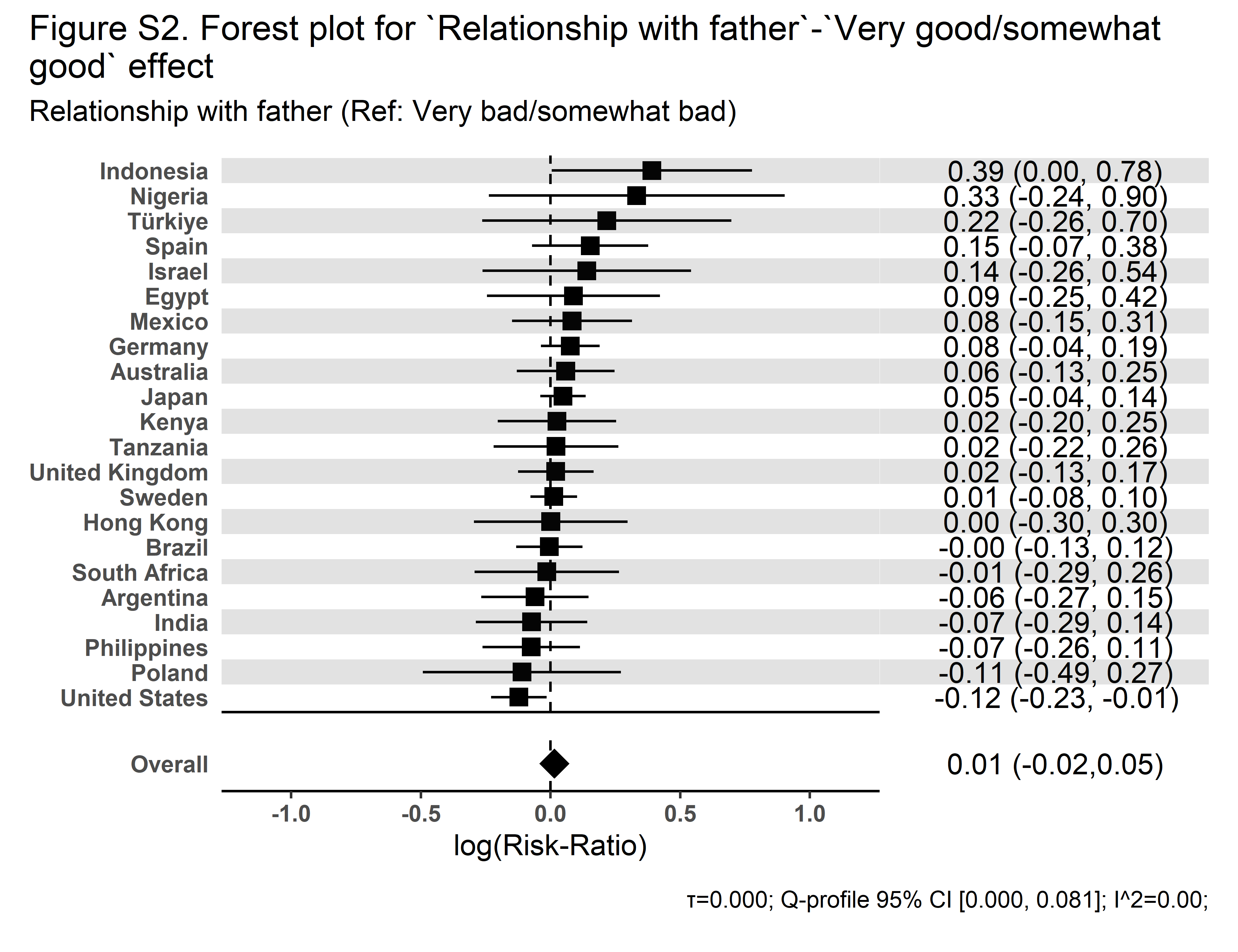

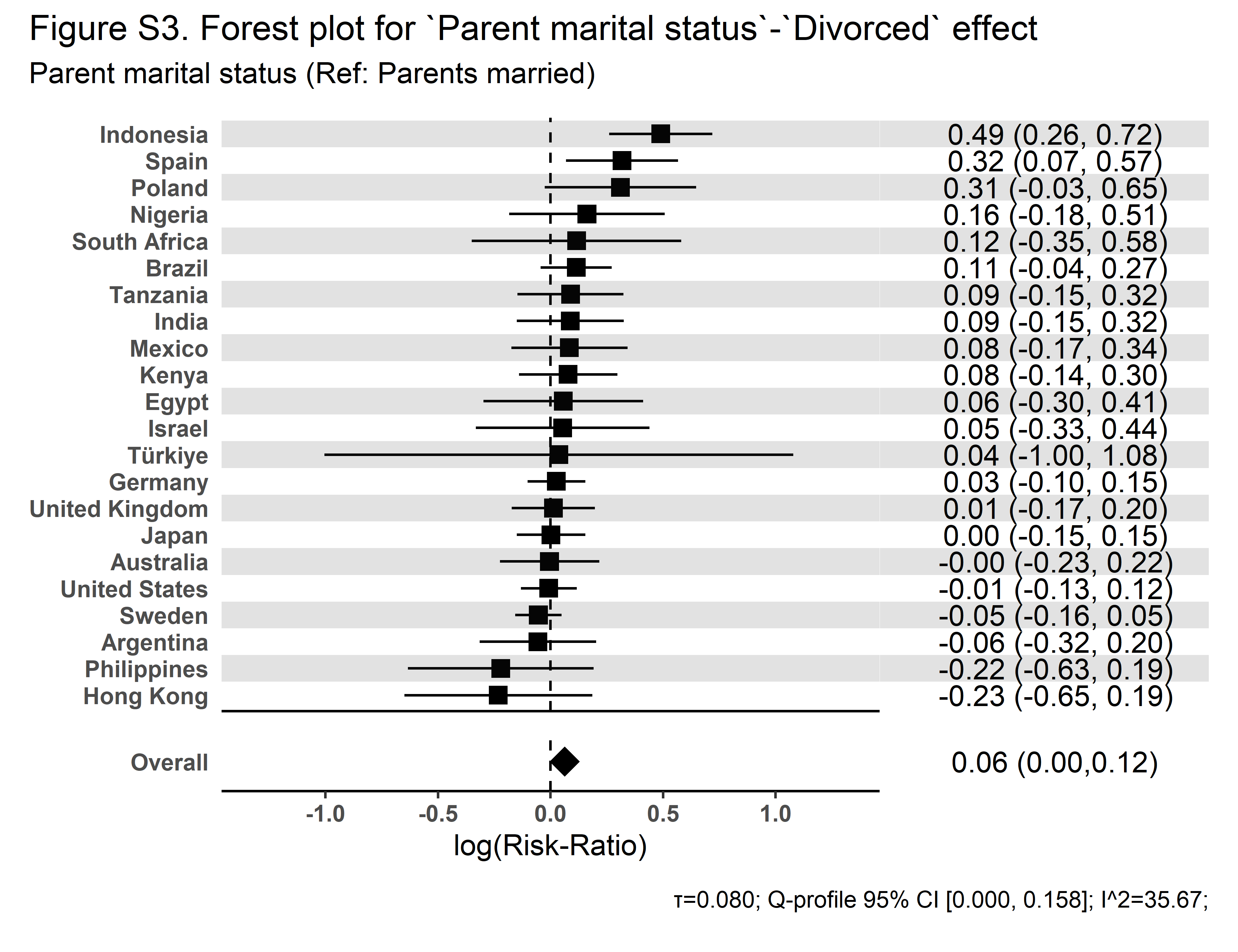

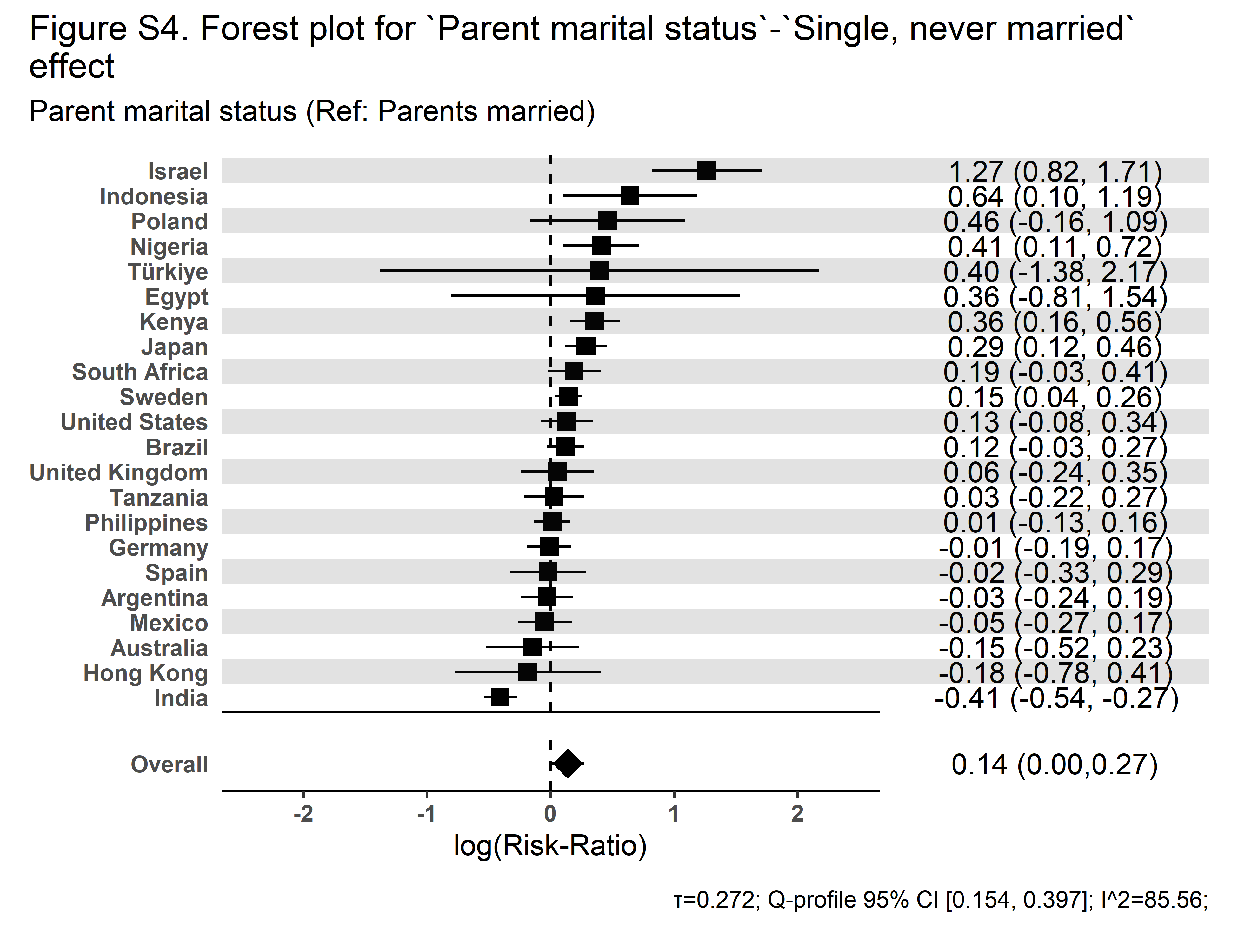

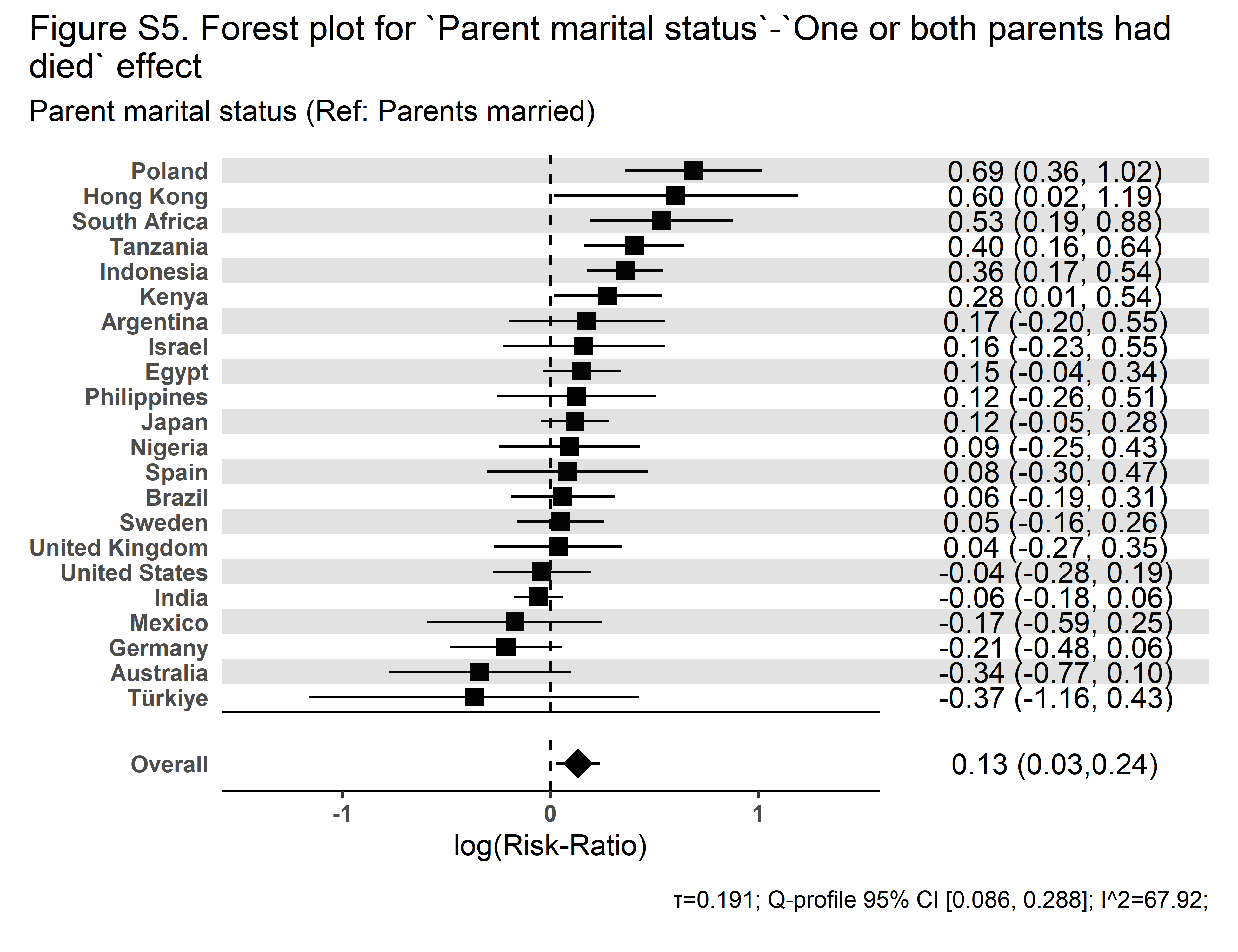

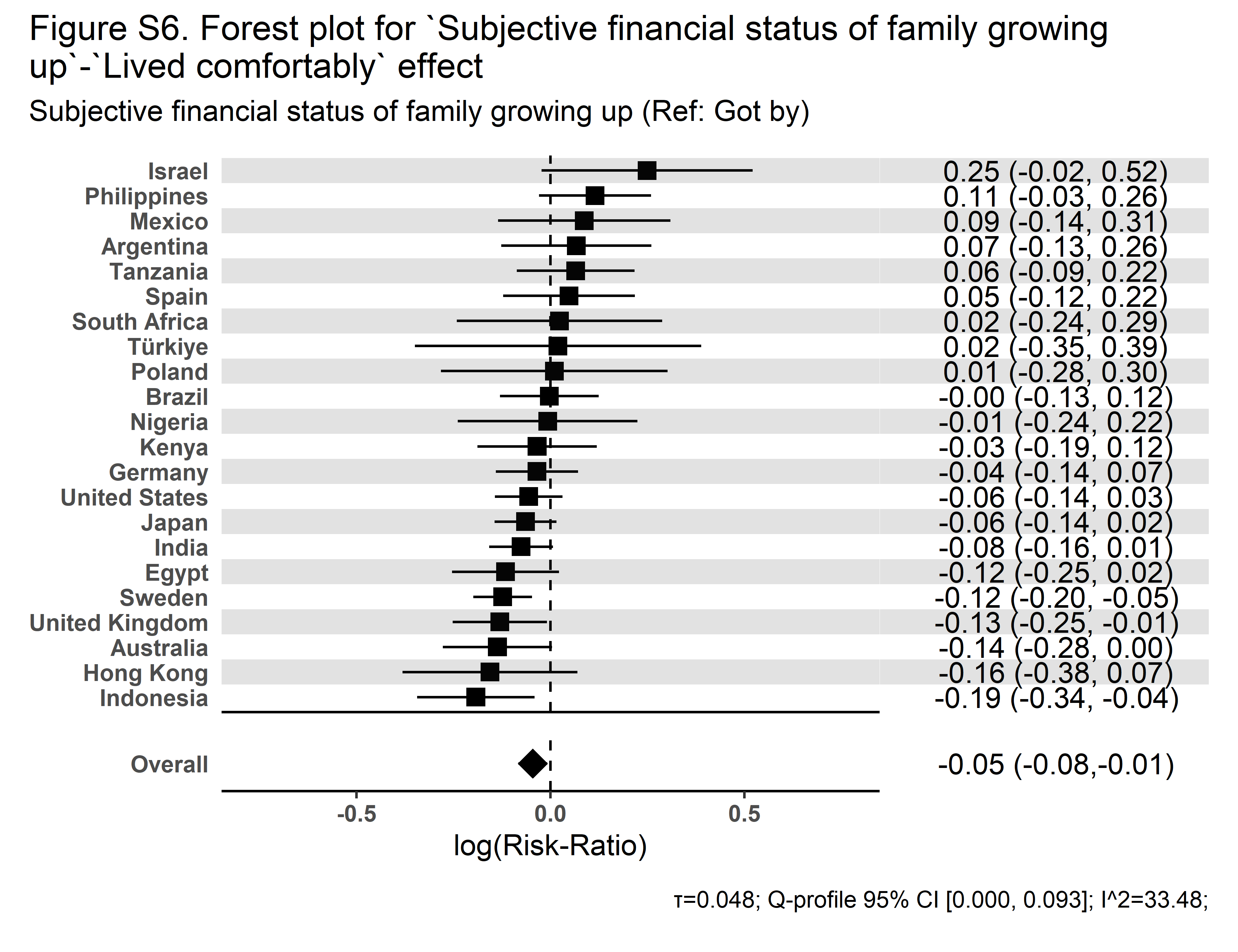

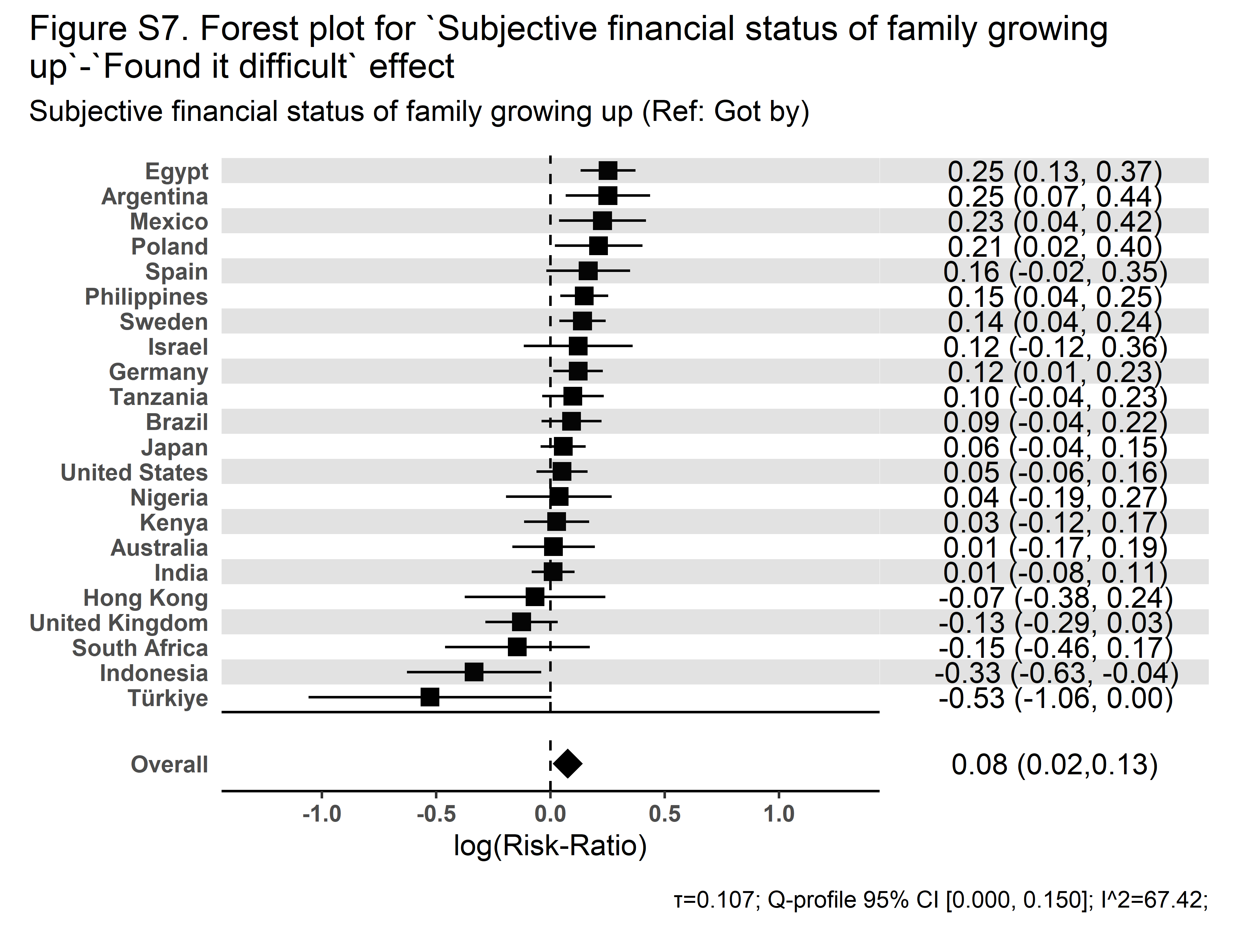

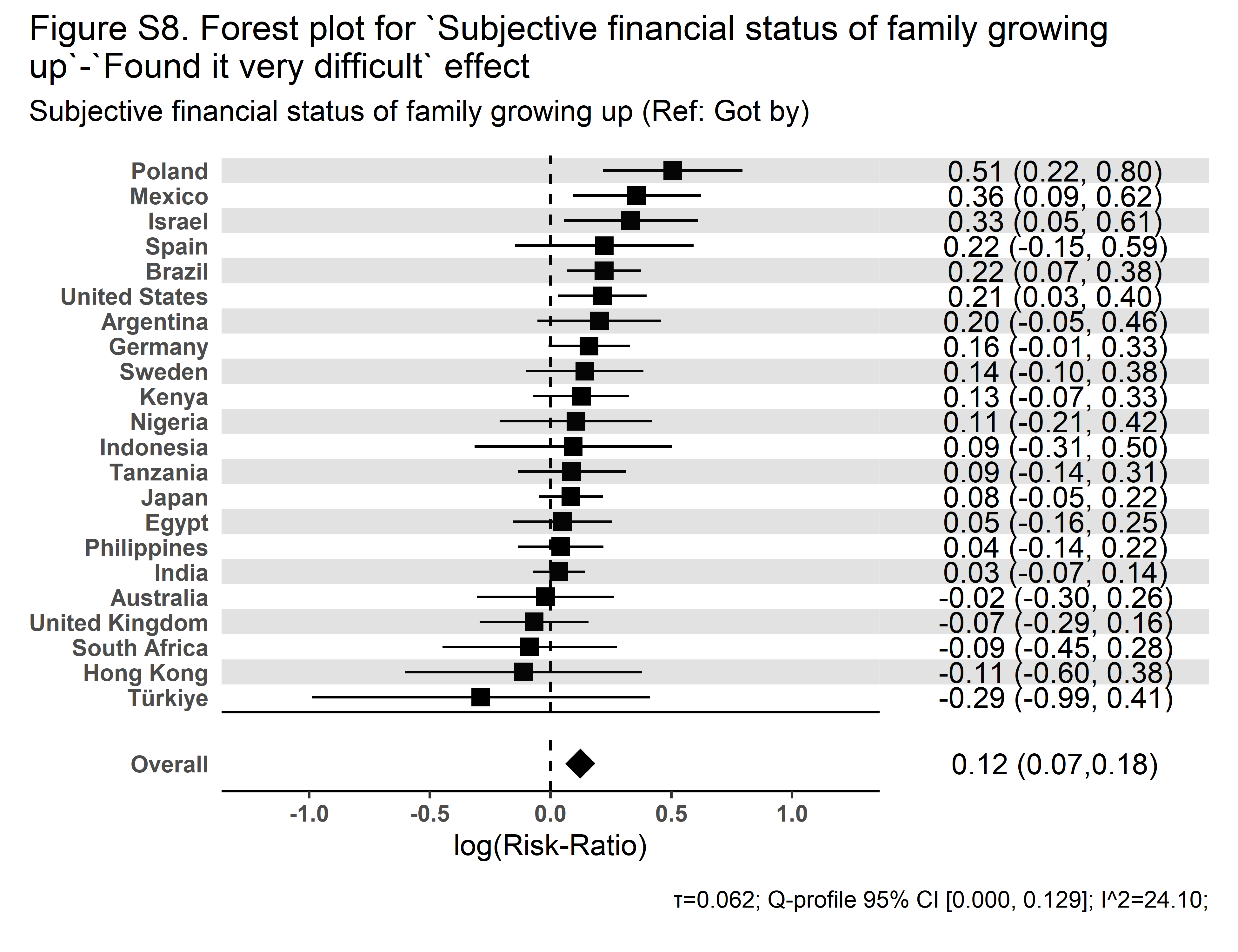

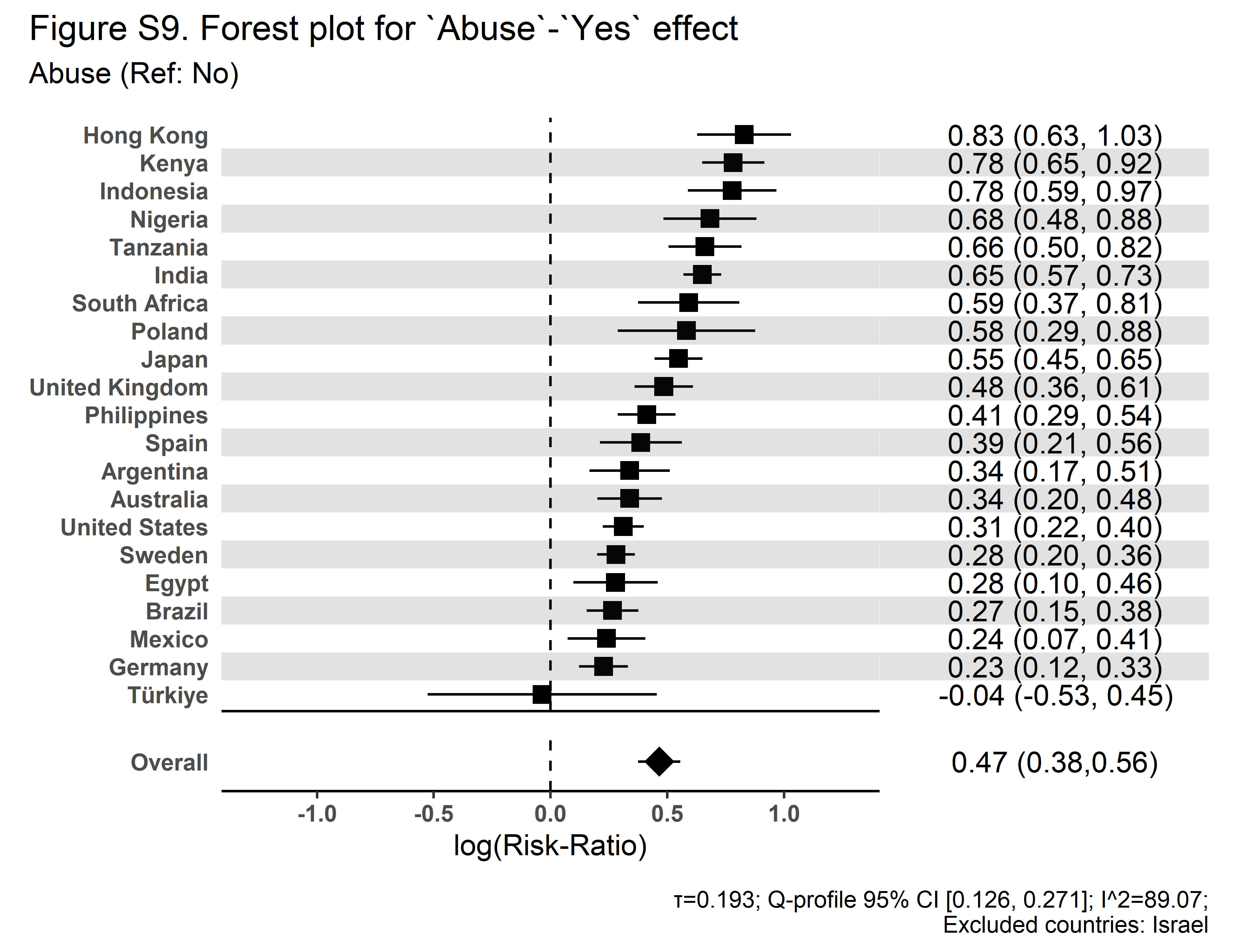

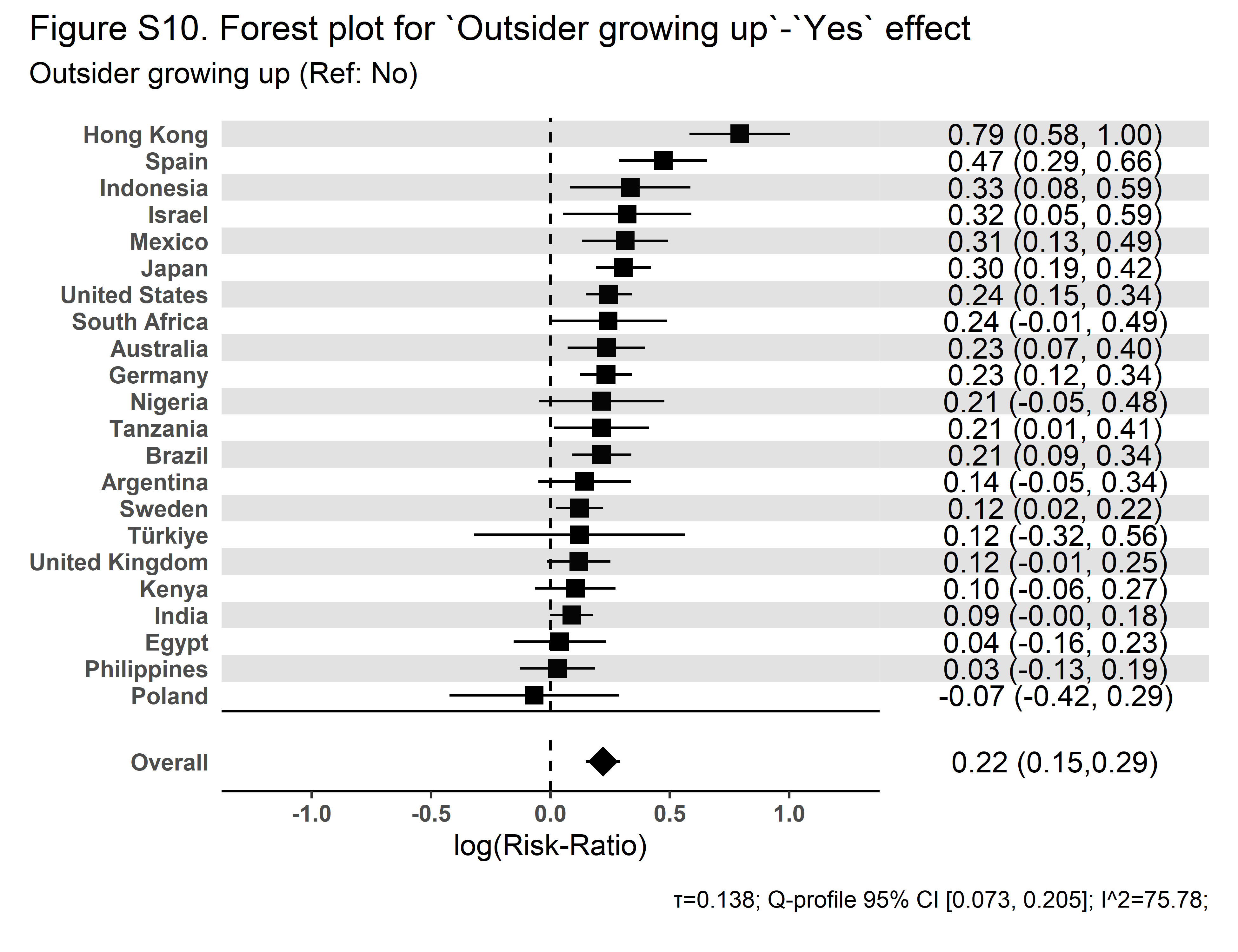

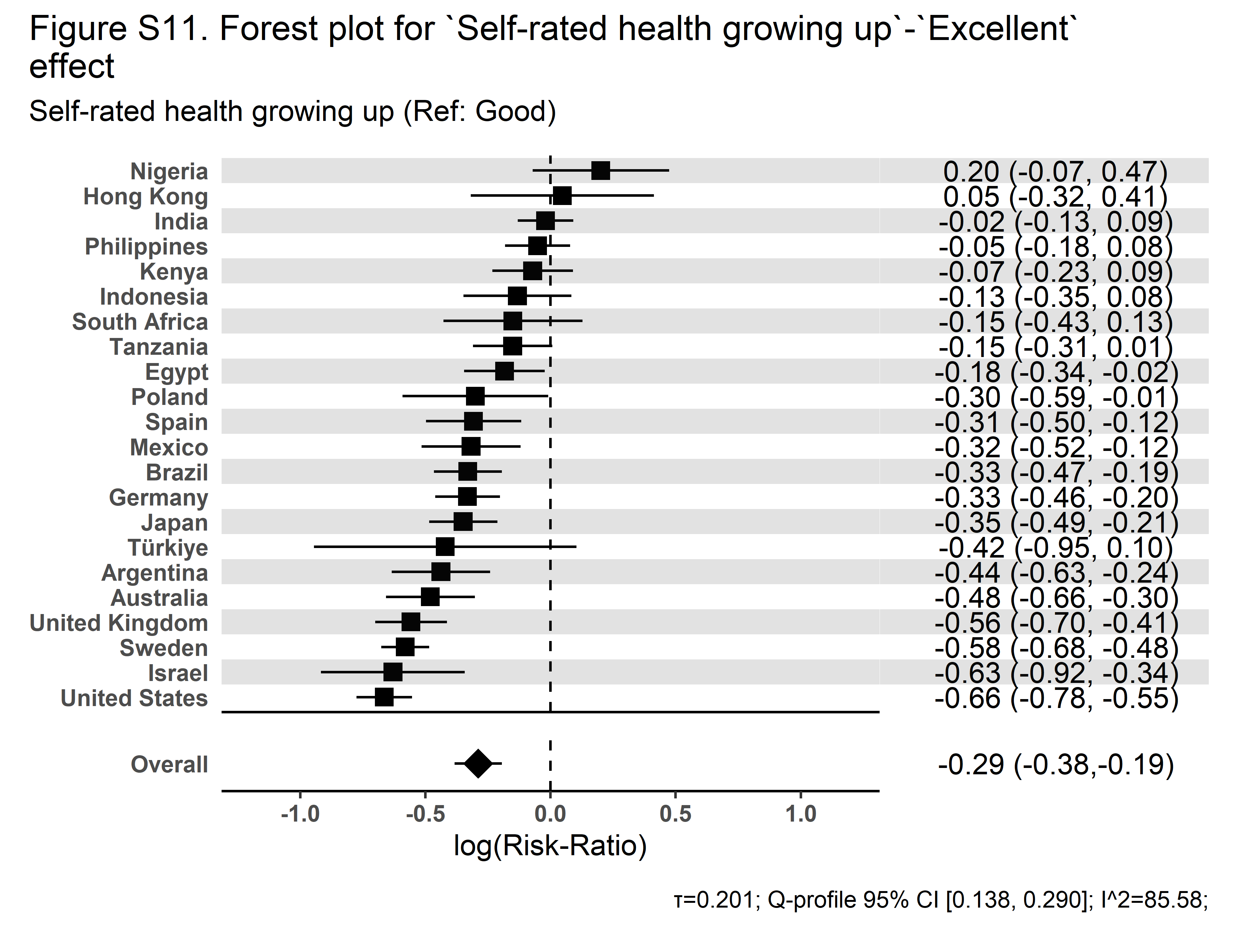

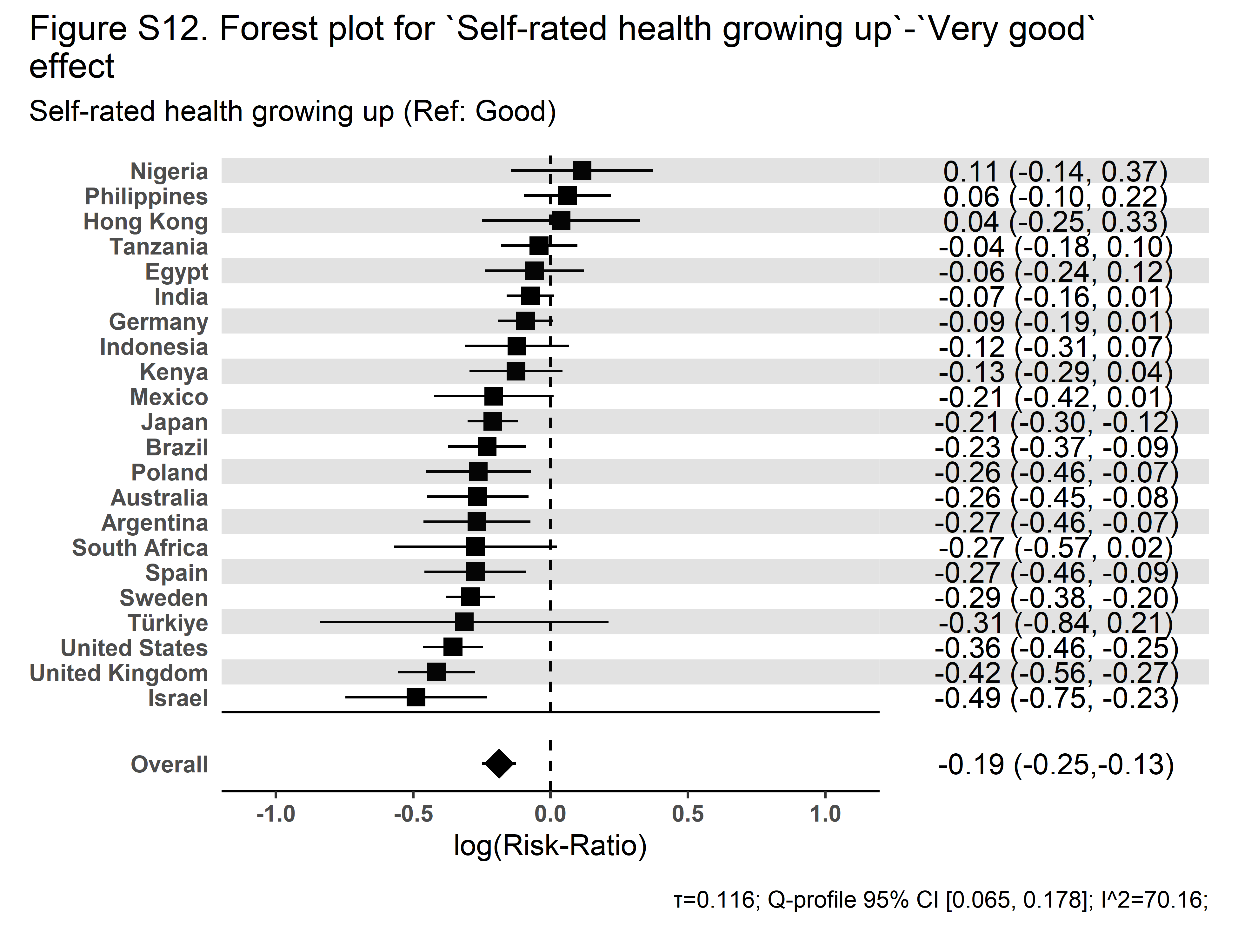

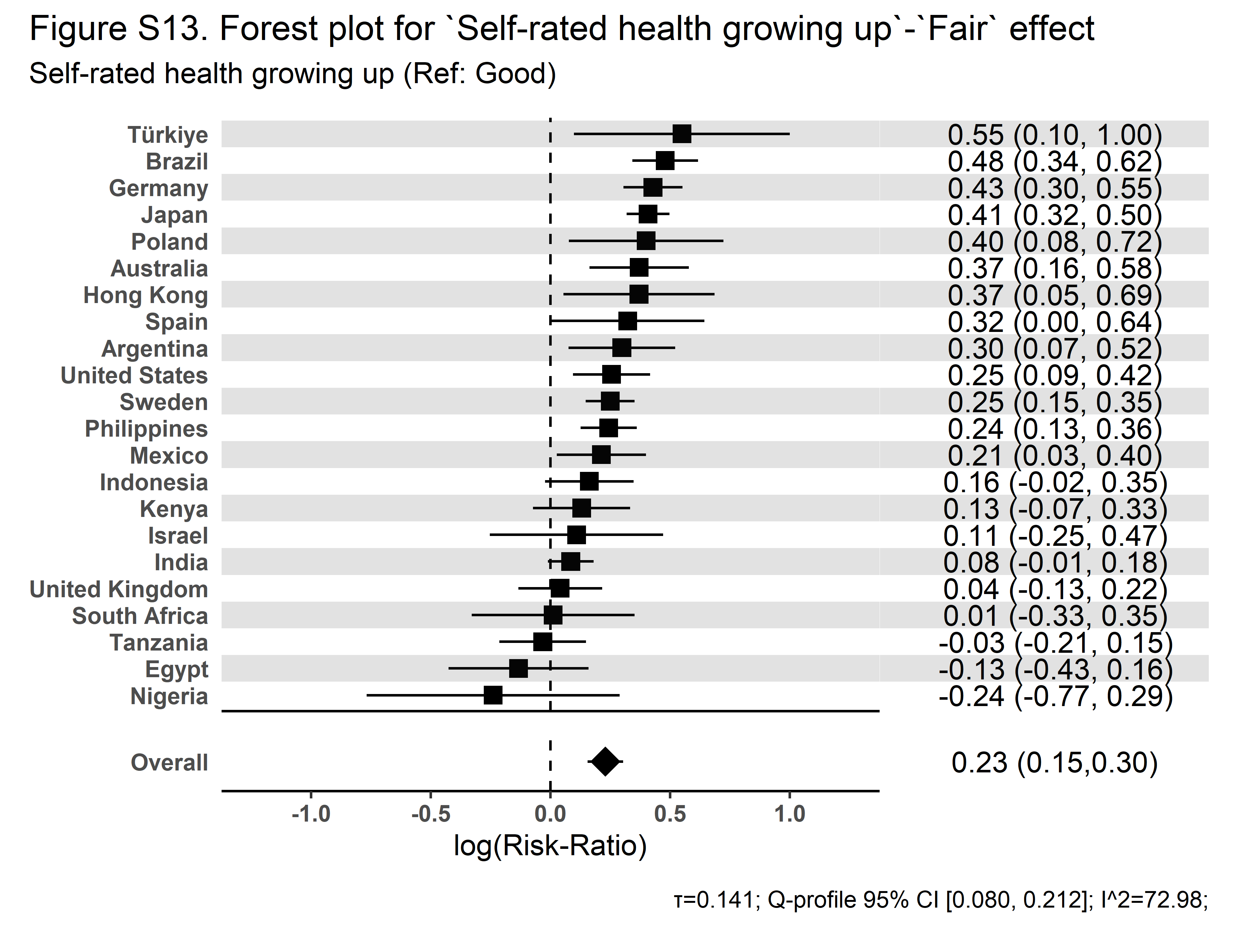

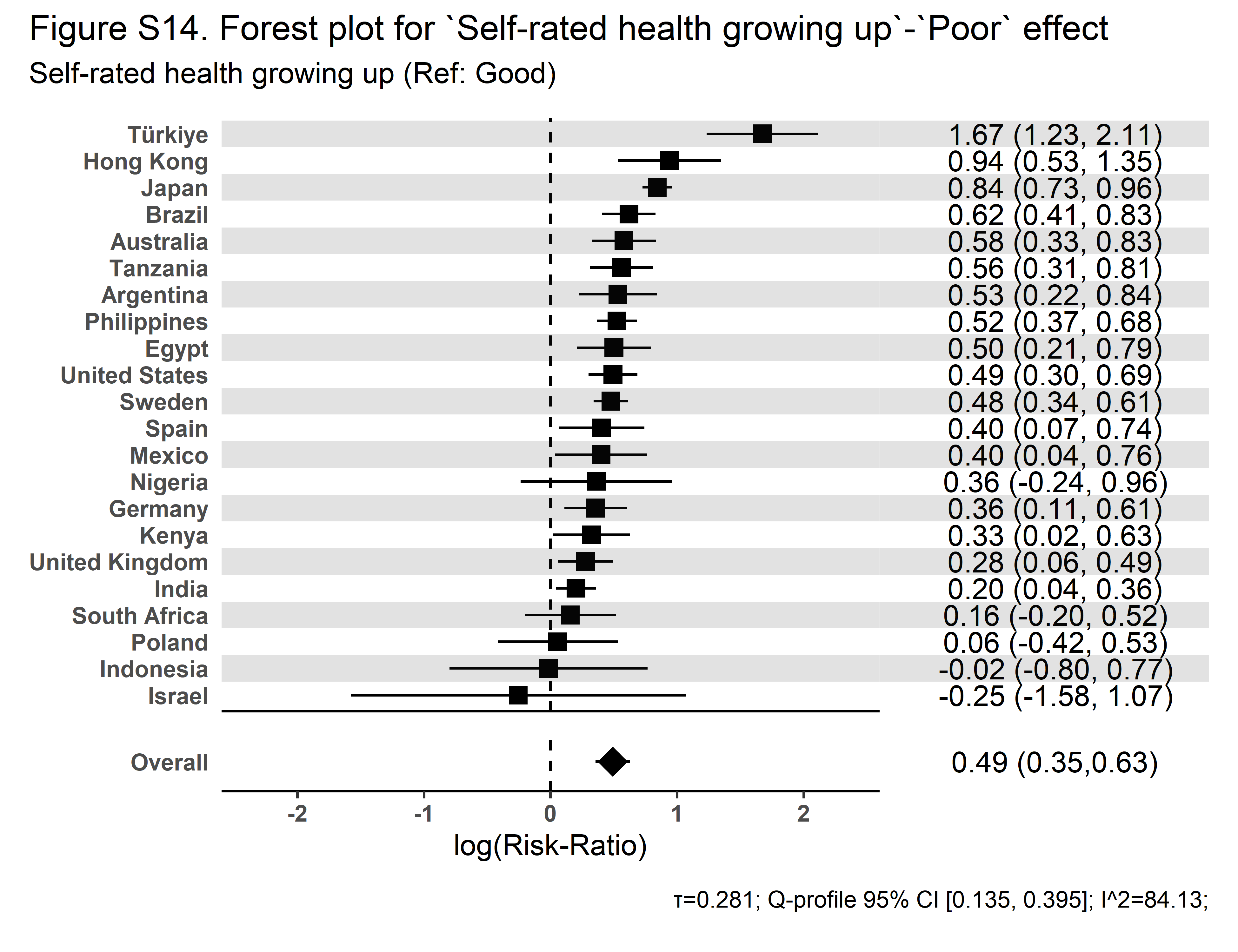

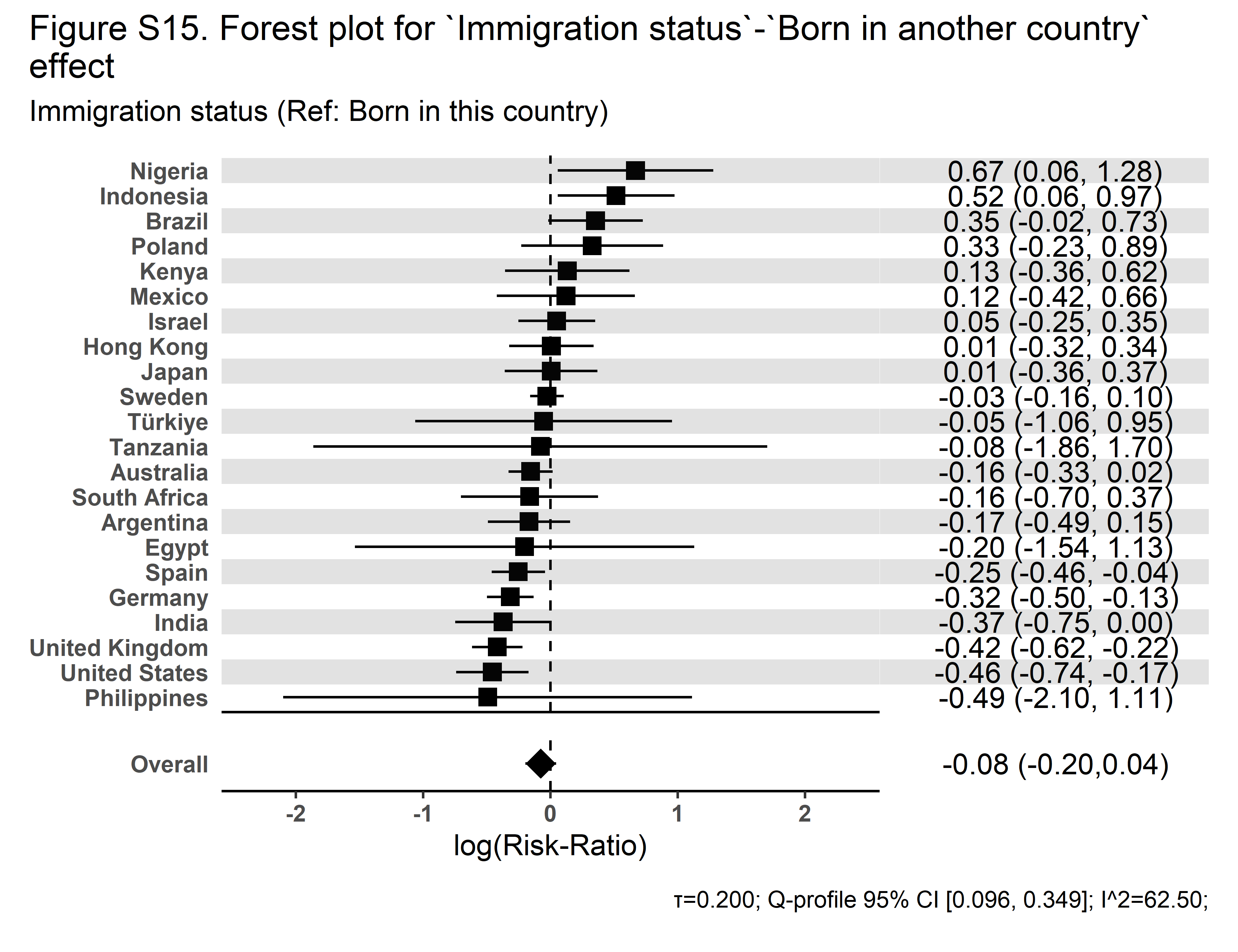

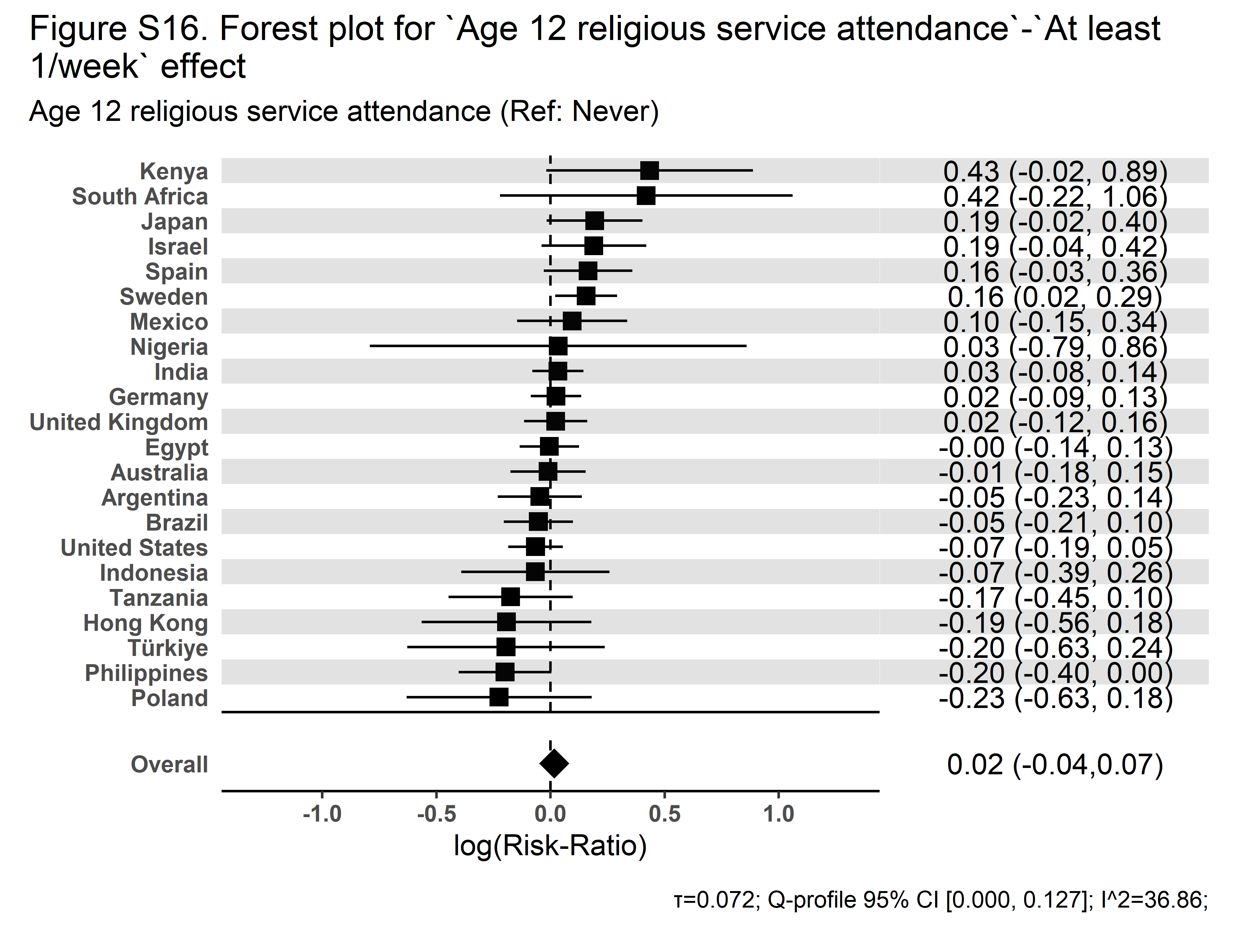

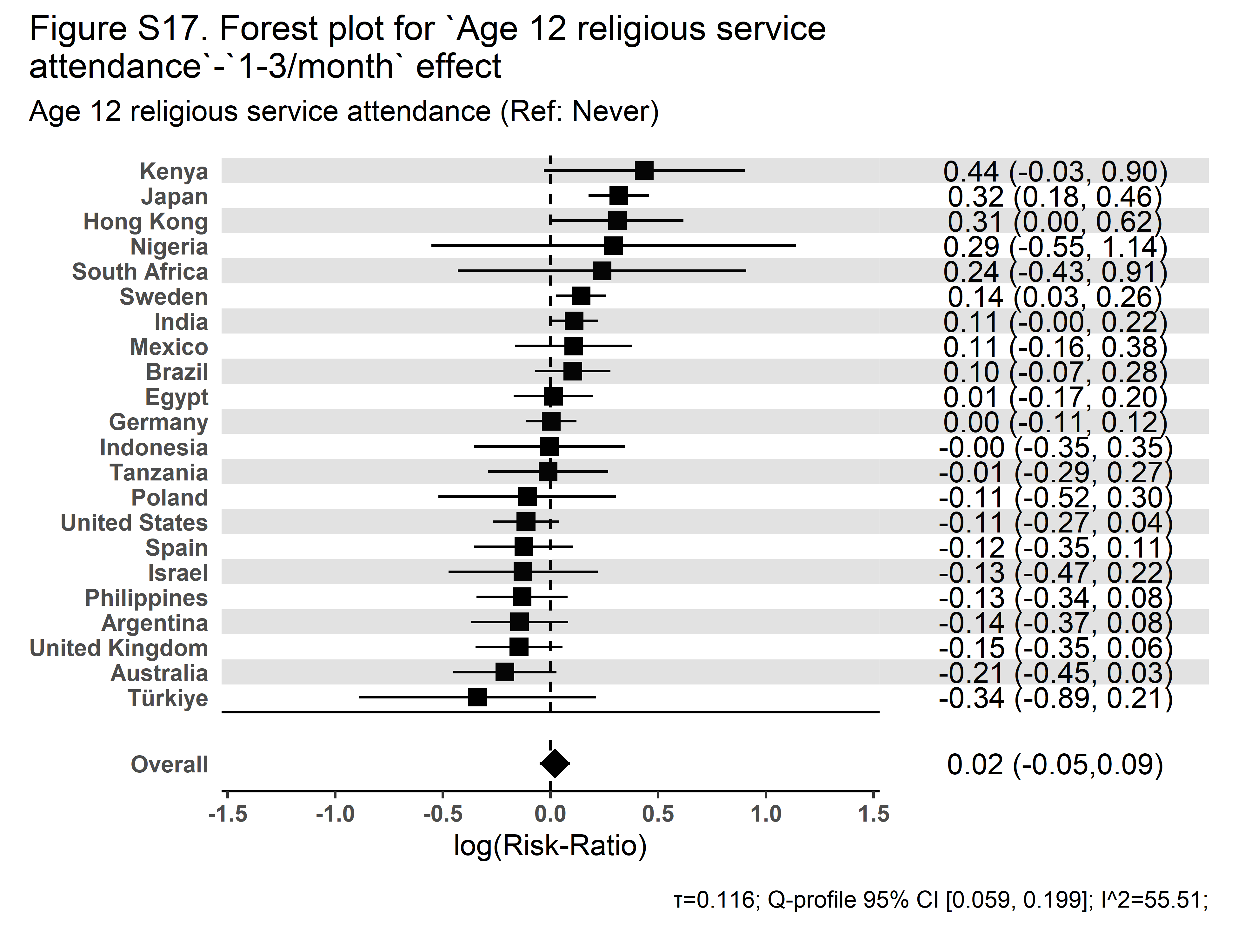

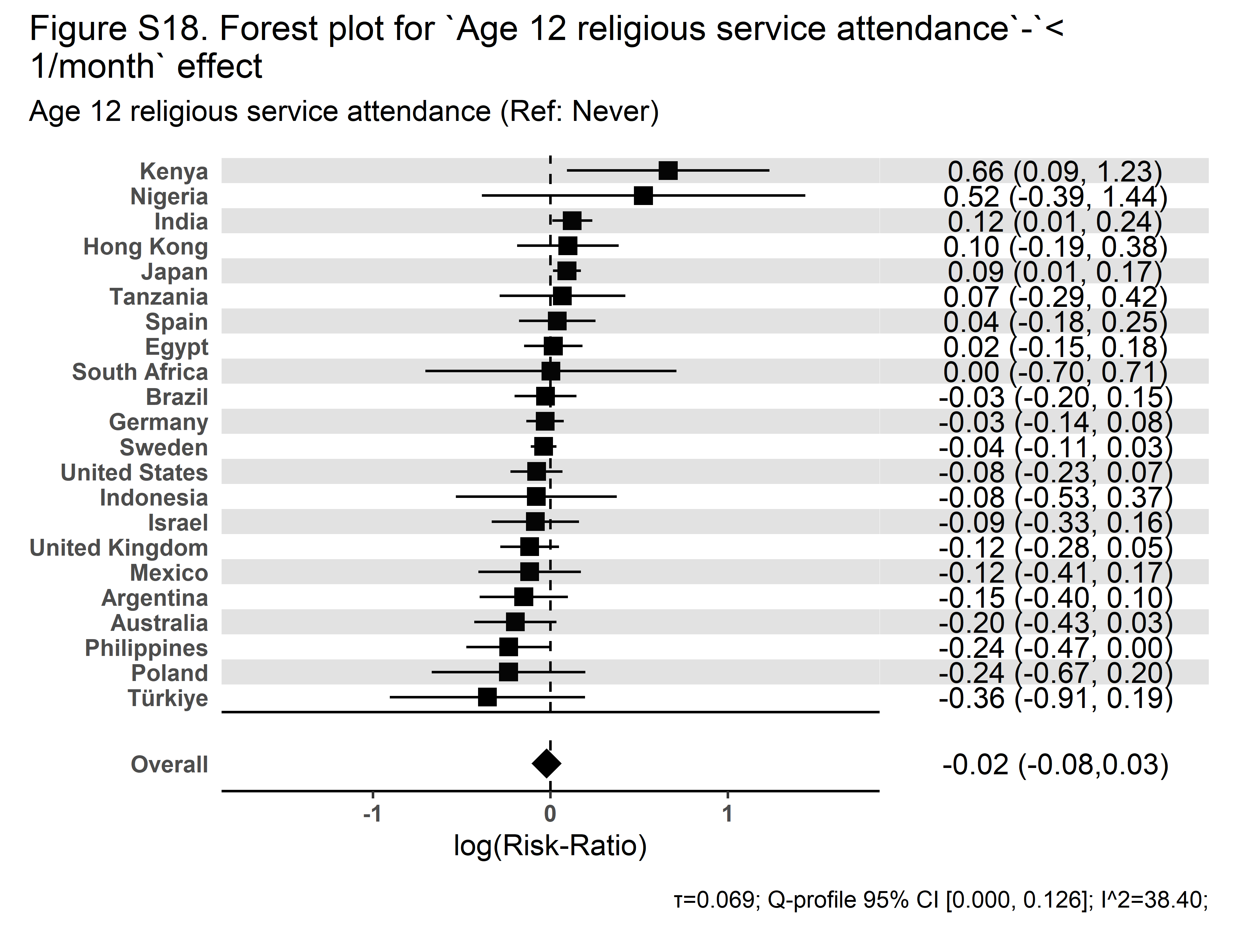

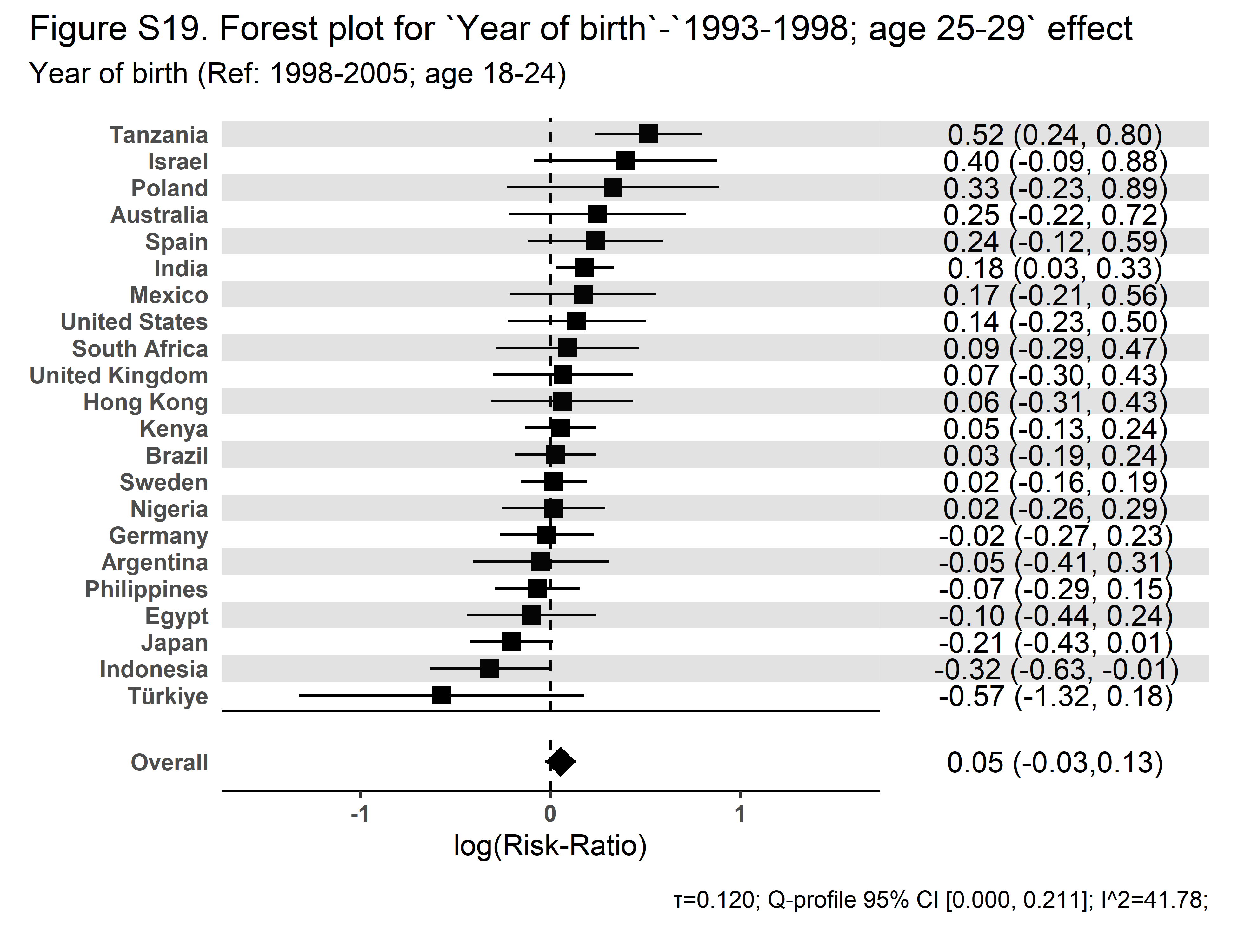

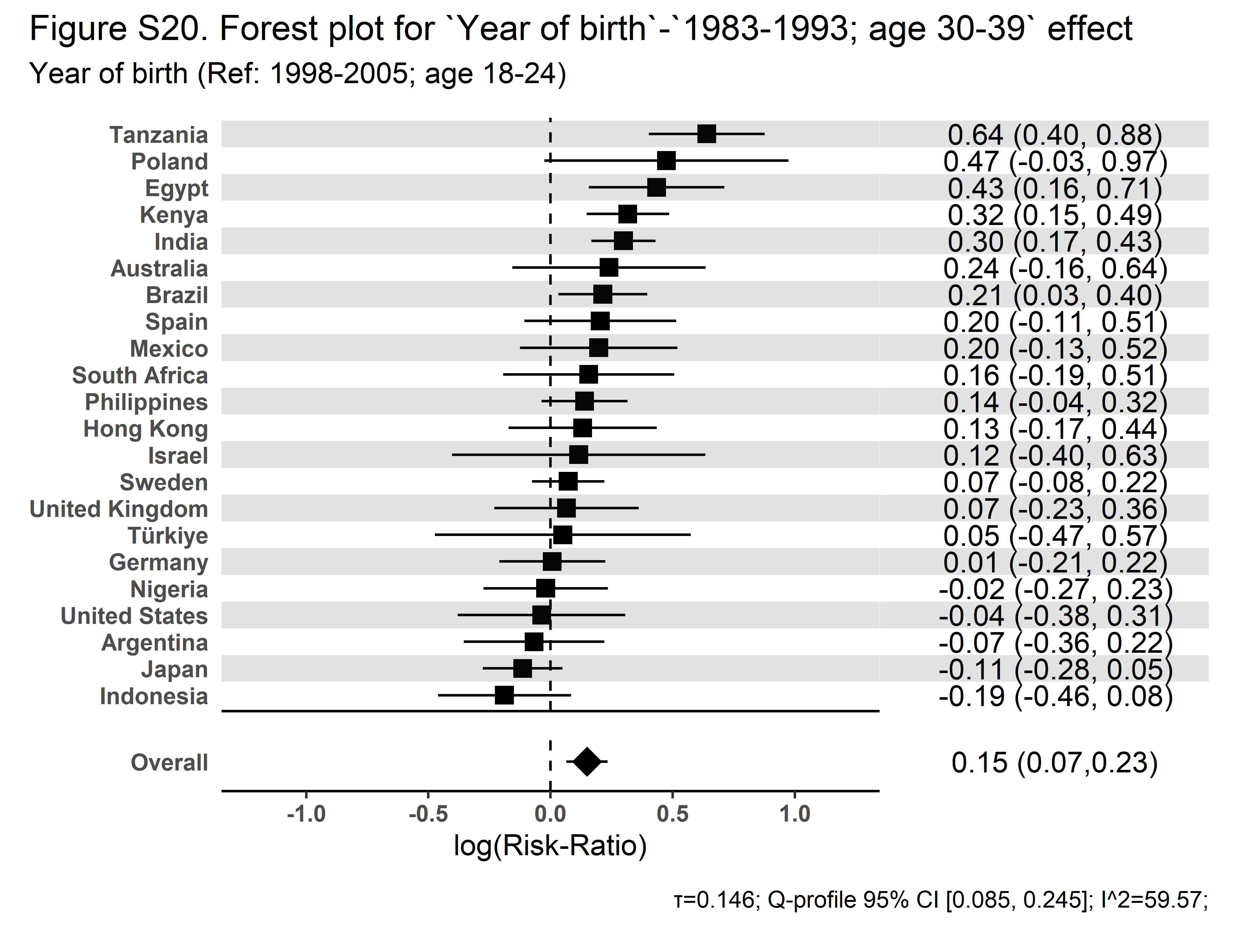

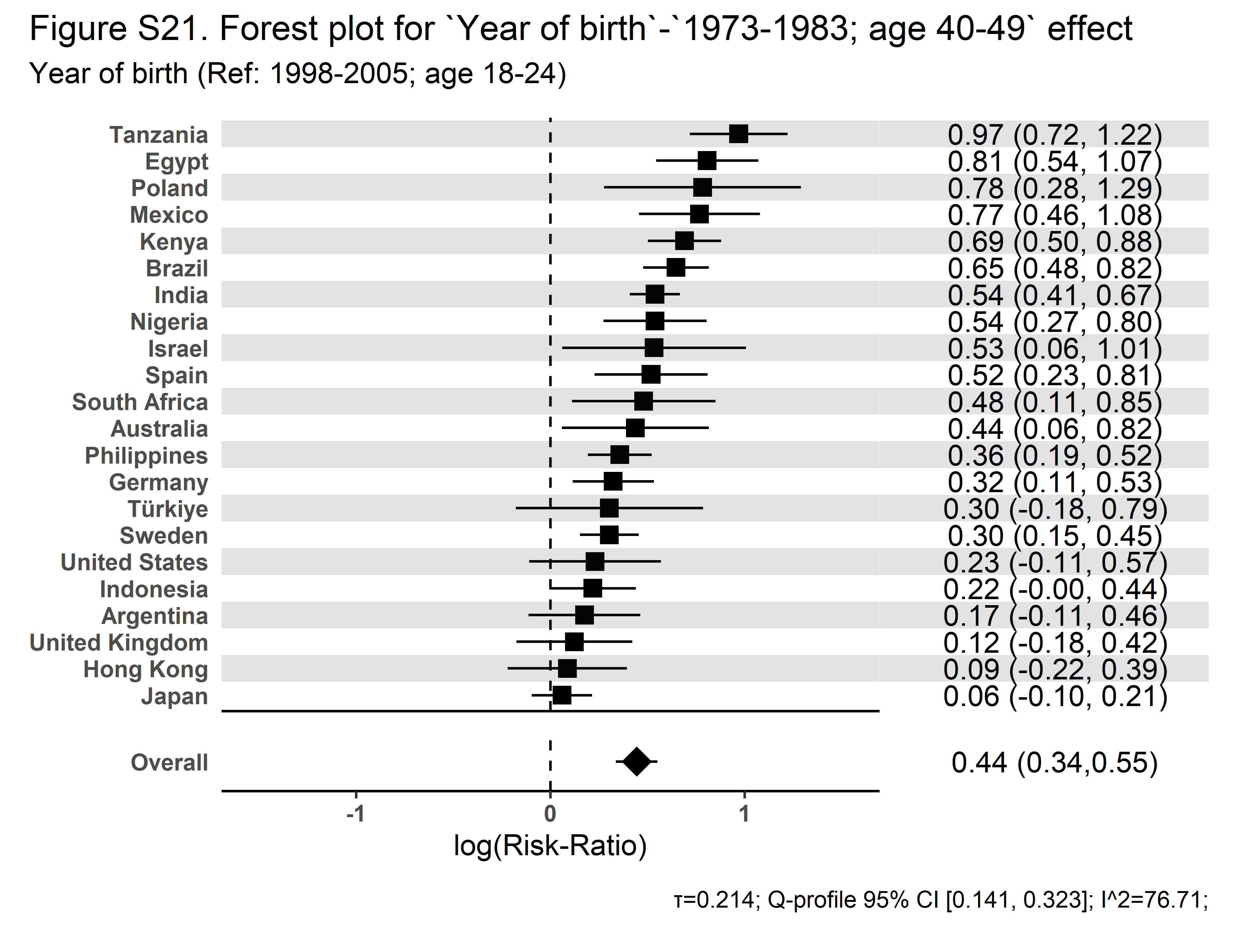

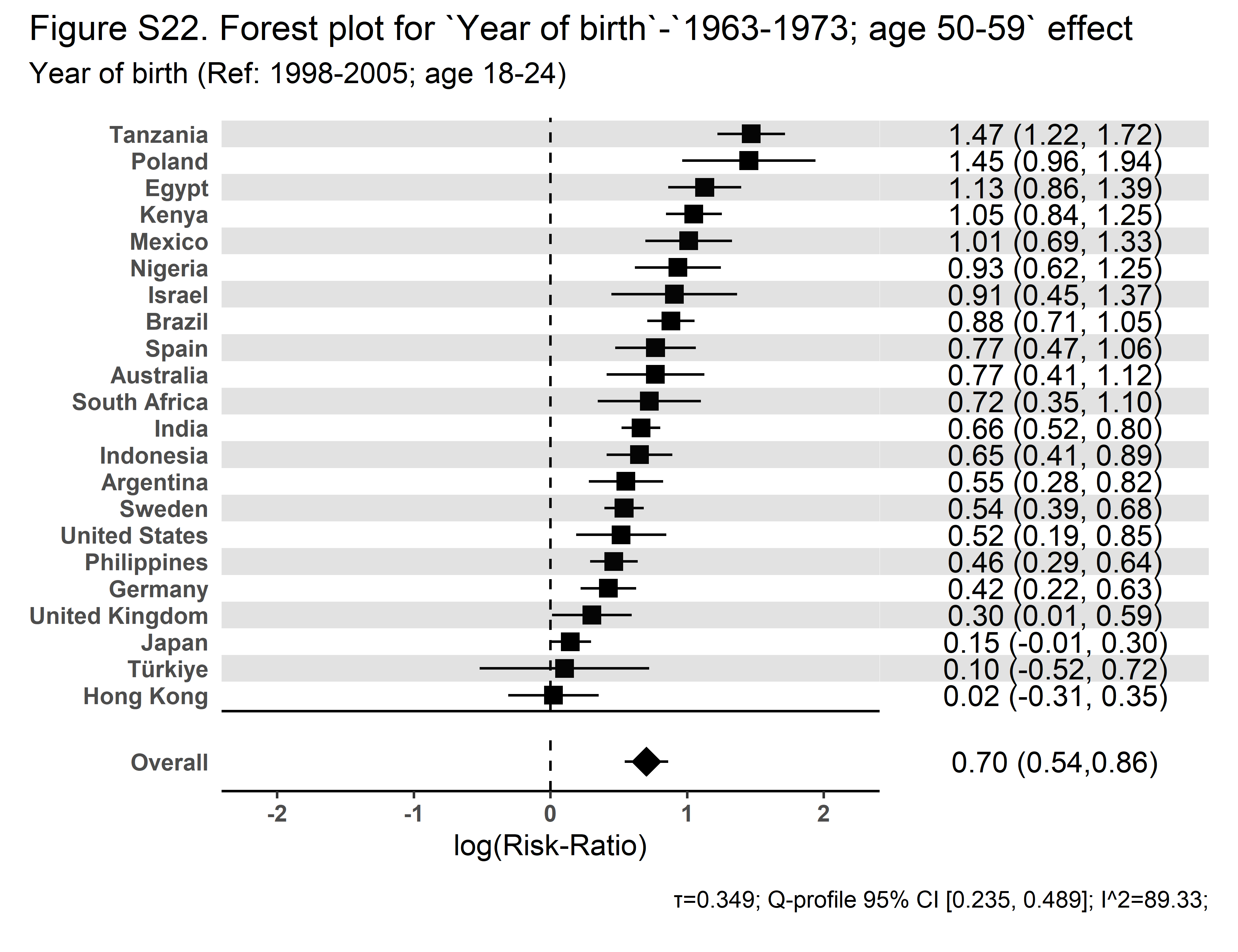

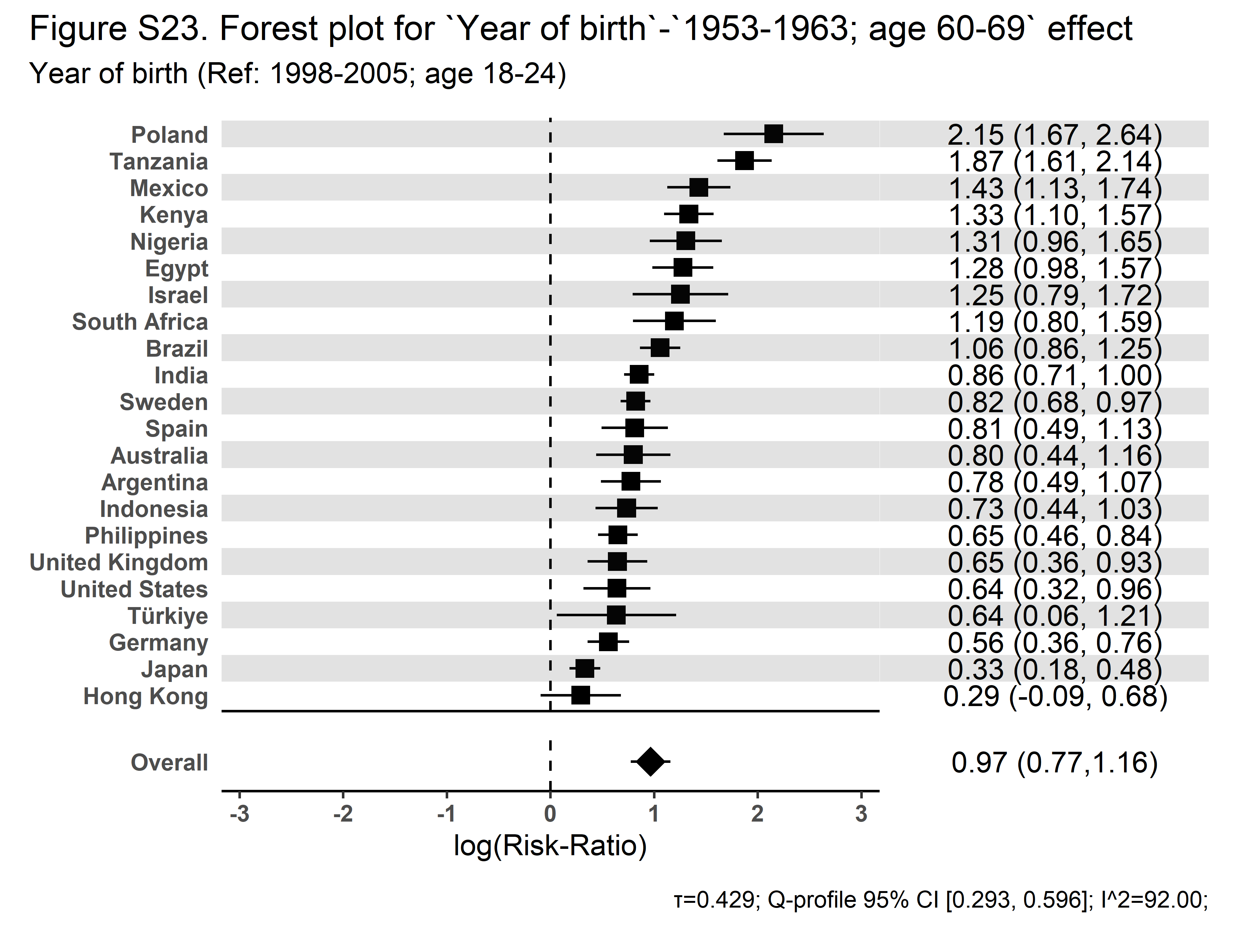

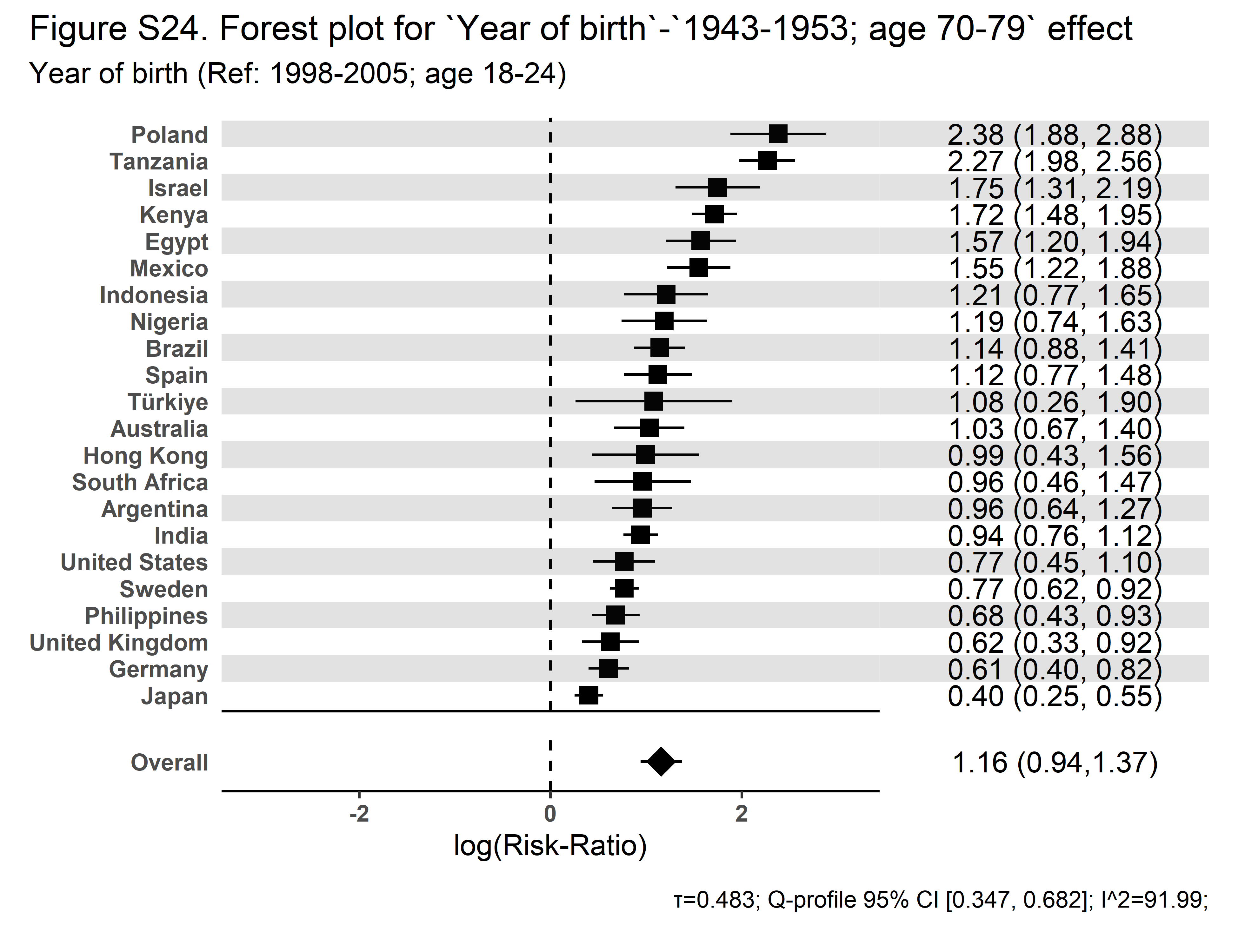

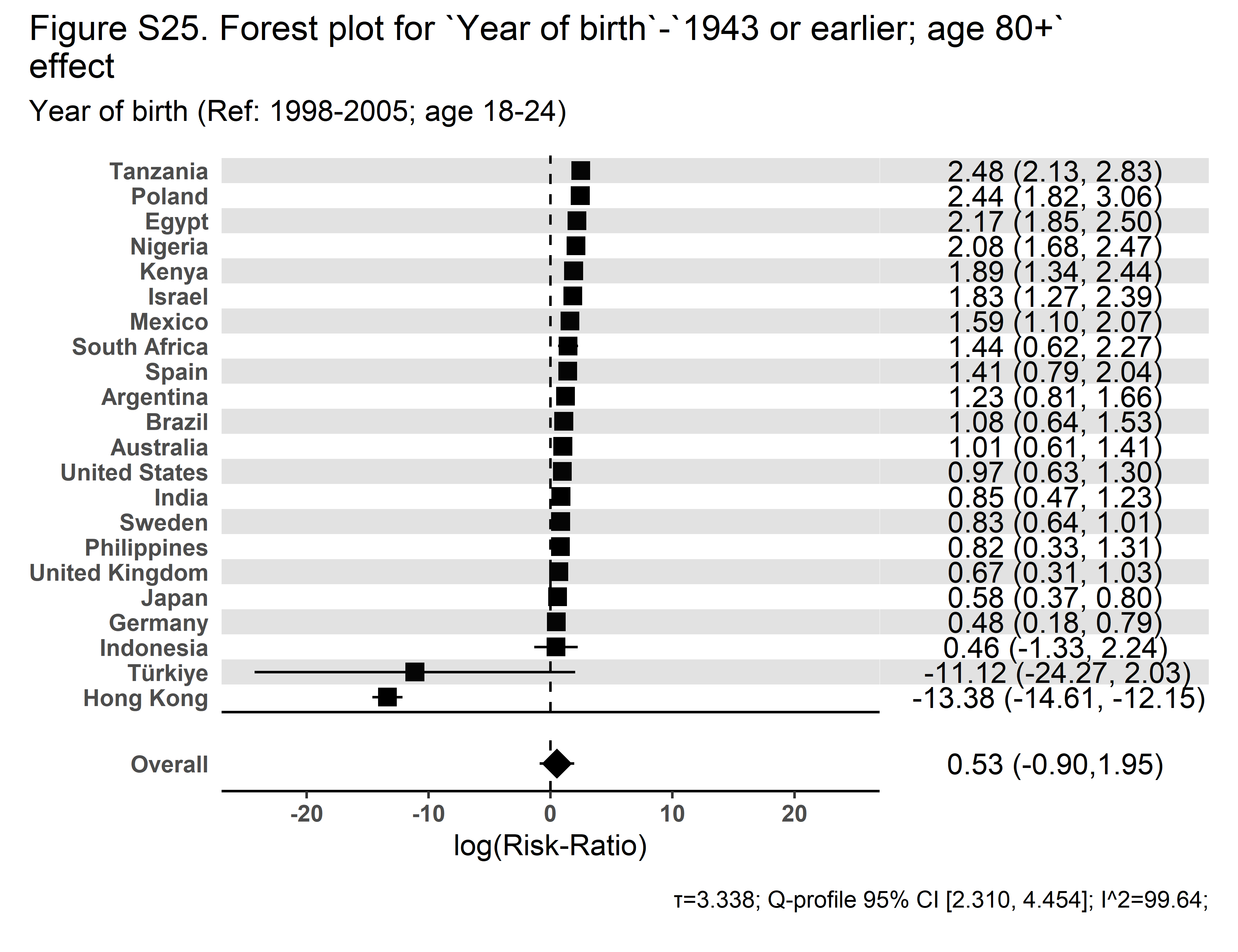

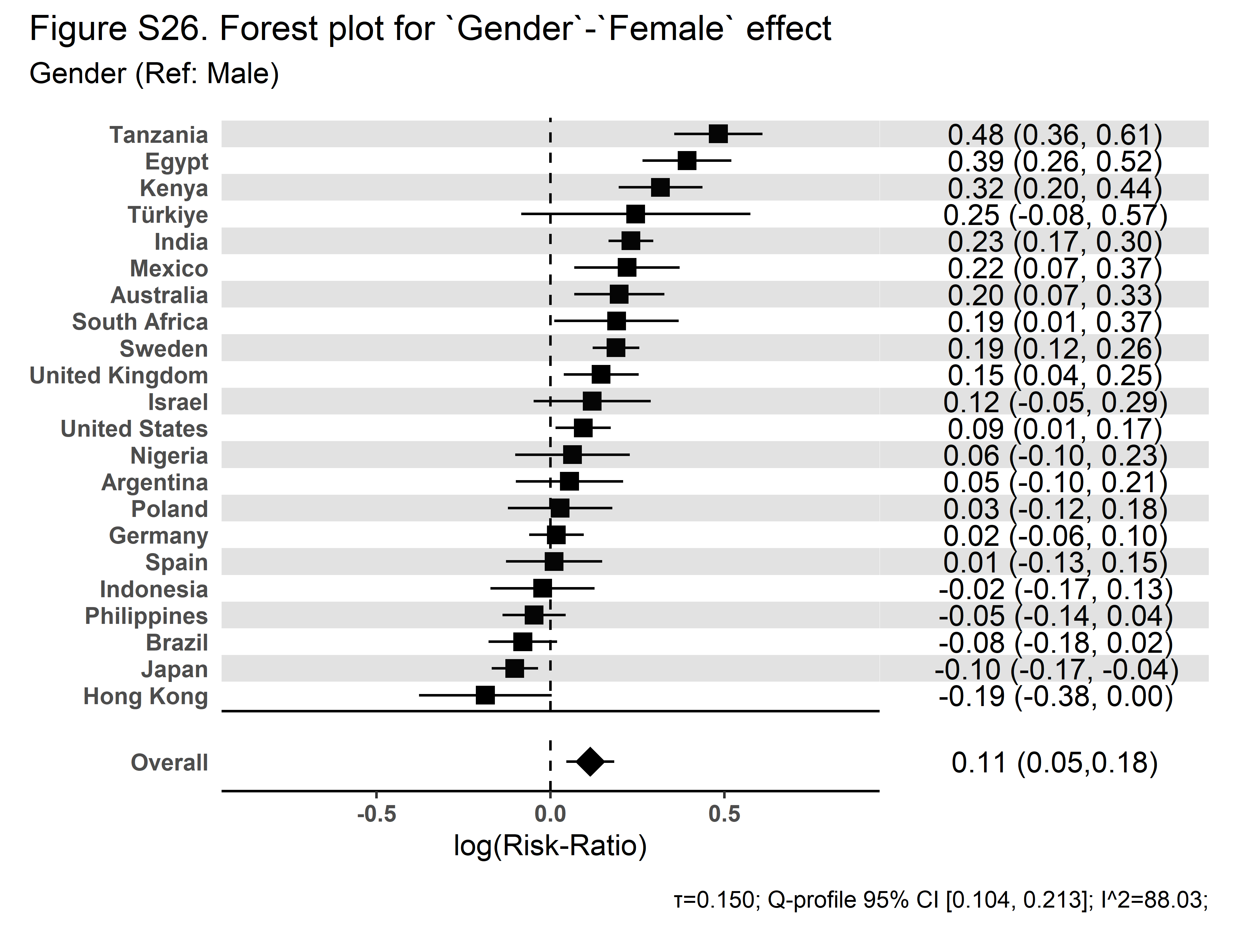

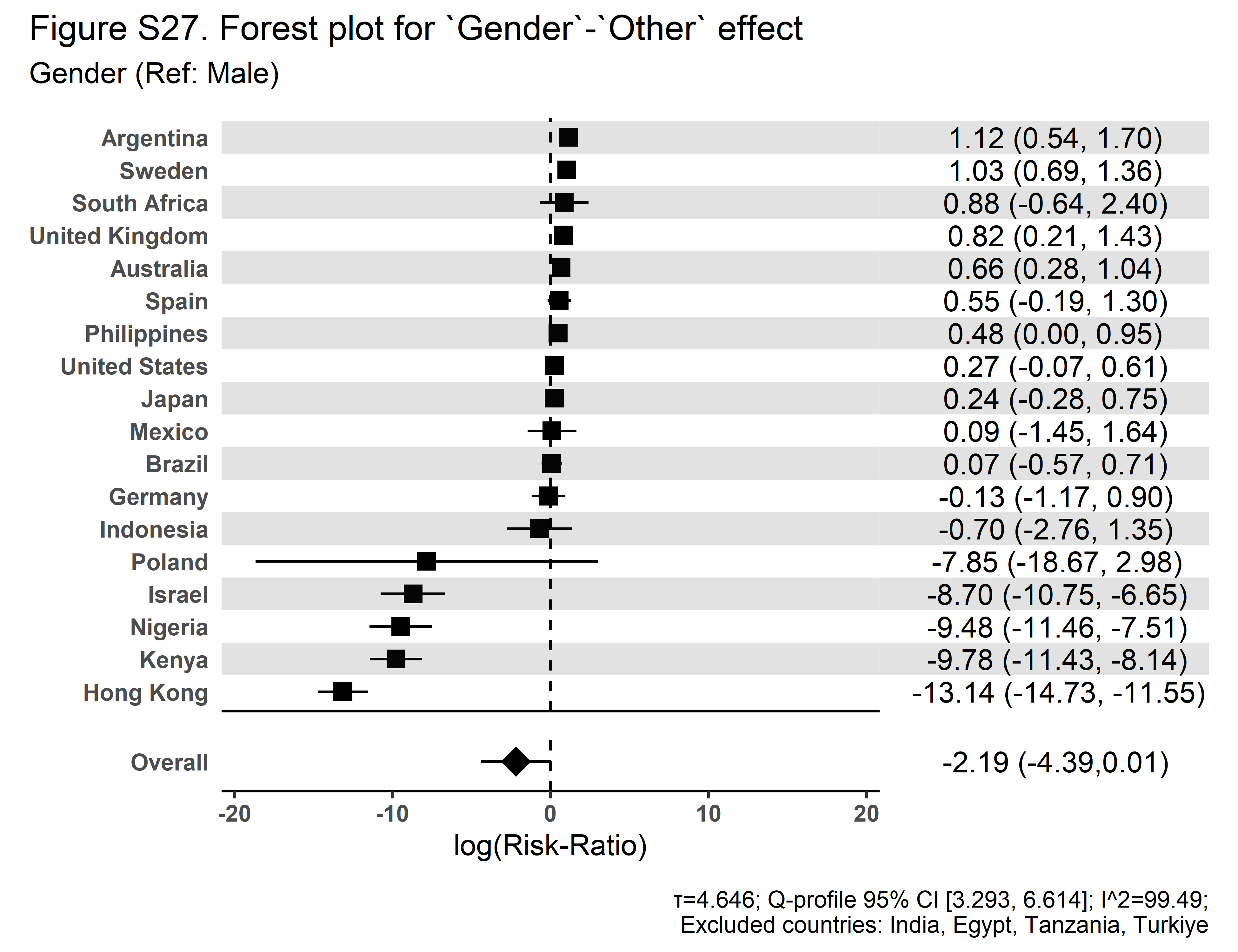

Supplement: Supplementary file 1 — Additional file 1: GFS Childhood Predictors of Health Problems in Adulthood Country Level Analyses [file 44263_2025_188_MOESM1_ESM.docx]
