# Supplementary material for: Childhood predictors of health limitations in life across 22 countries: a cross-national and cross-sectional analysis
Source: BMC Glob Public Health. 2025 Aug 13;3:70. doi: 10.1186/s44263-025-00188-0 (PMC12351978; doi:10.1186/s44263-025-00188-0)
Supplement: Supplementary file 2 — Additional file 2:STROBE checklist [file 44263_2025_188_MOESM2_ESM.docx]

Table 1: STROBE Checklist for GFS childhood predictors of health problems manuscript

|  | **Item No** | **Recommendation** | **Completed** |
| --- | --- | --- | --- |
| **Title and abstract** | 1 | (*a*) Indicate the study’s design with a commonly used term in the title or the abstract | X |
|  |  | (*b*) Provide in the abstract an informative and balanced summary of what was done and what was found | X |
| **Introduction** | | |  |
| Background/rationale | 2 | Explain the scientific background and rationale for the investigation being reported | X |
| Objectives | 3 | State specific objectives, including any prespecified hypotheses | X |
| **Methods** | | |  |
| Study design | 4 | Present key elements of study design early in the paper | X |
| Setting | 5 | Describe the setting, locations, and relevant dates, including periods of recruitment, exposure, follow-up, and data collection | X |
| Participants | 6 | (*a*) Give the eligibility criteria, and the sources and methods of selection of participants | X |
| Variables | 7 | Clearly define all outcomes, exposures, predictors, potential confounders, and effect modifiers. Give diagnostic criteria, if applicable | X |
| Data sources/ measurement | 8* | For each variable of interest, give sources of data and details of methods of assessment (measurement). Describe comparability of assessment methods if there is more than one group | X |
| Bias | 9 | Describe any efforts to address potential sources of bias | X |
| Study size | 10 | Explain how the study size was arrived at | X |
| Quantitative variables | 11 | Explain how quantitative variables were handled in the analyses. If applicable, describe which groupings were chosen and why | X |
| Statistical methods | 12 | (*a*) Describe all statistical methods, including those used to control for confounding | X |
|  |  | (*b*) Describe any methods used to examine subgroups and interactions | X |
|  |  | (*c*) Explain how missing data were addressed | X |
|  |  | (*d*) If applicable, describe analytical methods taking account of sampling strategy | X |
|  |  | (*e*) Describe any sensitivity analyses | X |
| **Results** | | |  |
| Participants | 13* | (a) Report numbers of individuals at each stage of study—eg numbers potentially eligible, examined for eligibility, confirmed eligible, included in the study, completing follow-up, and analysed | X |
|  |  | (b) Give reasons for non-participation at each stage | X |
|  |  | (c) Consider use of a flow diagram | Not done due to variation in sampling strategy noted in the manuscript. |
| Descriptive data | 14* | (a) Give characteristics of study participants (eg demographic, clinical, social) and information on exposures and potential confounders | X |
|  |  | (b) Indicate number of participants with missing data for each variable of interest | Not provided in this manuscript given size of the GFS. |
| Outcome data | 15* | Report numbers of outcome events or summary measures | X - provided for self-reported presence of health limtiations |
| Main results | 16 | (*a*) Give unadjusted estimates and, if applicable, confounder-adjusted estimates and their precision (eg, 95% confidence interval). Make clear which confounders were adjusted for and why they were included | Main results table includes the RR, CI, Estimated Proportion of Effectis by Threshold (<.9 and 1.1), I-2, and global p-value. |
|  |  | (*b*) Report category boundaries when continuous variables were categorized | Main outcome was binary |
|  |  | (*c*) If relevant, consider translating estimates of relative risk into absolute risk for a meaningful time period | Not relevant for time period. |
| Other analyses | 17 | Report other analyses done—eg analyses of subgroups and interactions, and sensitivity analyses | Country-specific tables are provided for cross country comparison as well as E-value for the estimates. |
| **Discussion** | | |  |
| Key results | 18 | Summarise key results with reference to study objectives | X |
| Limitations | 19 | Discuss limitations of the study, taking into account sources of potential bias or imprecision. Discuss both direction and magnitude of any potential bias | X |
| Interpretation | 20 | Give a cautious overall interpretation of results considering objectives, limitations, multiplicity of analyses, results from similar studies, and other relevant evidence | X |
| Generalisability | 21 | Discuss the generalisability (external validity) of the study results | X |
| **Other information** | | |  |
| Funding | 22 | Give the source of funding and the role of the funders for the present study and, if applicable, for the original study on which the present article is based | X |
